# Improved chromosome-level genome assembly of the Glanville fritillary butterfly (*Melitaea cinxia*) integrating Pacific Biosciences long reads and a high-density linkage map

**DOI:** 10.1093/gigascience/giab097

**Published:** 2022-01-12

**Authors:** Olli-Pekka Smolander, Daniel Blande, Virpi Ahola, Pasi Rastas, Jaakko Tanskanen, Juhana I Kammonen, Vicencio Oostra, Lorenzo Pellegrini, Suvi Ikonen, Tad Dallas, Michelle F DiLeo, Anne Duplouy, Ilhan Cem Duru, Pauliina Halimaa, Aapo Kahilainen, Suyog S Kuwar, Sirpa O Kärenlampi, Elvira Lafuente, Shiqi Luo, Jenny Makkonen, Abhilash Nair, Maria de la Paz Celorio-Mancera, Ville Pennanen, Annukka Ruokolainen, Tarja Sundell, Arja I Tervahauta, Victoria Twort, Erik van Bergen, Janina Österman-Udd, Lars Paulin, Mikko J Frilander, Petri Auvinen, Marjo Saastamoinen

**Affiliations:** Institute of Biotechnology, University of Helsinki, 00790 Helsinki, Finland; Department of Chemistry and Biotechnology, Tallinn University of Technology, 12618 Tallinn, Estonia; Organismal and Evolutionary Biology Research Programme, University of Helsinki, 00014 Helsinki, Finland; Organismal and Evolutionary Biology Research Programme, University of Helsinki, 00014 Helsinki, Finland; Ming Wai Lau Centre for Reparative Medicine, Karolinska Institutet, 171 77 Stockholm, Hong Kong; Institute of Biotechnology, University of Helsinki, 00790 Helsinki, Finland; Natural Resource Institute (LUKE), 00790 Helsinki, Finland; Institute of Biotechnology, University of Helsinki, 00790 Helsinki, Finland; Organismal and Evolutionary Biology Research Programme, University of Helsinki, 00014 Helsinki, Finland; Department of Evolution, Ecology and Behaviour, University of Liverpool, Liverpool CH64 7TE, UK; Institute of Biotechnology, University of Helsinki, 00790 Helsinki, Finland; Organismal and Evolutionary Biology Research Programme, University of Helsinki, 00014 Helsinki, Finland; Department of Biological Sciences, Louisiana State University, Baton Rouge, LA 70803, USA; Organismal and Evolutionary Biology Research Programme, University of Helsinki, 00014 Helsinki, Finland; Organismal and Evolutionary Biology Research Programme, University of Helsinki, 00014 Helsinki, Finland; Department of Biology, Lund University, 223 62 Lund, Sweden; Institute of Biotechnology, University of Helsinki, 00790 Helsinki, Finland; Department of Environmental and Biological Sciences, University of Eastern Finland, 70211 KUOPIO, Finland; Organismal and Evolutionary Biology Research Programme, University of Helsinki, 00014 Helsinki, Finland; Department of Entomology and Nematology, University of Florida, Gainesville, FL 32611-0620, USA; Department of Zoology, Loknete Vyankatrao Hiray Arts, Science & Commerce College, 422003, Maharashtra, India; Department of Environmental and Biological Sciences, University of Eastern Finland, 70211 KUOPIO, Finland; Department of Aquatic Ecology, Swiss Federal Institute of Aquatic Science and Technology, CH-8600 Dübendorf, Switzerland; College of Plant Protection, China Agricultural University, Beijing 100083, China; Department of Environmental and Biological Sciences, University of Eastern Finland, 70211 KUOPIO, Finland; Organismal and Evolutionary Biology Research Programme, University of Helsinki, 00014 Helsinki, Finland; Department of Zoology, Stockholm University, 106 91 Stockholm, Sweden; Viikki Plant Science Centre, Organismal and Evolutionary Biology Research Programme, University of Helsinki, 00014 Helsinki, Finland; Organismal and Evolutionary Biology Research Programme, University of Helsinki, 00014 Helsinki, Finland; Institute of Biotechnology, University of Helsinki, 00790 Helsinki, Finland; Department of Environmental and Biological Sciences, University of Eastern Finland, 70211 KUOPIO, Finland; Department of Biology, Lund University, 223 62 Lund, Sweden; Organismal and Evolutionary Biology Research Programme, University of Helsinki, 00014 Helsinki, Finland; Organismal and Evolutionary Biology Research Programme, University of Helsinki, 00014 Helsinki, Finland; Institute of Biotechnology, University of Helsinki, 00790 Helsinki, Finland; Institute of Biotechnology, University of Helsinki, 00790 Helsinki, Finland; Institute of Biotechnology, University of Helsinki, 00790 Helsinki, Finland; Organismal and Evolutionary Biology Research Programme, University of Helsinki, 00014 Helsinki, Finland; Helsinki Institute of Life Science (HiLIFE), University of Helsinki, 00014 Helsinki, Finland

**Keywords:** *Melitaea cinxia*, Glanville fritillary, genome, spatial ecology

## Abstract

**Background:**

The Glanville fritillary (*Melitaea cinxia*) butterfly is a model system for metapopulation dynamics research in fragmented landscapes. Here, we provide a chromosome-level assembly of the butterfly's genome produced from Pacific Biosciences sequencing of a pool of males, combined with a linkage map from population crosses.

**Results:**

The final assembly size of 484 Mb is an increase of 94 Mb on the previously published genome. Estimation of the completeness of the genome with BUSCO indicates that the genome contains 92–94% of the BUSCO genes in complete and single copies. We predicted 14,810 genes using the MAKER pipeline and manually curated 1,232 of these gene models.

**Conclusions:**

The genome and its annotated gene models are a valuable resource for future comparative genomics, molecular biology, transcriptome, and genetics studies on this species.

## Data Description

### Context

Identifying and characterizing genes underlying ecologically and evolutionarily relevant phenotypes in natural populations has become possible with novel genomic tools that can also be used in “non-model” organisms. The Glanville fritillary (*Melitaea cinxia*, NCBI:txid113334) butterfly, and in particular its metapopulation in the Åland Islands (southwest Finland), is an ecological model system in spatial ecology [1, 2]. In Åland, the species inhabits a network of dry outcrop meadows and pastures and persists as a classic metapopulation with high turnover in patch occupancy [1]. The network of 4,500 potential habitat patches has been systematically surveyed bi-annually for butterfly occupancy and abundance since 1993 [3], providing a vast amount of ecological data on population dynamics [2]. Experimental manipulations under more controlled conditions are also possible owing to the small size, high fecundity, and relatively short generation time of the species. Consequently, our understanding of the species includes knowledge of life history variation across development stages [4, 5], dispersal dynamics [6, 7], species interactions with host plants and parasitoids [8–12], and stress tolerance [13, 14]. During the past decade, the system has also been used to study genetic and evolutionary processes, such as identifying candidate genes underlying variation and evolution of dispersal in fragmented habitats [15] and host plant preference [16], and assessing allelic variation and its dynamics in space and time [17–19]. Several approaches have been used to explore the genetic underpinnings of phenotypic variation in the Glanville fritillary metapopulation, ranging from candidate gene approaches [13, 20] and quantitative genetics [21, 22] to whole-genome scans [23, 24], under both laboratory and natural environmental conditions.

The first *M. cinxia* genome assembly was released in 2014 [25]. This genome was produced from a combination of 454 sequencing for contig assembly, followed by scaffolding with Illumina paired-end (PE), SOLiD mate-pair reads and Pacific Biosciences (PacBio) data. The size of the final assembly was 390 Mb made up from 8,261 scaffolds, with a scaffold N50 of 119,328. Scaffolds were assigned to chromosomes on the basis of a linkage map produced from RAD sequencing [25]. We recently assessed the actual genome size using a *k*-mer–based approach on Illumina sequencing data and obtained estimates ranging from 488 to 494 Mb (Supplementary File S5). It was considered that a new genome, sequenced using PacBio long reads, would result in a more complete assembly and better represent the repetitive areas of the genome.

Here, a new sequencing and assembly of the *M. cinxia* genome has been carried out using a pool of 7 male butterflies from a single larval family collected from Sottunga, an island in an eastern part of the archipelago. Sequencing was conducted using the PacBio RSII sequencer. An initial assembly was created using FALCON [27, 28] followed by polishing performed with Quiver [27]. A new linkage map was created and used to assign the assembled scaffolds to their correct positions and orientations within the 31 chromosomes. The scaffolds were then gap-filled, producing a final assembly of 484 Mb with a scaffold N50 of 17,331,753 bp. The obtained genome size is well in line with the *k*-mer estimates. Gene prediction on the genome assembly was carried out using MAKER v 2.31.10 [29], which was run iteratively using several independent training sets. Manual annotation was performed for 1,232 of the gene models. The genome assembly increased greatly in contiguity and completeness compared to the first genome (Table 1), with chromosomal superscaffold N50 values of 17,331,753 bp in the new genome compared to 119,328 bp in the Version 1 genome.

**Table 1:** Assembly statistics

Statistic	*M. cinxia*		*Bombyx mori*	*Pieris napi* v1.1
	Version 2	Version 1 Scaffolds		
Length (bp)	484,462,241	389,907,520	460,334,017	349,759,982
N (%)	<0.01	7.42	0.10	22.47
Scaffold count	31	8,261	696	2,969
Longest scaffold (bp)	22,190,643	668,473	21,465,692	15,427,984
Scaffold N50 length (bp)	17,331,753	119,328	16,796,068	12,597,868
Scaffold N50 count (L50)	13	970	13	13
Contig count	529	48,180	726	53,510
Contig N50 length (bp)	1,831,849	14,057	12,201,325	10,538
Contig N50 count (L50)	79	7,366	16	6,914

Assembly statistics were calculated for the *M. cinxia* Version 2 genome, *M. cinxia* Version 1 scaffolds, and *B. mori* using the assembly-stats program v 17.02 [30]. Statistics for *H. melpomene* v2.5 and *P. napi* v1.1 were obtained from LepBase [31].

The significant increase in assembly size warrants a further investigation of the composition of these added sequences. Initial observations of individual alignments from genome-to-genome alignment show many collapsed repeat regions in the Version 1 genome that are mapped to multiple chromosomes in Version 2.

## Methods

**Figure 1:**
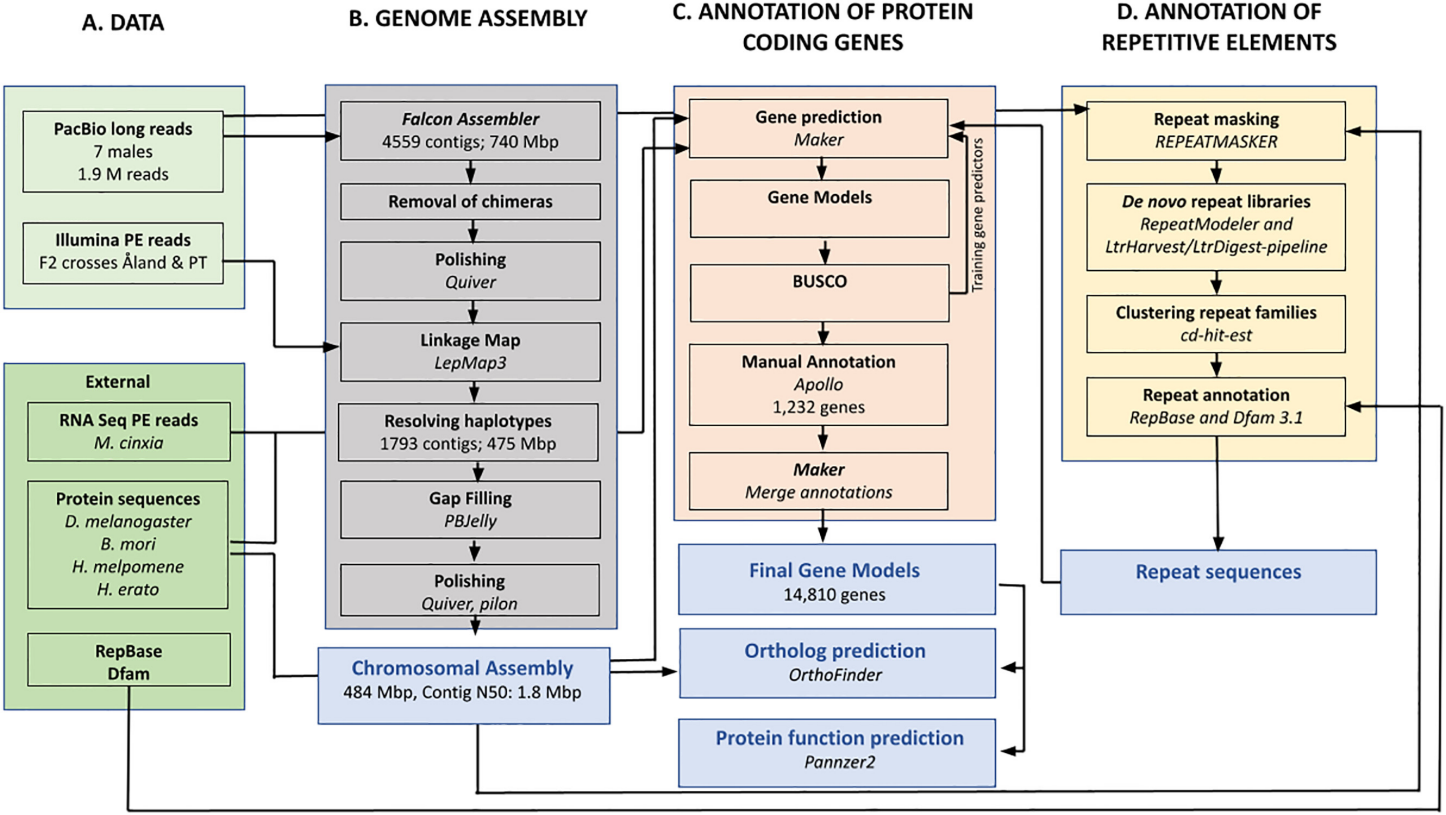
An overview of the assembly and annotation process of the improved Glanville fritillary genome.

An overview of the processing pipeline for the work is shown in Fig. 1.

### Genomic samples and DNA extraction

Owing to the facultatively univoltine life cycle of the butterfly in Finland, experimental inbreeding of the species would have taken several years. Therefore, we chose to sample individuals from an island population, Sottunga, expected to harbour lower genetic diversity compared to less isolated populations. Sottunga is part of the Åland Islands archipelago in the northern Baltic Sea, and the population was introduced here in 1991 using individuals collected on the mainland of Åland Island [32]. This introduction was carried out with 71 larval families. The distance to the nearest *M. cinxia* population across the water is 5 km, and we therefore assume that the introduced population has remained (almost) completely isolated. Furthermore, the effective population size of *M. cinxia* in Sottunga has been very low during the past 24 years (on average 57 larval nests/year in 1993–2019), and it has experienced several strong bottlenecks [33]. Using genomic markers, Fountain et al. [17] demonstrated that samples from the Sottunga population separate clearly from samples collected on the mainland.

During the fall survey of 2014 (see Ojanen et al. for details of the survey [3]) we collected individuals from 1 larval group on the island of Sottunga (patch No. 1439, 60 8.1768 N 20 40.1214 E). The larvae were collected once they were in diapause and most likely comprise full siblings [18]. The larval group was kept in diapause (+5°C) until the following spring and then reared to adulthood under common garden conditions (28:8°C; 12L:12D) at the Lammi Biological Station, University of Helsinki. After eclosion, butterflies were sexed and stored at −80°C. High molecular weight DNA was isolated from 7 adult males using the caesium chloride method [25]. Several individuals were used to obtain enough starting material for constructing the Single Molecule, Real-Time (SMRT) sequencing library.

### SMRT sequencing libraries and sequencing

Library construction for PacBio sequencing (PacBio RS II Sequencing System, RRID:SCR_017988) was carried out using the protocols recommended by the manufacturer (Pacific Biosciences, Menlo Park, CA, USA). Genomic DNA was sheared using a Megaruptor (Diagenode, Seraing, Belgium) followed by damage repair, end repair, hairpin ligation, and size selection using BluePippin (Sage Science, Beverly, MA, USA; RRID:SCR_020505). After primer annealing and polymerase binding, the DNA templates were sequenced on a PacBio RSII sequencer using P6/C4 chemistry and 360 min video time at the DNA Sequencing and Genomics Laboratory, Institute of Biotechnology, University of Helsinki, Finland [34].

### Genome Assembly

The genome was assembled using the FALCON assembler (FALCON-Integrate-1.8.6) [26, 27] with a read length cut-off of 18,000 bp. This cut-off was found to give the best contiguity for the assembly based on N50 value, while minimizing the percentage of possibly erroneous contigs. The erroneous contigs were detected by mapping markers of the linkage map from the previously published genome [25] to contigs and calculating the percentage of chimeric contigs. We tested 3 different read length cut-offs, 16,000, 18,000, and 20,000 bp, all of which included ∼9% of chimeric contigs. The assembly was based on 1.9M PacBio reads, 24.4 Gb in total, with an N50 of 18,479 bp, which is ∼50× coverage based on the final genome size. With the selected read cut-off the data produced 10.8 Gb of corrected reads that were further assembled using the FALCON software (Falcon, RRID:SCR_016089). The assembly yielded 4,559 primary contigs containing 739.9 Mb with an N50 of 340 kb and 1,661 alternative contigs containing 118.1 Mb with an N50 of 85,246 bp. The alternative contigs were automatically separated by the FALCON pipeline. The data were also assembled using miniasm software (0.2-r137-dirty) [35], which yielded similar results. The larger than expected initial assembly size, ∼1.5 times the *k*-mer estimate, is due to the multiple haplotypes originating from the 7 individuals used in sequencing.

To evaluate the putative chimeric contigs and assembly errors suggested by the genetic map, the raw SMRT sequencing data were mapped to the assembly primary contigs using BWA (BWA-0.7.17, RRID:SCR_010910) with the MEM algorithm [36]. The alignments of the 425 regions discovered as possibly chimeric were visually inspected. Of these regions, 92 showed even read coverage and no evident signs of assembly errors, while 333 regions contained areas with low coverage and/or repeat regions indicated by high coverage that had led to erroneous overlaps and misassemblies. These errors were identified by positions where the majority of the reads did not fully align; i.e., the alignments ended mid-read. The assembly was split in the positions where the coverage was at minimum. The resulting assembly was polished using the SMRT sequencing data and Quiver [26] software from the SMRT Tools-package (PacBio).

### Linkage Map

Linkage mapping was constructed from whole-genome resequencing data of F2 crosses of *M. cinxia*. The grandparents of these F2 crosses are offspring of wild-collected *M. cinxia* originating from 2 distantly related *M. cinxia* populations around the Baltic Sea: the Åland Islands (ÅL) [1] and Pieni Tytärsaari (PT) populations [37]. Between-population crosses of type ÅL♂×PT♀ and ÅL♀×PT♂ were established to create the F1 population. Some of these F1 individuals were used to establish the F2 families, actively avoiding mating among siblings. A subset of the resulting full-sibling families were reared to adulthood, and 5 of these F2 families, together with their parents and grandparents, were selected for resequencing. In total, resequencing included 10 grandparental individuals, 10 F1 parents, and 165 F2 individuals (N: 185).

All the larvae from different generations completed development under common garden conditions (28:15°C; 12L:12D) utilizing fresh leaves of greenhouse-grown *Veronica spicata*. Diapausing larvae were kept in a growth chamber at +5°C and 80% relative humidity for ∼7 months to mimic the normal wintertime conditions for these butterflies. Adults were kept in hanging cages (of 50 cm height and 40 cm diameter) at ∼26:18°C; 9L:15D and fed *ad libitum* with 20% honey-water solution throughout the experiments.

Before DNA extraction the adult butterflies were stored at −80°C, and either thorax or abdomen tissue of these individuals was used for sequencing. Tissues were homogenized prior to extraction using TissueLyser (Qiagen, Venlo, The Netherlands) at 30/s for 1.5 mins with Tungsten Carbide Beads, 3 mm (Qiagen, Venlo, The Netherlands), and ATL buffer (Qiagen, Venlo, The Netherlands). DNA was extracted using the NucleoSpin 96 Tissue Core Kit (Macherey-Nage, Düren, Germany) according to the manufacturer's protocol with the exception that lysing time was extended to overnight. The samples were additionally treated with RNase A (Thermo Fisher Scientific, Waltham, MA. USA) before sequencing. Sequencing was performed using standard PE library preparation and Illumina HiSeq 2000 (Illumina HiSeq2000, RRID:SCR_020132) with 125 bp PE reads.

The mapping procedure followed the Lep-MAP3 [26] pipeline (biotools:lep-map3). First, individual fastq files were mapped to the contig assembly using BWA MEM (BWA-0.7.17) [36] and individual bam files were created using SAMtools (1.6) (SAMTOOLS, RRID:SCR_002105) [38, 39]. SAMtools mpileup and the scripts pileupParser2.awk and pileup2posterior.awk were used to obtain input data for Lep-MAP3. Then ParentCall2 (parameter: ZLimit: 2) and Filtering2 (parameters: dataTolerance: 0.0001; removeNonInformative: 1; familyInformativeLimit: 4) were run to obtain data with ≥4 informative families for each marker, resulting in a final input with almost 2.5M markers.

SeparateChromosomes2 was run on the final data (parameters lodLimit: 20; samplePair: 0.2;numThreads: 48) to obtain 31 linkage groups with a total of 2.4M markers. OrderMarkers2 was run (parameter recombination2: 0) on each linkage group (chromosome). This map was used to anchor the contig assembly into chromosomes. To validate anchoring, the map construction was repeated in the same way except that OrderMarkers2 was run on the physical order of markers to reduce noise in the linkage map. Finally, the raw data were remapped to the gap-filled chromosome-level assembly and the linkage map was redone in the new physical order to infer final recombination rates.

### Anchoring the genome and resolving haplotypes using the linkage map

The contigs were aligned against each other and lift-over chains were created by running the first 2 steps (batch A and B to calculate the alignment chain) of the HaploMerger2 [40] pipeline. By manually inspecting this chain (all.chain.gz), contigs fully contained in some longer contig were removed. Initial contig order and orientation within each chromosome was calculated by the median map position of each contig and the longest increasing subsequence of markers, respectively. For each chromosome, Marey map [41], a scatter plot of physical and linkage positions combining the genetic and physical maps, and contig-contig alignments from the chain were recorded. The contigs’ orders and orientations were manually fixed when needed if the map had support for alternative orientation. If the contig-contig alignments linked contigs together, they were joined. Any assembly errors that were found were corrected by splitting the contigs accordingly. Also, partially haplotypic contigs were found and collapsed, i.e., alternative haplotype sequence removed, on the basis of the Marey maps and contig-contig alignments. This manual work facilitated the removal of additional haplotype contigs and regions and resulted in the haploid reference genome sequence including start and end positions of contigs in the correct order and orientation for each chromosome. Of 2,933 contigs in initial reference, 4 were chimeric and were split to 9 separate contigs. Of the resulting 2,938 contigs, 1,080 were included without any modification, 825 were trimmed on 1 or both ends, and 1,033 were completely contained and thus removed. Finally, the haplotype-corrected genome was gap-filled using PBJelly software (PBSuite_15.8.24; RRID:SCR_012091) [42] with the original SMRT sequencing data and polished with the Quiver tool [26] from the SMRT Tools-package 2.3.0 (PacBio) and with Pilon (1.21) (Pilon, RRID:SCR_014731) [43], which resulted in the final reference genome sequence of ∼484 Mb.

**Figure 2:**
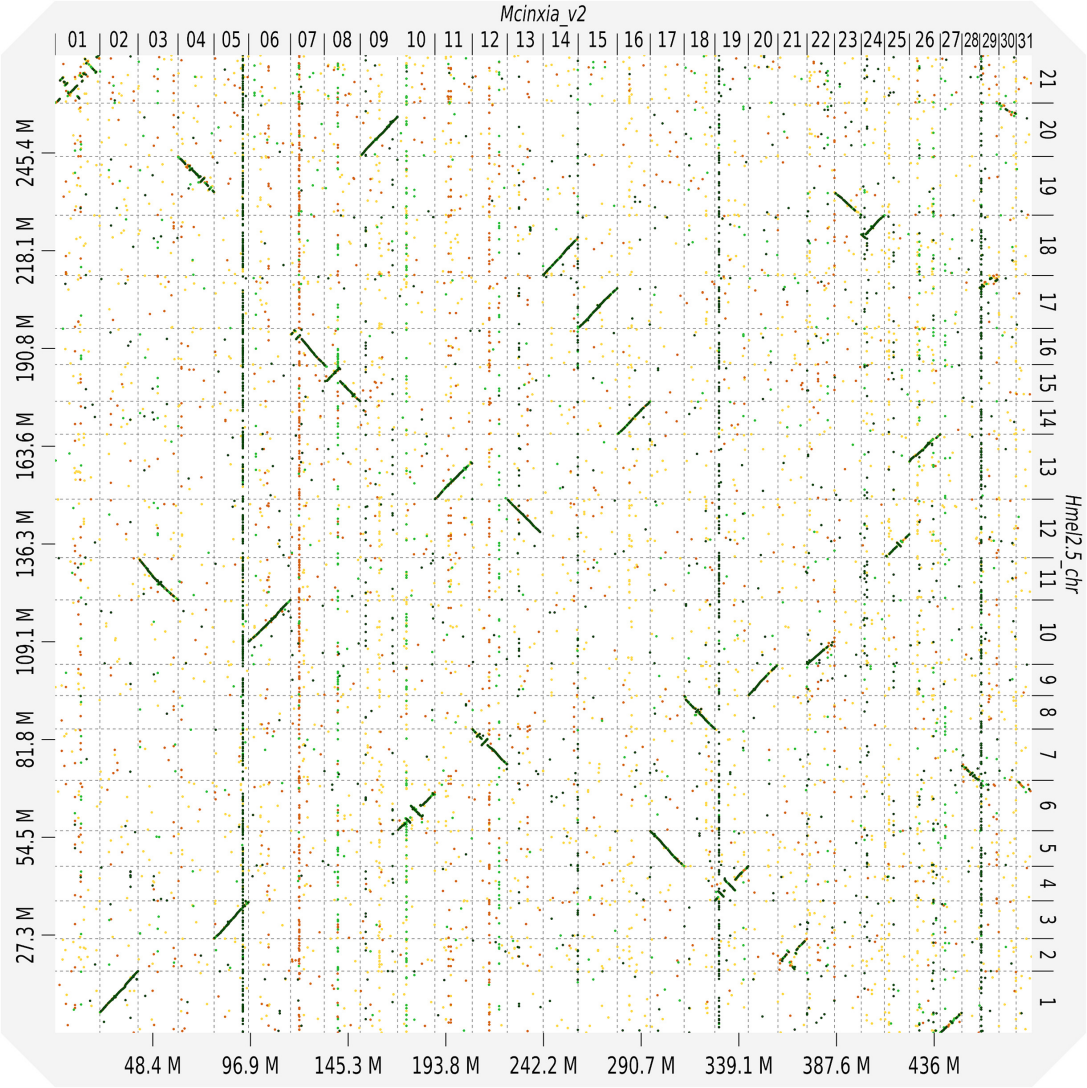
A dot-plot structural comparison of the *H. melpomene* genome against the *M. cinxia* v2 genome. The alignment was created using D-GENIES (1.2.0) [48]. The diagonal lines indicate the collinearity between the 2 species. The lack of collinearity in sex chromosomes is visible in the upper left corner between Mcnxia_v2 chr 01 and Hmel2.5 chr 21. The visible vertical lines show repeats that are resolved in Mcinxia_v2 but are present in all chromosomes in Hmel2.5_chr.

The chromosomes were aligned against the *Heliconius melpomene* (2.5) [44, 45] and *Pieris napi* [46] genomes using the LAST aligner(938) [47] to check structural similarity between the species (Supplementary Figs S1–S13). An overview alignment for *H. melpomene* was created using D-GENIES (1.2.0) (D-GENIES, RRID:SCR_018967) [48] (Fig. 2). The data show a high level of collinearity between *M. cinxia* and *H. melpomene* chromosomes, as described before in Ahola et al. [25]. An interesting point is the lack of collinearity with sex chromosomes (*M. cinxia* chromosome 1 and *H. melpomene* chromosome 21). Furthermore, the visible vertical lines show the effect of long-read assembly on repeat resolution. With long reads spanning the repeats and allowing their accurate placement in the contigs, in *M. cinxia* the repeats are placed in single chromosomes whereas in *H. melpomene* they are present in all chromosomes.

### Repeat masking and annotation

Genomic assemblies were masked with *de novo* repeat libraries by RepeatMasker v.4.0.9 (RepeatMasker, RRID:SCR_012954) [49]. *De novo* repeat libraries were constructed from original PacBio reads with lengths >30,000 bp and assembled scaffolds (pseudo chromosomes) using RepeatModeler v 1.0.10 (RepeatModeler, RRID:SCR_015027) [50] and the LtrHarvest/LtrDigest-pipeline [51, 52]. Repeat families were clustered using cd-hit-est applying the 80/80-rule (80% identity over 80% length) [53]. Repeat annotations were confirmed by RepBase Release 20,181,026 [54] and Dfam version 3.1 [55].

### Transcriptome assembly

To aid construction of gene models, we capitalized on 2 transcriptome assemblies that were produced as part of separate projects in our laboratory to be presented in upcoming publications ([5], PRJNA670126). Importantly for gene model construction, they represent a wide range of transcriptional diversity, as the RNA-seq data are derived from various developmental stages (first instar larvae, fourth instar larvae, and adult thorax and abdomen). All individuals were lab-reared but originated from the same butterfly metapopulation. Transcriptome 1 was produced using a set of 78 individually sequenced female larvae (fourth developmental instar) [5], sequenced to an average depth of 17.3M reads (read lengths 85 and 65 bp for forward and reverse PE reads, respectively). Because the 2 sexes are practically indistinguishable in the larval stages, the females were identified on the basis of homozygosity across a set of 22 Z-chromosome–specific single-nucleotide polymorphism loci [5]. To remove Illumina adapter sequences, we trimmed raw reads using Trimmomatic (Trimmomatic-0.35, RRID:SCR_011848) [56], and normalized using Trinity v2.6.5 (Trinity, RRID:SCR_013048) [57]. We then used 2 separate procedures to construct *de novo* transcriptome assemblies, Trinity (v2.6.5) and Velvet/Oases (1.2.10) [58]. Trinity was run with standard settings, whereas Velvet/Oases used a range of 7 *k*-mer sizes (21–71 bp), producing a separate assembly for each *k*-mer size. We then combined the resulting assemblies, filtered the combined assembly using the EvidentialGene (tr2aacds.pl VERSION 2017.12.21) [59] pipeline, and removed contigs smaller than 200 bp or expressed at a low level (<1 normalized counts per million), yielding the final assembly. Transcriptome 2 was constructed from a set of 12 adult females (thorax and abdomen, without ovaries) and 48 first instar larvae, as part of a separate gene expression study (PRJNA670126). RNA from these 60 individual samples was sequenced to an average depth of 16.6M reads (86/74 bp PE). The stranded RNA-seq libraries were made using Ovation® Universal RNA-Seq System (Nugen) with custom ribosomal RNA removal. The libraries were PE sequenced on a NextSeq 500 using the 150 bp kit (Illumina) at the DNA sequencing and genomics laboratory Institute of Biotechnology University of Helsinki. We trimmed the reads using fastp (v0.20.0) [60], and used the HISAT2 2.0.4 (HISAT2, RRID:SCR_015530)/StringTie 1.3.5 (StringTie, RRID:SCR_016323) pipeline [61] to construct a genome-guided transcriptome assembly, mapping the RNA-seq reads to the new genome assembly. Transcriptome 1 yielded 69,182 putative transcripts with mean length of 727 bp (95% CI: 206–3,433), while Transcriptome 2 yielded 137,250 putative transcripts with mean length of 1,737 (95% CI: 203–9,106). These statistics should be interpreted with caution because the assemblies derive from different life stages, and different assembly and filtering approaches were used (reflecting differences in histories of the datasets as they were produced for different projects).

### Gene model annotation

Initial gene predictions were obtained by running the MAKER v 2.31.10 [29] gene prediction program in an iterative procedure. In the first round of MAKER, transcriptome assembly 1, described above, was provided as evidence, and genes were predicted solely from the aligned transcripts. This resulted in 14,738 gene models. These gene models were then used for training the SNAP (2013–02-16) [62] and AUGUSTUS (3.3.2) (Augustus, RRID:SCR_008417) [63] gene predictors. A second round of MAKER was run providing the *de novo* transcripts from both transcriptomes (see previous paragraph), trained gene prediction models, repeat masking file, and protein data from other lepidopteran species. The MAKER settings were adjusted to allow prediction of gene models without requiring a corresponding transcript in the *de novo* transcriptome assembly. Following each round of MAKER gene prediction, the annotation completeness was assessed using BUSCO (Benchmarking Universal Single-Copy Orthologs, RRID:SCR_015008) [64, 65].

### Manual annotation

Manual annotation was performed for 1,232 genes, using the Apollo collaborative annotation system Version 2.1.0 [66]. The collaborative annotation environment was set up in Ubuntu Linux 14.04 server with 250 GB RAM and 48 AMD Opteron 6 168 processing cores. This was later upgraded to a cloud server provided by the Finnish IT Center for Science (CSC) and run on Ubuntu Linux 18.04 with 200 GB RAM and 40 Intel Xeon model 85 processing cores. Evidence tracks were produced containing gene predictions from 3 rounds of MAKER, RNA-seq alignments of sequence reads, and protein alignments from other species (Table 2). RNA-seq alignments comprised a mixed-tissue pooled sample, an abdomen pooled sample, and 6 larval samples (from Transcriptome 1) selected to represent a diverse range and included, e.g., both sexes and different family backgrounds. A list of gene families that were considered of particular interest in butterfly research were identified for prioritization during the manual annotation. (Supplementary File S4). The gene annotators were able to select a family of genes for annotation, or a random selection from the prioritized families was given. Gene models were corrected by examining the evidence tracks in the browser, conducting blast searches, and examining multiple alignments of protein sequences. In total for the 1,232 genes, 1,455 messenger RNAs (mRNAs) were manually inspected, of which 814 genes and mRNAs were changed. Most changes were made to exon borders and mRNA exon structure, especially in the case of multiple isoforms.

**Table 2:** Evidence tracks that were used during the manual annotation of 1,232 *M. cinxia* genes

Evidence track	Type	Description
Maker 1	Gene prediction	Initial maker gene predictions based on EST alignments
Maker 2	Gene prediction	Second round of gene predictions from EST alignments, protein alignments, and gene predictors trained on maker 1.
RNA-seq abdomen pool	RNA-seq alignment	RNA-seq reads aligned to the genome with STAR [67]
RNA-seq mixed-tissue pool	RNA-seq alignment	
*B. mori* proteins	Protein alignment	Proteins sequences aligned to the genome with AAT
*H. melpomene* proteins	Protein alignment	
*Drosophila melanogaster* proteins	Protein alignment	
*Heliconius erato* proteins	Protein alignment	
RNA-seq female larvae family 80	RNA-seq alignment	RNA-seq reads aligned to the genome with STAR [67]
RNA-seq female larvae family 70	RNA-seq alignment	
RNA-seq female larvae family 119	RNA-seq alignment	
RNA-seq female larvae family 120	RNA-seq alignment	
RNA-seq male larvae family 80	RNA-seq alignment	
RNA-seq male larvae family 119	RNA-seq alignment	

### Final gene models

Following the manual annotation, the SNAP [62] and AUGUSTUS [63] gene predictors were retrained using the manually annotated gene models. MAKER was run using the updated gene predictors, Transcriptome 1 and 2, and using a masking file for repeats. As a final step to incorporate the manually annotated gene models, MAKER was run, providing the previous MAKER file to pred_gff and the manually annotated models to model_gff. Gene functional prediction was performed using Pannzer v2 [68].

### Ortholog identification

**Figure 3:**
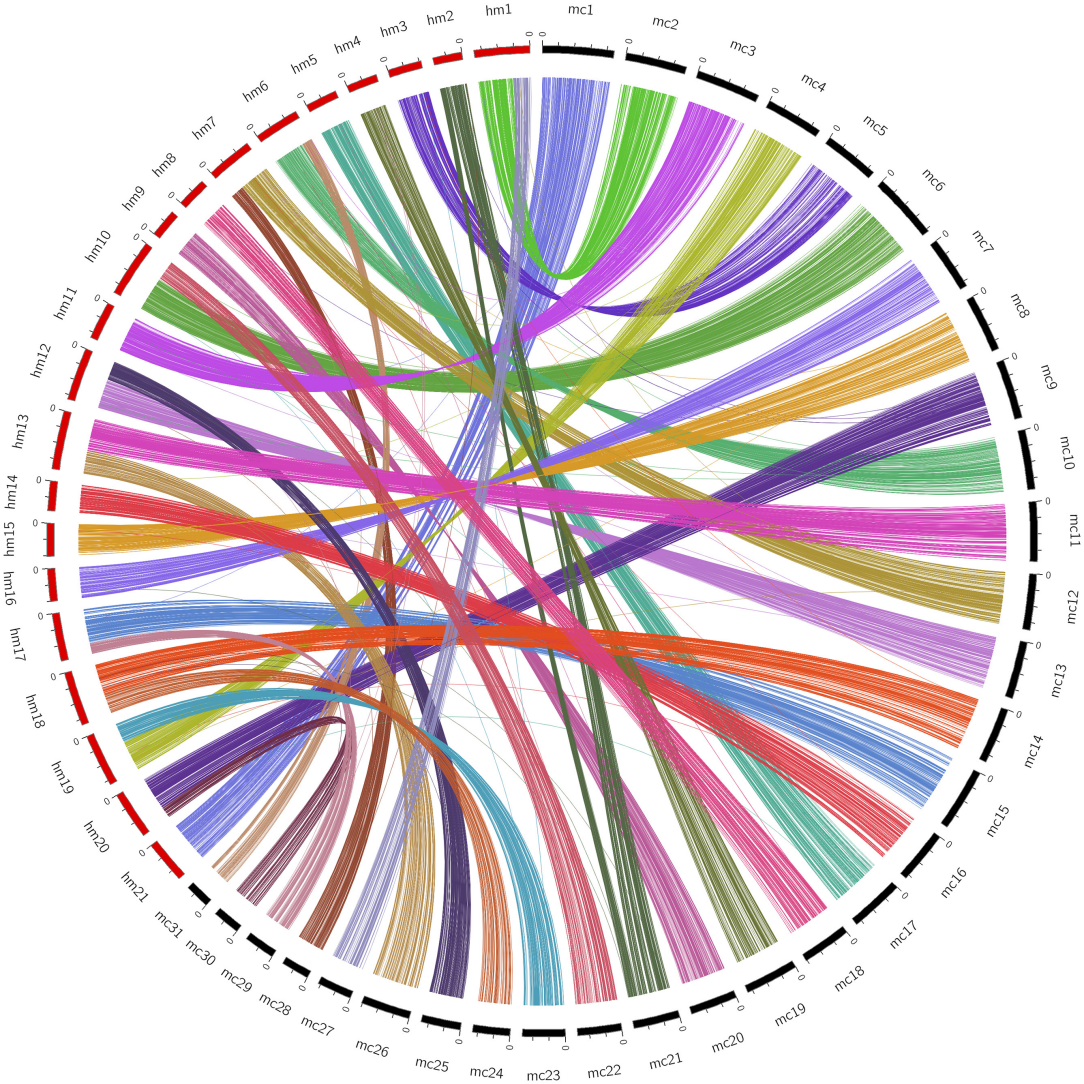
A circos plot showing the orthologs between *M. cinxia* and *H. melpomene*. Orthologs between *M. cinxia* and *H. melpomene* were identified using OrthoFinder and filtered for 1-to-1 orthologs. The internal links in the circos plot indicate the orthologs between *M. cinxia* and *H. melpomene*. The links are coloured according to the *M. cinxia* chromosome.

Predicted protein sequences from *Bombyx mori* [69] (January 2017 gene models), *P. napi* [46], and *H. melpomene* (Hmel2.5) [44, 45] were downloaded from SilkBase [70], LepBase [31], and the Butterfly Genome Database [71]. OrthoFinder v2.3.3 (OrthoFinder, RRID:SCR_017118) [72] was run to identify orthologs between *M. cinxia, B. mori, P. napi*, and *H. melpomene* using blast as the search tool (Fig. 3 and Supplementary Fig. S14).

## Data Validation and Quality Control

**Figure 4:**
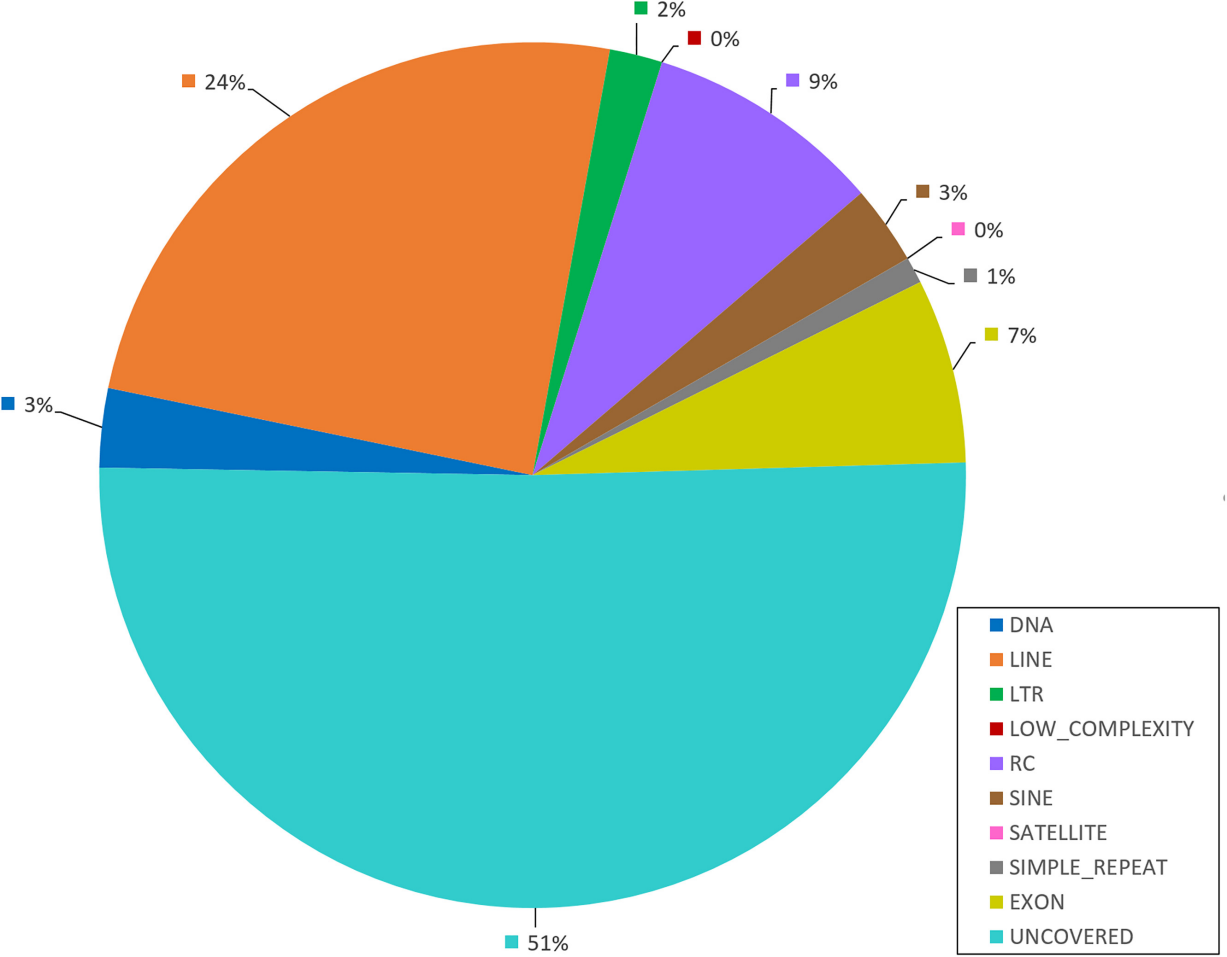
Relative amounts of different repeat classes in *M. cinxia* genome. Repeat classes and coverage of the *M. cinxia* genome v2: DNA: Class II; LINE: long interspersed nuclear elements; LTR: long terminal repeats; LOW_COMPLEXITY: low-complexity repeated DNA; RC: rolling circle elements (e.g., helitrons); SINE: short interspersed nuclear elements; Satellite: satellite DNA; SIMPLE_REPEAT: simple repeated motifs; EXON: exonic regions; UNCOVERED: rest of the chromosomes.

To assess the quality of the assembly, assembly statistics were generated using assembly-stats [30] and compared to the v1 genome, as well as the *H. melpomene, B. mori*, and *P. napi* genome assemblies (Table 1). The new genome contains 94 Mb more sequence than the previous scaffold assembly. On the basis of the observations of individual alignments in the full genome alignment between Version 1 and Version 2, there are many regions in Genome 1 that are aligned into multiple positions in Version 2. This points to collapsed repeat regions in Version 1 and more accurate repeat placement due to the long-read sequencing in Version 2. The N50 length and L50 value at scaffold or chromosome level improved greatly compared to the previous genome. To check for possible duplication or missing areas in the assembly, an assessment was made for the completeness of single-copy orthologs from BUSCO [64, 65] eukaryota, arthropoda, and metazoa gene sets (Table 3). In each of the gene sets, 93.0–94.9% of the expected single-copy orthologs were found in complete copies. The duplication rate was estimated to be between 1.4 and 1.5%. A total of 1,232 gene models were manually curated using the Apollo annotation system [66] to ensure the quality of the models. To test for contamination, the predicted protein sequences were checked with AAI-profiler [73] to identify sequences originating from different taxa (Supplementary Files S1–S3). Overall, 42% of the genome was composed of repeat sequences (Fig. 4 and Supplementary Figs S15–S20 [chromosome-specific repeat classes]). There were no clear differences in the repeat contents between chromosomes (Supplementary Table S1), which further supports the more accurate placement of repeats due to the long-read sequencing in Version 2. Long interspersed nuclear elements (LINEs) were the most prevalent.

**Table 3:** BUSCO completeness estimates of the v2 genome based on the eukaryota, arthropoda, and metazoa gene sets

Lineage	BUSCO Category, No. (%)				
	Complete	Single-copy	Duplicated	Fragmented	Missing
Eukaryota	237 (93.0)	234 (91.8)	3 (1.2)	9 (3.5)	9 (3.5)
Arthropoda	960 (94.8)	946 (93.4)	14 (1.4)	16 (1.6)	37 (3.6)
Metazoa	905 (94.9)	891 (93.4)	14 (1.5)	16 (1.7)	33 (3.4)

## Reuse potential

The substantial improvements in contiguity and gene annotation quality of the new genome will enable a range of important new studies and open up possibilities for future work. The results also demonstrate that with the use of proper computational tools and data, it is possible to obtain a high-quality, chromosome-scale reference genome even when a single individual organism will not provide enough high molecular weight DNA for long-read sequencing. Furthermore, we show the potential of the linkage mapping: it anchors contigs to actual chromosomes instead of just linking different contigs together as is done, for example, in the Hi-C approach. Moreover, the haplotype problem is not tackled by Hi-C. Our high-density linkage map allows us to put nearly all contigs into chromosomes. It is worth noting that the linkage map is not scaffolding directly but it puts contigs into map positions; scaffolding is possible if a contig spans 2 or more map positions. Otherwise, the contig can be placed only partially. In addition to the linkage map approach, we used extensive manual curation of the assembly to avoid chimeric parts and improve the assembly quality. Current research aims at identifying mechanisms underlying key life history adaptations, exploring the extent of natural variation and selection on these adaptations in wild populations, and integrating these insights with the exceptional ecological, demographic, and climatic data available for this system. Future studies in this direction will help identify the mechanisms maintaining variation in life histories across spatial and temporal scales, and the extent to which phenotypic variation in these and other traits may contribute to a population's adaptive capacity under climate change. Several studies in different species illustrate how stress responses can be crucial for survival under variable environments, both within and between generations. The Glanville fritillary is being used to explore how environmental information is translated into adaptive phenotypic changes, and how these responses are transmitted to future generations, using transcriptomic and epigenetic approaches. Such studies will benefit from an improved annotation permitting exon-specific expression quantification, and identification of epigenetic marks and other functional variants outside coding regions. Exploiting current and past large-scale sampling efforts, these new studies apply population genomic approaches that are facilitated by the increased assembly contiguity, e.g., by permitting linkage disequilibrium and haplotype-based selection analyses. Other avenues of research enabled by the improved genome assembly include structural variation, regulatory evolution, recombination rate variation, and coalescent-based demographic analyses. The increasing availability of chromosome-level lepidopteran genomes such as ours permits exciting new comparative phylogenetic analyses, e.g., of chromosome and genome evolution.

## Data Availability

The SMRT sequencing reads used for the genome assembly are available in the NCBI SRA and can be accessed with Bioproject PRJNA607899 accession No. SRR11184190.

The genome has been deposited to GenBank under Bioproject PRJNA607899.

The Illumina reads used for the linkage map are available in the NCBI SRA and can be accessed with Bioproject PRJNA608928 accession Nos. SRR11186917–SRR11187107.

Transcriptome 1 RNA-seq reads are available in NCBI GEO and can be accessed with accession No. GSE159376.

Transcriptome 2 RNA-seq reads are available in NCBI SRA and can be accessed with Bioproject PRJNA670126.

All supporting data and materials are available in the *GigaScience* GigaDB database [74].

## Additional Files

**Supplementary Figure S1:**  *M. cinxia* aligned against *H. melpomene* using the last aligner [47]. A: *M. cinxia* chromosome 1 (M01_B01_H21), B: chromosome 2 (M02_B04_H01a), C: chromosome 3 (M03_B15_H11), D: chromosome 4 (M04_B12_H19a), E: chromosome 5 (M05_B06_H03), and F: chromosome 6 (M06_B05_H10a).

**Supplementary Figure S2:**  *M. cinxia* aligned against *H. melpomene* using the last aligner [47]. A: *M. cinxia* chromosome 7 (M07_B18_H16), B: chromosome 8 (M08_B17_H15), C: chromosome 9 (M09_B10_H20a), D: chromosome 10 (M10_B09_H06a), E: chromosome 11 (M11_B22_H13a), and F: chromosome 12 (M12_B11a_H07a).

**Supplementary Figure S3:**  *M. cinxia* aligned against *H. melpomene* using the last aligner [47]. A: *M. cinxia* chromosome 13 (M13_B08_H12a), B: chromosome 14 (M14_B23a_H18a), C: chromosome 15 (M15_B13_H17a), D: chromosome 16 (M16_B19_H14), E: chromosome 17 (M17_B03_H05), and F: chromosome 18 (M18_B25_H08).

**Supplementary Figure S4:**  *M. cinxia* aligned against *H. melpomene* using the last aligner [47]. A: *M. cinxia* chromosome 19 (M19_B21_H04), B: chromosome 20 (M20_B07_H09), C: chromosome 21 (M21_B16_H02), D: chromosome 22 (M22_B28_H10b), E: chromosome 23 (M23_B26_H19b), and F: chromosome 24 (M24_B27_H18b).

**Supplementary Figure S5:**  *M. cinxia* aligned against *H. melpomene* using the last aligner [47]. A: *M. cinxia* chromosome 25 (M25_B20_H12b), B: chromosome 26 (M26_B14_H13b), C: chromosome 27 (M27_B24a_H01b), D: chromosome 28 (M28_B02_H07b), E: chromosome 29 (M29_B24b_H17b), and F: chromosome 30 (M30_B23b_H20b).

**Supplementary Figure S6:**  *M. cinxia* aligned against *H. melpomene* using the last aligner [47]. *M. cinxia* chromosome 31 (M31_B11b_H06b).

**Supplementary Figure S7:** A dot-plot showing the structure of *P. napi* genome against *M. cinxia* genome v.2. The diagonal lines indicate the collinearity between the 2 species.

**Supplementary Figure S8:**  *M. cinxia* aligned against *P. napi* using the last aligner [47]. A: *M. cinxia* chromosome 1 (M01_B01_H21), B: chromosome 2 (M02_B04_H01a), C: chromosome 3 (M03_B15_H11), D: chromosome 4 (M04_B12_H19a), E: chromosome 5 (M05_B06_H03), and F: chromosome 6 (M06_B05_H10a).

**Supplementary Figure S9:**  *M. cinxia* aligned against *P. napi* using the last aligner [47]. A: *M. cinxia* chromosome 7 (M07_B18_H16), B: chromosome 8 (M08_B17_H15), C: chromosome 9 (M09_B10_H20a), D: chromosome 10 (M10_B09_H06a), E: chromosome 11 (M11_B22_H13a), and F: chromosome 12 (M12_B11a_H07a).

**Supplementary Figure S10:**  *M. cinxia* aligned against *P. napi* using the last aligner [47]. A: *M. cinxia* chromosome 13 (M13_B08_H12a), B: chromosome 14 (M14_B23a_H18a), C: chromosome 15 (M15_B13_H17a), D: chromosome 16 (M16_B19_H14), E: chromosome 17 (M17_B03_H05), and F: chromosome 18 (M18_B25_H08).

**Supplementary Figure S11:**  *M. cinxia* aligned against *P. napi* using the last aligner [47]. A: *M. cinxia* chromosome 19 (M19_B21_H04), B: chromosome 20 (M20_B07_H09), C: chromosome 21 (M21_B16_H02), D: chromosome 22 (M22_B28_H10b), E: chromosome 23 (M23_B26_H19b), and F: chromosome 24 (M24_B27_H18b).

**Supplementary Figure S12:**  *M. cinxia* aligned against *P. napi* using the last aligner [47]. A: *M. cinxia* chromosome 25 (M25_B20_H12b), B: chromosome 26 (M26_B14_H13b), C: chromosome 27 (M27_B24a_H01b), D: chromosome 28 (M28_B02_H07b), E: chromosome 29 (M29_B24b_H17b), and F: chromosome 30 (M30_B23b_H20b).

**Supplementary Figure S13:**  *M. cinxia* aligned against *P. napi* using the last aligner [47]. *M. cinxia* chromosome 31 (M31_B11b_H06b).

**Supplementary Figure S14:** Orthologs between *M. cinxia* and *P. napi* were identified using OrthoFinder and filtered for 1-to-1 orthologs. The internal links in the circos plot indicate the orthologs between *M. cinxia* and *P. napi*.  The links are coloured according to the *M. cinxia* chromosome.

**Supplementary Figure S15:** Repeat classes and coverage of the *M. cinxia* genome v.2. A: M. cinxia chromosome 1 (M01_B01_H21), B: chromosome 2 (M02_B04_H01a), C: chromosome 3 (M03_B15_H11), D: chromosome 4 (M04_B12_H19a), E: chromosome 5 (M05_B06_H03), and F: chromosome 6 (M06_B05_H10a). (DNA: Class II; LINE: long interspersed elements; LTR: long terminal repeats; Low_complexity: low-complexity repeated DNA; RC: rolling circle elements [e.g., Helitrons]; SINE: short interspersed nuclear elements; Satellite: satellite DNA; Simple_repeat: simple repeated motifs; Exon: exonic regions; Uncovered: rest of the chromosome).

**Supplementary Figure S16:** Repeat classes and coverage of the *M. cinxia* genome v.2. A: *M. cinxia* chromosome 7 (M07_B18_H16), B: chromosome 8 (M08_B17_H15), C: chromosome 9 (M09_B10_H20a), D: chromosome 10 (M10_B09_H06a), E: chromosome 11 (M11_B22_H13a), and F: chromosome 12 (M12_B11a_H07a). (DNA: Class II; LINE: long interspersed elements; LTR: long terminal repeats; Low_complexity: low-complexity repeated DNA; RC: rolling circle elements [e.g., Helitrons]; SINE: short interspersed nuclear elements; Satellite: satellite DNA; Simple_repeat: simple repeated motifs; Exon: exonic regions; Uncovered: rest of the chromosome).

**Supplementary Figure S17:** Repeat classes and coverage of the *M. cinxia* genome v.2. A: *M. cinxia* chromosome 13 (M13_B08_H12a), B: chromosome 14 (M14_B23a_H18a), C: chromosome 15 (M15_B13_H17a), D: chromosome 16 (M16_B19_H14), E: chromosome 17 (M17_B03_H05), and F: chromosome 18 (M18_B25_H08). (DNA: Class II; LINE: long interspersed elements; LTR: long terminal repeats; Low_complexity: low-complexity repeated DNA; RC: rolling circle elements [e.g., Helitrons]; SINE: short interspersed nuclear elements; Satellite: satellite DNA; Simple_repeat: simple repeated motifs; Exon: exonic regions; Uncovered: rest of the chromosome).

**Supplementary Figure S18:** Repeat classes and coverage of the *M. cinxia* genome v.2. A: *M. cinxia* chromosome 19 (M19_B21_H04), B: chromosome 20 (M20_B07_H09), C: chromosome 21 (M21_B16_H02), D: chromosome 22 (M22_B28_H10b), E: chromosome 23 (M23_B26_H19b), and F: chromosome 24 (M24_B27_H18b). (DNA: Class II; LINE: long interspersed elements; LTR: long terminal repeats; Low_complexity: low-complexity repeated DNA; RC: rolling circle elements [e.g., Helitrons]; SINE: short interspersed nuclear elements; Satellite: satellite DNA; Simple_repeat: simple repeated motifs; Exon: exonic regions; Uncovered: rest of the chromosome).

**Supplementary Figure S19:** Repeat classes and coverage of the *M. cinxia* genome v.2. A: *M. cinxia* chromosome 25 (M25_B20_H12b), B: chromosome 26 (M26_B14_H13b), C: chromosome 27 (M27_B24a_H01b), D: chromosome 28 (M28_B02_H07b), E: chromosome 29 (M29_B24b_H17b), and F: chromosome 30 (M30_B23b_H20b). (DNA: Class II; LINE: long interspersed elements; LTR: long terminal repeats; Low_complexity: low-complexity repeated DNA; RC: rolling circle elements [e.g., Helitrons]; SINE: short interspersed nuclear elements; Satellite: satellite DNA; Simple_repeat: simple repeated motifs; Exon: exonic regions; Uncovered: rest of the chromosome).

**Supplementary Figure S20:** Repeat classes and coverage of the *M. cinxia* genome v.2. *M. cinxia* chromosome 31 (M31_B11b_H06b). (DNA: Class II; LINE: long interspersed elements; LTR: long terminal repeats; Low_complexity: low-complexity repeated DNA; RC: rolling circle elements [e.g., Helitrons]; SINE: short interspersed nuclear elements; Satellite: satellite DNA; Simple_repeat: simple repeated motifs; Exon: exonic regions; Uncovered: rest of the chromosome).

**Supplementary File S1:**Report from AAI-profiler on predicted protein sequences.

**Supplementary File S2:**Results from AAI-profiler on matrix-format on predicted protein sequences.

**Supplementary File S3:**Results from AAI-profiler on krona plot format on predicted protein sequences.

**Supplementary File S4:**List of prioritized gene families selected based on particular interest in butterfly research

**Supplementary File S5:**Kmer analysis for genome size estimation

**Supplementary Table S1:** Repeat contents of chromosomes

## Abbreviations

BLAST: Basic Local Alignment Search Tool; bp: base pairs; BUSCO: Benchmarking Universal Single-Copy Orthologs; BWA: Burrows-Wheeler Aligner; CI: confidence interval; Gb: gigabase pairs; LINE: long interspersed nuclear element; LTR: long terminal repeat; Mb: megabase pair; mRNA: messenger RNA; NCBI: National Center for Biotechnology Information; PacBio: Pacific Biosciences; PE: paired-end; RAD sequencing: restriction-site associated DNA sequencing; RAM: random access memory; SMRT: Single Molecule, Real-Time; SRA: Sequence Read Archive.

## Ethics Approval and Consent to Participate

There are no ethical policies related to working with insect data. The Glanville fritillary is not considered endangered in the Åland Islands and no permits are required for sampling. However, we note that within this project the larval sampling for genetic analyses is done non-invasively in the field, ensuring insignificant demographic impact. In addition, because the sampling takes place prior to diapause (Åland) when mortality is generally the highest, the collection has negligible effect on family survival or the demographic characteristics of populations.

## Competing Interests

The authors declare that they have no competing interests.

## Funding

Funding for M.S., D.B., V.O., E.v.B., J.T., and A.K. was provided by a grant from the European Research Council (Independent Starting Grant No. 637,412 "META-STRESS" to M.S.) and J.Ö.-U., V.A., and D.B. from the Academy of Finland grant (Decision No. 304,041 to M.S. and Decision No. 283,108 to Ilkka Hanski). A.D. was funded by a Marie Sklodowska Curie Individual Fellowship (No. 790,531, Host Sweet Home). O.-P.S. was supported by the “TTÜ development program 2016–2022,” project code 2014–2020.4.01.16–0032.

## Authors' Contributions

O.-P.S. assembled the genome, processed the chimeric contigs, performed the gap filling and the polishing of the assembly, and participated in the genome analysis.

V.A. was responsible for the initial idea of the approach for the genome-related activities, coordinated the first part of the project, designed and produced data for the linkage map, and worked on solving the haplotypes from the initial assembly.

D.B. performed gene prediction, functional annotation, ortholog prediction, and managed the manual annotation.

J.I.K. installed and managed the Apollo annotation server.

S.I. was responsible for larval rearing and preparation of butterfly crosses.

P.R. performed the linkage mapping and anchored the genome onto chromosomes.

V.O. assembled the transcriptomes used for gene prediction.

L. Pellegrini manually inspected the chimeric contigs.

A.R. performed DNA extraction.

D.B., J.I.K., V.O., T.D., M.F.D., A.D., I.C.D., P.H., A.K., S.S.K., S.O.K., E.L., S.L., J.M., A.N., M.C.-M., V.P., T.S., A.I.T., V.T., E.v.B., J.Ö.-U., and M.S. participated in manual annotation.

J.T. performed the annotation of transposable elements and repeat classes.

L. Paulin was responsible for the management of the DNA sequencing.

M.J.F. was responsible for the management of the genome analysis.

P.A. was responsible for the initial idea of the approach for the genome-related activities, and the management of the genome analysis.

M.S. was responsible for the management of the *M. cinxia* database and genome analysis.

O.-P.S., D.B., V.A., P.R., J.T., J.I.K., V.O., L.P., M.J.F., P.A., and M.S. wrote the manuscript.

## Supplementary Material

giab097_GIGA-D-20-00318_Original_Submission

giab097_GIGA-D-20-00318_Revision_1

giab097_GIGA-D-20-00318_Revision_2

giab097_GIGA-D-20-00318_Revision_3

giab097_Response_to_Reviewer_Comments_Revision_2

giab097_Reviewer_1_Report_Original_Submission Annabel Charlotte Whibley -- 1/12/2021 Reviewed

giab097_Reviewer_2_Report_Original_Submission Shanlin Liu -- 1/26/2021 Reviewed

giab097_Reviewer_2_Report_Revision_1 Shanlin Liu -- 6/4/2021 Reviewed

giab097_Reviewer_2_Report_Revision_2 Shanlin Liu -- 7/1/2021 Reviewed

giab097_Supplemental_Files

## Notes

Corresponding authors contributed equally.
