# Supplementary material for: Improved chromosome-level genome assembly of the Glanville fritillary butterfly (Melitaea cinxia) integrating Pacific Biosciences long reads and a high-density linkage map
Source: Gigascience. 2022 Jan 12;11:giab097. doi: 10.1093/gigascience/giab097 (PMC8756199; doi:10.1093/gigascience/giab097)
Supplement: giab097_GIGA-D-20-00318_Original_Submission [file giab097_giga-d-20-00318_original_submission.pdf]

GigaScience

Improved chromosome level genome assembly of the Glanville fritillary butterfly (Melitaea cinxia) based on SMRT Sequencing and linkage map.

--Manuscript Draft--

**Manuscript Number:**	GIGA-D-20-00318
**Full Title:**	Improved chromosome level genome assembly of the Glanville fritillary butterfly (Melitaea cinxia) based on SMRT Sequencing and linkage map.
**Article Type:**	Data Note
**Funding Information:**	H2020 European Research Council (637412) — Dr Marjo Saastamoinen<br>Academy of Finland (304041) — Dr Marjo Saastamoinen<br>Academy of Finland (283108) — Not applicable<br>H2020 Marie Skłodowska-Curie Actions (790531) — Ms Anne Duploy<br>Sihtasutus Archimedes (2014-2020.4.01.16-0032) — Not applicable
**Abstract:**	The Glanville fritillary (Melitaea cinxia) butterfly is a long-term model system for metapopulation dynamics research in fragmented landscapes. Here, we provide a chromosome level assembly of the butterfly's genome produced from Pacific Biosciences sequencing of a pool of males, combined with a linkage map from population crosses. The final assembly size of 484 Mb is an increase of 94 Mb on the previously published genome. Estimation of the completeness of the genome with BUSCO, indicates that the genome contains 93 - 95% of the BUSCO genes in complete and single copies. We predicted 14,830 gene models using the MAKER pipeline and manually curated 1,232 of these gene models. The genome and its annotated gene models are a valuable resource for future comparative genomics, molecular biology, transcriptome and genetics studies on this species.
**Corresponding Author:**	Olli-Pekka Smolander<br>Tallinn University of Technology<br>Tallinn, Harju ESTONIA
**Corresponding Author Secondary Information:**	
**Corresponding Author's Institution:**	Tallinn University of Technology
**Corresponding Author's Secondary Institution:**	
**First Author:**	Daniel Blande
**First Author Secondary Information:**	
**Order of Authors:**	Daniel Blande
	Olli-Pekka Smolander
	Virpi Ahola
	Pasi Rastas
	Jaakko Tanskanen
	Juhana I Kammonen
	Vincencio Oostra
	Lorenzo Pellegrini
	Suvi Ikonen
	Tad Dallas

Powered by Editorial Manager® and ProduXion Manager® from Aries Systems Corporation

	Michelle F DiLeo
	Anne Duploy
	Ilhan Cem Duru
	Pauliina Halimaa
	Aapo Kahilainen
	Suyog S Kuwar
	Sirpa O Kärenlampi
	Elvira Lafuente
	Shiqi Luo
	Jenny Makkonen
	Abhilash Nair
	Maria de la Paz Celorio-Mancera
	Ville Pennanen
	Annukka Ruokolainen
	Tarja Sundell
	Arja I Tervahauta
	Victoria Twort
	Erik van Bergen
	Janina Österman-Udd
	Lars Paulin
	Mikko J Frilander
	Petri Auvinen
	Marjo Saastamoinen
**Order of Authors Secondary Information:**	
**Additional Information:**	
**Question**	**Response**
Are you submitting this manuscript to a special series or article collection?	No
**Experimental design and statistics**<br><br>Full details of the experimental design and statistical methods used should be given in the Methods section, as detailed in our Minimum Standards Reporting Checklist. Information essential to interpreting the data presented should be made available in the figure legends.<br><br>Have you included all the information requested in your manuscript?	Yes

Powered by Editorial Manager® and ProduXion Manager® from Aries Systems Corporation

**Resources**<br><br>A description of all resources used, including antibodies, cell lines, animals and software tools, with enough information to allow them to be uniquely identified, should be included in the Methods section. Authors are strongly encouraged to cite Research Resource Identifiers (RRIDs) for antibodies, model organisms and tools, where possible.<br><br>Have you included the information requested as detailed in our Minimum Standards Reporting Checklist?	Yes
**Availability of data and materials**<br><br>All datasets and code on which the conclusions of the paper rely must be either included in your submission or deposited in publicly available repositories (where available and ethically appropriate), referencing such data using a unique identifier in the references and in the "Availability of Data and Materials" section of your manuscript.<br><br>Have you have met the above requirement as detailed in our Minimum Standards Reporting Checklist?	Yes

Powered by Editorial Manager® and ProduXion Manager® from Aries Systems Corporation

Manuscript Click here to access/download;Manuscript;Cinxia_genome_manuscript_giga

Title page

Improved chromosome level genome assembly of the Glanville fritillary butterfly (*Melitaea cinxia*) based on SMRT Sequencing and linkage map.

Daniel Blande[*,1], Olli-Pekka Smolander*[2,3], Virpi Ahola[1,4], Pasi Rastas[2], Jaakko Tanskanen[5], Juhana I. Kammonen[2], Vicencio Oostra[1,6], Lorenzo Pellegrini[2], Suvi Ikonen[1], Tad Dallas[7], Michelle F. DiLeo[1], Anne Duplouy[1,8], Ilhan Cem Duru[2], Pauliina Halimaa[9], Aapo Kahilainen[1], Suyog S. Kuwar[10,11], Sirpa O. Kärenlampi[9], Elvira Lafuente[12], Shiqi Luo[13], Jenny Makkonen[9], Abhilash Nair[1], Maria de la Paz Celorio-Mancera[14], Ville Pennanen[15], Annukka Ruokolainen[1], Tarja Sundell[2], Arja I. Tervahauta[9], Victoria Twort[8], Erik van Bergen[1], Janina Österman-Udd[1], Lars Paulin[2], Mikko J. Frilander[#,2], Petri Auvinen[#, 2], Marjo Saastamoinen[#1,16]

* These authors contributed equally to the work

# These authors contributed equally to the work

Abstract

The Glanville fritillary (*Melitaea cinxia*) butterfly is a long-term model system for metapopulation dynamics research in fragmented landscapes. Here, we provide a chromosome level assembly of the butterfly's genome produced from Pacific Biosciences sequencing of a pool of males, combined with a linkage map from population crosses. The final assembly size of 484 Mb is an increase of 94 Mb on the previously published genome. Estimation of the completeness of the genome with BUSCO, indicates that the genome contains 93 - 95% of the BUSCO genes in complete and single copies. We predicted 14,830 gene models using the MAKER pipeline and manually curated 1,232 of these gene models.

The genome and its annotated gene models are a valuable resource for future comparative genomics, molecular biology, transcriptome and genetics studies on this species.

Keywords

Melitaea cinxia, Glanville fritillary, Genome, Spatial Ecology

Data Description

Context

Identifying and characterizing genes underlying ecologically and evolutionarily relevant phenotypes in natural populations has become possible with novel genomic tools that can also be utilized in 'non-model' organisms. The Glanville fritillary (*Melitaea cinxia*) butterfly, and in particular its metapopulation in the Åland Islands (SW Finland), is an ecological model system in spatial ecology[1,2]. In short, within this archipelago, the species inhabits a network of dry outcrop meadows and pastures, and persists as a classic metapopulation with high turnover in patch occupancy[1]. The network of 4,500 potential habitat patches has been systematically surveyed bi-annually for butterfly occupancy and abundance since 1993[3], providing a vast amount of ecological data on population dynamics[2]. Experimental manipulations under more controlled conditions are also possible due to the small size, high fecundity and relatively short generation time of the species. Consequently, the ecological understanding of the species expands to the life history ecology across development stages[4] (e.g. Kahilainen et al. unpubl.), dispersal dynamics[5,6], species interactions (host plants and parasitoids)[7-11], and stress tolerance[12,13]. During the last decade, the system has also been used to study genetic and evolutionary processes, such as identifying candidate genes

underlying variation and evolution of dispersal in fragmented habitats[14] and host plant preference[15], and assessing allelic variation and their dynamics in space and time with SNP data across populations[16-18]. Several approaches have been used to explore the genetic underpinnings of phenotypic variation in the Glanville fritillary metapopulation, ranging from candidate gene approaches[12,19] to quantitative genetics[20,21], and whole-genome scans[22,23] (including transcriptomics; Kahilainen et al. unpubl.), and included both laboratory and natural environmental conditions.

The first *M. cinxia* genome was released in 2014[24]. This genome was produced from a combination of 454 sequencing for contig assembly, followed by scaffolding with Illumina paired-end (PE), SOLiD mate-pair reads and PacBio data. The size of the final assembly was 390 Mb made up from 8,261 scaffolds, with a scaffold N50 of 119,328. Scaffolds were assigned to chromosomes based on a linkage map produced from RAD sequencing[25]. It was considered that a new genome, sequenced using longer PacBio reads, would result in a more complete assembly and better represent the repetitive areas of the genome.

Here, a new sequencing and assembly of the *M. cinxia* genome has been carried out using a pool of seven male butterflies from a single larval family collected from Sottunga, an island in an eastern part of the archipelago. Sequencing was conducted using the PacBio RSII sequencer. An initial assembly was created using FALCON[26,27] followed by polishing performed with Quiver[26]. A new linkage map was created and used to assign the assembled scaffolds to their correct positions and orientations within the 31 chromosomes. The scaffolds were then gap-filled producing a final assembly of 484 Mb with a scaffold N50 of 17,331,753 bp. Gene prediction on the genome assembly was carried out using MAKER v 2.31.10[28] that was run iteratively using several independent training sets. Manual annotation was performed for 1,232 of the gene models. The genome assembly increases greatly in contiguity and completeness compared to the first genome (Table 1) with superscaffold N50

values of 17,331,753 bp in the new genome compared to 9,578,794 bp in the version 1 genome.

Table 1. Assembly statistics were calculated for the *M. cinxia* v2 genome, *M. cinxia* v1 scaffolds, *M. cinxia* v1 chromosomes and *B. mori* using the assembly-stats program (https://zenodo.org/badge/latestdoi/20772/rjchallis/assembly-stats). Statistics for *H. melpomene* v2.5 and *P. napi* v1.1 were obtained from LepBase[64].

	***M. cinxia* Version 2**	***M. cinxia* Version 1 Scaffolds**	***M. cinxia* Version 1 Chromo-somes**	***Heliconius melpomene* Hmel2.5**	***Bombyx mori***	***Pieris napi* v1.1**
Length (bp)	484,462,241	389,907,520	250,926,848	275,245,813	460,334,017	349,759,982
N(%)	0.00	7.42	7.84	0.38	0.10	22.47
GC(%)	33.83	32.56	32.38	32.79	38.54	33.32
AT(%)	66.17	67.44	67.62	67.21	61.46	66.68
Scaffold count	31	8,261	31	332	696	2,969
Longest scaffold (bp)	22,190,643	668,473	13,295,773	18,127,314	21,465,692	15,427,984
Scaffold N50 length (bp)	17,331,753	119,328	9,578,794	14,308,859	16,796.068	12,597,868
Scaffold N50 count (L50)	13	970	12	9	13	13
Contig Count	529	48,180	26,662	3,126	726	53,510
Contig N50 length (bp)	1,831,849	14,057	15,707	328,886	12,201,325	10,538
Contig N50 count (L50)	79	7,366	4,286	214	16	6,914

Methods

An overview of the processing pipeline for the work is shown in Figure 1.

Genomic samples and DNA extraction

Owing to the facultatively univoltine life cycle of the butterfly in Finland, experimental inbreeding of the species would have taken several years. Therefore, we chose to sample individuals from an island population, Sottunga, expected to harbour low genetic diversity. Sottunga is part of the Åland Islands archipelago in the northern Baltic Sea, and the population was introduced here in 1991 using individuals collected on the mainland of Åland Island[29]. This introduction was carried out with 71 larval families. The distance to the nearest *M. cinxia* population across the water is 5 km, and we therefore assume that the introduced population has been (almost) completely isolated ever since. Furthermore, the effective population size of *M. cinxia* in Sottunga has been very low during the last 24 years (on average 57 larval nests/year in 1993-2019), and it has experienced several strong bottlenecks[30]. Using genomic markers, Fountain et al.[16] demonstrated that samples from the Sottunga population separate clearly from samples collected on the mainland.

During the fall survey of 2014 (see Ojanen et al. for details of the survey[3]) we collected individuals from one larval group on the island of Sottunga (patch number 1439, see Schulz et al. for specific location[31]). The larvae were collected once they were in diapause and most likely comprise full-sibs[17]. The larval group was kept in diapause (+5 °C) until the following spring and then reared to adulthood under common garden conditions (28:8°C; 12L:12D) at the Lammi Biological Station, University of Helsinki. After eclosion, butterflies were sexed and stored at -80°C. High-molecular-weight DNA was isolated from seven adult males using the caesium chloride (CsCl) method[24].

SMRT sequencing libraries and sequencing

Library construction for Pacific Biosciences sequencing was carried out using the protocols recommended by the manufacturer (Pacific Biosciences, Menlo Park, CA, USA). Genomic

DNA was sheared using a Megaruptor (Diagenode, Seraing, Belgium) followed by damage repair, end-repair, hairpin ligation, and size selection using BluePippin (Sage Science, Beverly, MA, USA). After primer annealing and polymerase binding, the DNA templates were sequenced on a PacBio RSII sequencer using P6/C4 chemistry and 360 min video time at the DNA Sequencing and Genomics Laboratory, Institute of Biotechnology, University of Helsinki, Finland[32].

Genome Assembly

The genome was assembled using the FALCON assembler (FALCON-Integrate-1.8.6)[26,27] with a read cut-off of 18,000. This cut-off was experimentally found to give the best contiguity for the assembly, while minimizing (within a small margin of error) the percentage of possibly erroneous contigs. The assembly errors were evaluated based on a genetic map from the previously published genome[24]. The assembly was based on 1,939,889 PacBio reads, 24,409,505,551 bp in total, with an N50 of 18,479 bp which is approximately 50x coverage based on the final genome size. With the selected read cut-off the data produced 10.8 Gb of corrected reads which were further assembled using the Falcon software. The assembly yielded 4,559 primary contigs containing 739.9 Mb with an N50 of 340 kb and 1,661 alternative contigs containing 118.1 Mb with an N50 of 85,246 bp. The data were also assembled using miniasm software (0.2-r137-dirty)[33] which yielded similar results.

To evaluate the putative chimeric contigs and assembly errors suggested by the genetic map, the raw SMRT sequencing data were mapped to the assembly using the Burrows-Wheeler Aligner (BWA-0.7.17) with the MEM algorithm[34]. The alignments of the 425 regions discovered as possibly chimeric were visually inspected. Of these regions, 92 showed even read coverage and no evident signs of assembly errors, while 333 regions contained areas

with low coverage and/or repeat regions that led to erroneous overlaps and misassemblies. The assembly was split in the positions where the error was observed. The resulting assembly was polished using the SMRT sequencing data and Quiver[26] software from the SMRT Tools-package (PacBio).

Linkage Map

Linkage mapping was constructed from whole genome resequencing data of F2 crosses of *M. cinxia* (Ahola et al., unpublished). The grandparents of these F2 crosses are offspring of wild collected *M. cinxia* originating from two distantly related *M. cinxia* populations around the Baltic Sea; the Åland Islands (ÅL)[1] and Pieni Tytärsaari (PT) populations[35]. Between population crosses of type ÅL♂xPT♀ and ÅL♀xPT♂were established to create the F1 population. Part of these F1 individuals were used to establish the F2 families, actively avoiding mating among siblings. A subset of the resulting full-sib families were reared to adulthood, and five of these F2 families, together with their parents and grandparents, were selected for resequencing. In total, resequencing included ten grandparental individuals, ten F1 parents and 165 F2 individuals (N=185).

All the larvae from different generations completed development under common garden conditions (28:15°C; 12L:12D) utilizing fresh leaves of greenhouse grown *Veronica spicata*. Diapausing larvae were kept in a growth chamber at +5°C and 80% RH for approximately seven months to mimic the normal wintertime conditions for these butterflies. Adults were kept in hanging cages (of 50 cm height and 40 cm diameter) at ~26:18°C; 9L:15, and fed *ad libitum* with 20% honey-water solution throughout the experiments.

Before DNA extraction the adult butterflies were stored at -80°C, and either thorax or abdomen tissue of these individuals was used for sequencing. Tissues were homogenized prior to extraction using TissueLyser (Qiagen, Venlo, The Netherlands) at 30/s for 1.5 mins

with Tungsten Carbide Beads, 3 mm (Qiagen, Venlo, The Netherlands) and ATL buffer (Qiagen, Venlo, The Netherlands). DNA was extracted using the NucleoSpin 96 Tissue Core Kit (Macherey-Nagel) according to the manufacturer's protocol with the exception that lysing time was extended to overnight. The samples were additionally treated with RNase A (Thermo Scientific) before sequencing. Sequencing was made using Illumina Hi-Seq with 125 bp paired-end reads.

The mapping procedure followed the Lep-MAP3[25] pipeline. First, individual fastq files were mapped to the contig assembly using BWA MEM (BWA-0.7.17) [34] and individual bam files were created using SAMtools (1.6)[36,37]. SAMtools mpileup and the scripts pileupParser2.awk and pileup2posterior.awk were used to obtain input data for Lep-MAP3. Then ParentCall2 (parameter: ZLimit=2) and Filtering2 (parameters: dataTolerance=0.0001; removeNonInformative=1; familyInformativeLimit=4) were run to obtain data with at least four informative families for each marker, resulting in a final input with almost 2.5M markers.

SeparateChromosomes2 was run on the final data (parameters lodLimit=20; samplePair=0.2;numThreads=48) to obtain 31 linkage groups with a total of 2.4M markers. OrderMarkers2 was run (parameter recombination2=0) on each linkage group (chromosome). This map was used to anchor the contig assembly into chromosomes. To validate anchoring, the map construction was repeated in the same way except that OrderMarkers2 was run on the physical order of markers to reduce noise in the linkage map. Finally, the raw data were re-mapped to the gap-filled chromosome level assembly and the linkage map was re-done in the new physical order to infer final recombination rates.

Anchoring the genome and resolving haplotypes using the linkage map

The contigs were aligned against each other and lift-over chains were created by running the first two steps (batch A1 and A2) of HaploMerger2[38]. By inspecting this chain, contigs fully contained in some longer contig were removed. Initial contig order and orientation within each chromosome was calculated by the median map position of each contig and the longest increasing subsequence of markers, respectively. For each chromosome, Marey map and contig-contig alignments from the chain were recorded. The contigs orders and orientations were manually fixed when needed and any assembly errors that were found were corrected by splitting the contigs accordingly. Also, partially haplotypic contigs were found and collapsed based on the Marey maps and contig-contig alignments. This manual work resulted in the final reference genome sequence including start and end positions of contigs in the correct order and orientation for each chromosome. Finally, the haplotype corrected genome was gap-filled using PBJelly software (PBSuite_15.8.24)[39] with the original SMRT sequencing data, and polished with the Quiver tool[26] from the SMRT Tools-package 2.3.0 (PacBio) and with Pilon (1.21)[40].

The chromosomes were aligned against the *Heliconius melpomene* (2.5)[41,42] and *Pieris napi*[43] genomes using the LAST aligner(938)[44] to check structural similarity between the species (Supplementary Figures S1-13). An overview alignment for *H. melpomene* was created using D-GENIES (1.2.0)[45] (Figure 2). The data show a high level of collinearity between *M. cinxia* and *H. melpomene* chromosomes, as described before in Ahola et al.[24]. A notably interesting point is the lack of collinearity with sex chromosomes (*M. cinxia* chromosome 1 & *H. melpomene* chromosome 21). Furthermore, the visible vertical lines show the effect of long read assembly on repeat resolution. In *M. cinxia* the repeats are placed in single chromosomes whereas in *H. melpomene* they are present in all chromosomes.

Repeat masking and annotation

Genomic assemblies were masked with *de novo* repeat libraries by RepeatMasker v.4.0.9 (http://www.repeatmasker.org/). *De novo* repeat libraries were constructed from original PacBio reads with lengths over 30,000bp and assembled scaffolds (pseudo chromosomes) using RepeatModeler v 1.0.10 (http://www.repeatmasker.org/RepeatModeler/) and the LtrHarvest/LtrDigest-pipeline[46,47]. Repeat families were clustered using cd-hit-est applying 80/80-rule (80% identity over 80% length)[48]. Repeat annotations were confirmed by RepBase Release 20181026[49] and Dfam version 3.1[50].

Transcriptome assembly

In order to aid construction of gene models, we used two transcriptome assemblies, each based on separate RNASeq data sets. Transcriptome 1 was produced using a set of 78 individually sequenced female larvae of an outbred lab population (Kahilainen et al. unpubl. data), sequenced to an average depth of 17.3M reads (read lengths 85 bp and 65 bp for forward and reverse PE reads, respectively). As the two sexes are practically indistinguishable in the larval stages, the females were identified based on homozygosity across a set of 22 Z-chromosome specific SNP loci (Kahilainen et al. unpubl. data). To remove Illumina adapter sequences, we trimmed raw reads using Trimmomatic (Trimmomatic-0.35)[51], and normalised using Trinity v2.6.5[52]. We then used two separate procedures to construct *de novo* transcriptome assemblies, Trinity (v2.6.5) and Velvet / Oases (1.2.10)[53]. Trinity was run with standard settings, whereas Velvet / Oases used a range of nine kmer sizes (21 to 99 bp), producing a separate assembly for each kmer size. We then combined the resulting assemblies, filtered the combined assembly using the EvidentialGene (tr2aacds.pl VERSION 2017.12.21)[54] pipeline, and removed contigs smaller than 200 bp or expressed at a low level (< 1 normalized counts per million), yielding the final assembly. Transcriptome 2 was constructed from a set of 12 adult females (thorax and abdomen, without ovaries) and 48 first instar larvae, as part of a separate gene expression

study (Oostra et al. unpubl. data). RNA from these 60 individual samples was sequenced to an average depth of 16.6M reads (86/74 bp PE). The stranded RNA-seq libraries were made using Ovation® Universal RNA-Seq System (Nugen) with custom ribosomal RNA removal. The libraries were paired-end sequenced on a NextSeq 500 using the 150 bp kit (Illumina) at the DNA sequencing and genomics laboratory Institute of Biotechnology University of Helsinki. We trimmed the reads using fastp (v0.20.0)[55], and used the HISAT2 (2.0.4) / StringTie (1.3.5) pipeline[56] to construct a genome-guided transcriptome assembly, mapping the RNAseq reads to the new genome assembly.

Gene model Annotation

Initial gene predictions were obtained by running the MAKER v 2.31.10[28] gene prediction programme in an iterative procedure. In the first round of MAKER, transcriptome assembly 1, described above, was provided as evidence, and genes were predicted solely from the aligned transcripts. This resulted in 14,738 gene models. These gene models were then used for training the SNAP (2013-02-16)[57] and AUGUSTUS (3.3.2)[58] gene predictors. A second round of MAKER was run providing the *de novo* transcripts, trained gene prediction models, repeat masking file and protein data from other lepidopteran species. The MAKER settings were adjusted to allow prediction of gene models without requiring a corresponding transcript in the *de novo* transcriptome assembly. Following each round of MAKER gene prediction, the annotation completeness was assessed using BUSCO[59,60].

Manual Annotation

Manual annotation was performed for 1,232 genes, using the Apollo collaborative annotation system version 2.1.0[61]. The collaborative annotation environment was set up in Ubuntu Linux 14.04 server with 250 GB RAM and 48 AMD Opteron 6,168 processing cores. This was later upgraded to a cloud server provided by the Finnish IT Center for Science (CSC) and

running on Ubuntu Linux 18.04 with 200 GB RAM and 40 Intel Xeon model 85 processing cores. Evidence tracks were produced containing gene predictions from three rounds of MAKER, RNASeq alignments of sequence reads and protein alignments from other species (Table 2). RNASeq alignments comprised a mixed tissue pooled sample, an abdomen pooled sample and six larval samples (from transcriptome 1) selected to represent a diverse range and included, for example, both sexes and different family backgrounds.

Table 2. Evidence tracks that were used during the manual annotation of 1,232 M. cinxia genes

**Evidence track**	**Type**	**Description**
Maker 1	Gene prediction	Initial maker gene predictions based on EST alignments
Maker 2	Gene prediction	Second round of gene predictions from EST alignments, protein alignments and gene predictors trained on maker 1.
RNASeq abdomen pool	RNASeq alignment	RNASeq reads aligned to the genome with STAR
RNASeq mixed tissue pool	RNASeq alignment	
B. mori proteins	Protein alignment	Proteins sequences aligned to the genome with AAT.
H. melpomene proteins	Protein alignment	
D. melanogaster proteins	Protein alignment	
H. erato proteins	Protein alignment	
RNASeq - female larvae family 80	RNASeq alignment	RNASeq reads aligned to the genome with STAR
RNASeq - female larvae family 70	RNASeq alignment	

RNASeq female larvae family 119	RNASeq alignment	
RNASeq female larvae family 120	RNASeq alignment	
RNASeq male larvae family 80	RNASeq alignment	
RNASeq male larvae family 119	RNASeq alignment	

Final Gene Models

Following the manual annotation, the SNAP[57] and AUGUSTUS[58] gene predictors were retrained using the manually annotated gene models. MAKER was run using the updated gene predictors, transcriptome 1 and 2, and also using a masking file for repeats. As a final step to incorporate the manually annotated gene models, MAKER was run providing the previous MAKER file to pred_gff and the manually annotated models to model_gff. Gene functional prediction was performed using Pannzer v2[62].

Ortholog identification

Predicted protein sequences from *Bombyx* mori[63] (January 2017 gene models), *P. napi*[43] and *H. melpomene* (Hmel2.5)[41,42] were downloaded from silkbase http://silkbase.ab.a.u-tokyo.ac.jp/cgi-bin/download.cgi, LepBase[64] and the Butterfly Genome Database http://butterflygenome.org/?q=node/4 respectively. OrthoFinder v2.3.3[65] was run to identify orthologs between *M. cinxia*, *B. mori, P napi* and *H. melpomene* using blast as the search tool (Figure 3 & Supplementary Figure S14).

Data Validation and quality control

To assess the quality of the assembly, assembly statistics were generated using assembly-stats[64] and compared to the v1 genome as well as the *H. melpomene*, *B. mori* and *P. napi* genome assemblies (Table 1). The new genome contains 94 Mb more sequence than the previous scaffold assembly. The N50 length and L50 value at scaffold or chromosome level have improved greatly compared to the previous genome. To check for possible duplication or missing areas in the assembly, an assessment was made of the completeness of single copy orthologs from BUSCO[59,60] eukaryota, arthropoda and metazoa gene sets (Table 3). In each of the gene sets, 93.4-94.9% of the expected single copy orthologs were found in complete and single copies. The duplication rate was estimated to be between 1.4 and 2.3%. A total of 1,232 gene models were manually curated using the Apollo annotation system[61] to ensure the quality of the models. To test for contamination, the predicted protein sequences were checked with AAI-profiler[66] to identify sequences originating from different taxa (supplementary files 1-3 (AAI.html, matrix.html, krona.html)). Overall, 42% of the genome was composed of repeat sequences (Figure 4 and Supplementary Figures S15-20 (chromosome specific repeat classes)). There were no clear differences in the repeat contents between chromosomes (Supplementary Table 1). Long interspersed elements (LINE) were the most prevalent.

Table 3. BUSCO completeness estimates of the v2 genome based on the eukaryota, arthropoda and metazoa gene sets.

Lineage	BUSCO Category				
	**Complete**	**Single-copy**	**Duplicated**	**Fragmented**	**Missing**
Eukaryota	290	283	7	5	8
	95.7%	93.4%	2.3%	1.7%	2.6%

Arthropoda	1027	1012	15	7	32
	96.3%	94.9%	1.4%	0.7%	3.0%
Metazoa	935	921	14	12	31
	95.6%	94.2%	1.4%	1.2%	3.2%

Re-use potential

The substantial improvements in contiguity and gene annotation quality of the new genome will enable a range of important new studies and open up possibilities for future work. Current research aims at identifying mechanisms underlying key life history adaptations, exploring the extent of natural variation and selection on these adaptations in wild populations, and integrating these insights with the exceptional ecological, demographic, and climatic data available for this system. Future studies in this direction will help understand the mechanisms maintaining variation in life-histories across spatial and temporal scales, and on the extent to which phenotypic variation in these and other traits may contribute to a population's adaptive capacity under climate change. Several studies in different species illustrate how stress responses can be crucial for survival under variable environments, both within and between generations. The Glanville fritillary is being used to explore how environmental information is translated into adaptive phenotypic changes, and how these responses are transmitted to future generations, using transcriptomic and epigenetic approaches. Such studies will greatly benefit from an improved annotation permitting exon-specific expression quantification, and identification of epigenetic marks and other functional variants outside coding regions. Exploiting current and past large-scale sampling efforts, these new studies apply population genomic approaches that are greatly facilitated by the increased assembly contiguity, for instance by permitting linkage disequilibrium (LD) and

haplotype-based selection analyses. Other avenues of research enabled by the improved genome assembly include structural variation, regulatory evolution, recombination rate variation, and coalescent-based demographic analyses. The increasing availability of chromosome-level lepidopteran genomes such as ours permits exciting new comparative phylogenetic analyses, for example of chromosome and genome evolution.

Availability of source code and requirements (if used in the paper)

Not applicable

Availability of supporting data

The SMRT sequencing reads used for the genome assembly have been deposited to the sequence read archive under Bioproject PRJNA607899 accession number SRR11184190.

The genome has been deposited to GenBank under Bioproject PRJNA607899

The Illumina reads used for the linkage map have been deposited to the sequence read archive under Bioproject PRJNA608928 accession numbers SRR11186917- SRR11187107.

Transcriptome 1 RNASeq reads have been deposited to NCBI GEO under accession number GSE159376

Transcriptome 2 RNASeq reads have been deposited to NCBI SRA under ioproject PRJNA670126

Declarations

List of abbreviations

Not applicable

Ethics approval and consent to participate

There are no ethical policies related to working with insect data. The Glanville fritillary is not considered endangered in the Åland islands and no permits are required for sampling. However, we note that within this project the larval sampling for genetic analyses is done non-invasively in the field. ensuring insignificant demographic impact. In addition, as the sampling will take place prior diapause (Åland) when mortality is generally the highest – the collection has negligible effect on the family survival or the demography of populations.

Consent for publication

Not Applicable

Competing interests

'The authors declare that they have no competing interests'.

Funding

Funding for M.S, D.B, V.O, E.vB, J.T & A.K was provided by a grant from the European Research Council (Independent Starting Grant No. 637412 'META-STRESS' to MS) and J Ö-U, V.A and D.B from the Academy of Finland grant (Decision No. 304041 to MS & Decision No. 283108 to Ilkka Hanski). A.D was funded by a Marie Sklodowska Curie Individual Fellowship (#790531, Host Sweet Home). O-P.S. was supported by the "TTÜ development program 2016– 2022", project code 2014-2020.4.01.16-0032.

Authors' contributions

O-P.S assembled the genome, processed the chimeric contigs, performed the gap filling and the polishing of the assembly, and participated in the genome analysis.

V.A was responsible for the initial idea of the approach for the genome related activities, coordinated first part of the project, designed and produced data for the linkage map, worked on solving the haplotypes from the initial assembly.

D.B performed gene prediction, functional annotation, ortholog prediction, and managed the manual annotation.

J.K installed and managed the Apollo annotation server.

S.I was responsible for larval rearing and preparation of butterfly crosses.

P.R performed the linkage mapping and anchored the genome onto chromosomes.

V.O assembled the transcriptomes used for gene prediction.

Lo.P manually inspected the chimeric contigs.

A.R performed DNA extraction.

D.B, J.K, V.O, T.D, M.F.D, A.D, I.C.D, P.H, A.K, S.S.K, S.O.K, E.L, S.L, J.M, A.N, M.C-M, V.P, T.S, A.I.T, V.T, E.vB, J.Ö-U and M.S participated in manual annotation.

J.T performed the annotation of transposable elements and repeat classes.

L.P was responsible for the management of the DNA sequencing.

M.J.F was responsible for the management of the genome analysis.

P.A was responsible for the initial idea of the approach for the genome related activities, and the management of the genome analysis.

M.S was responsible for the management of the *M. cinxia* database and genome analysis.

D.B, O-P.S, V.A, P.R, J.T, J.K, V.O, L.P, M.J.F, P.A and M.S wrote the manuscript.

Acknowledgements

The authors wish to acknowledge CSC – IT Center for Science, Finland, for computational resources. We thank Torsti Schulz and Emily Hornett for annotating > 10 genes. We thank the personnel of the DNA sequencing and genomics laboratory (Institute of Biotechnology, Helsinki, Finland) for performing the NGS sequencing.

Authors' information

[1.] Organismal and Evolutionary Biology Research Programme, University of Helsinki, Finland

[2.] Institute of Biotechnology, University of Helsinki, Finland

[3.] Department of Chemistry and Biotechnology, Tallinn University of Technology, Estonia

[4.] Ming Wai Lau Centre for Reparative Medicine, Karolinska Institutet, Hong Kong

[5.] Natural Resource Institute (LUKE), Finland

[6.] Department of Evolution, Ecology and Behaviour, University of Liverpool, UK

[7.] Department of Biological Sciences, Louisiana State University, USA

[8.] Department of Biology, Lund University, Sweden

[9.] Department of Environmental and Biological Sciences, University of Eastern Finland, Finland

[10.] Department of Entomology and Nematology, University of Florida, USA

[11.] Department of Zoology, Loknete Vyankatrao Hiray Arts, Science & Commerce College, India

[12.] Swiss Federal Institute of Aquatic Science and Technology, Department of Aquatic Ecology, Switzerland

[13.] College of Plant Protection, China Agricultural University, China

[14.] Department of Zoology, Stockholm University, Sweden

[15.] Viikki Plant Science Centre, Organismal and Evolutionary Biology Research Programme, University of Helsinki, Finland

[16.] Helsinki Institute of Life Science (HiLIFE), University of Helsinki, Finland

Present address:

V.T - Luomus, Finnish Museum of Natural History, Zoology Unit, University of Helsinki, Helsinki, Finland

S.O.K, P.H and J.M - Biosafe – Biological Safety Solutions Ltd, Microkatu 1 M, 70210 Kuopio, Finland

corresponding author(s): ddblande@gmail.com, marjo.saastamoinen@helsinki.fi, olli-pekka.smolander@taltech.ee

Endnotes

Not applicable

Figure 1. An overview of the assembly and annotation process of the improved Glanville fritillary genome.

Figure 2. A dot-plot structural comparison of the *H. melpomene* genome against the *M. cinxia* v2 genome.

The alignment was created using D-GENIES (1.2.0)[45]. The diagonal lines indicate the collinearity between the two species. The lack of collinearity in sex chromosomes is visible in the upper left corner between Mcnxia_v2 chr 01 and Hmel2.5 chr 21. The visible vertical lines show repeats that are resolved in Mcinxia_v2 but are present in all chromosomes in Hmel2.5_chr.

Figure 3. A circos plot showing the orthologs between *M. cinxia* and *H. melpomene*

Orthologs between *M. cinxia* and *H. melpomene* were identifies using OrthoFinder and filtered for one-to-one orthologs. The internal links in the circos plot indicate the orthologs between *M. cinxia* and *H. melpomene*. The links are coloured according to the *M. cinxia* chromosome.

Figure 4. Relative amounts of different repeat classes in *M. cinxia* genome

Repeat classes and coverage of the *M. cinxia* genome v2: DNA = classII; LINE = Long interspersed elements; LTR = Long terminal repeats; LOW_COMPLEXITY = Low complexity repeated DNA; RC = Rolling circle elements (e.g. Helitrons); SINE = Short interspersed elements; Satellite = Satellite DNA; SIMPLE_REPEAT = Simple repeated motifs; EXON = Exonic regions; UNCOVERED = rest of the chromosomes.

References

1 Hanski, I. Habitat connectivity, habitat continuity, and metapopulations in dynamic landscapes. *Oikos* **87**, 209-219, doi:Doi 10.2307/3546736 (1999).

2 Ovaskainen, O. & Saastamoinen, M. Frontiers in Metapopulation Biology: The Legacy of Ilkka Hanski. *Annu Rev Ecol Evol S* **49**, 231-252, doi:10.1146/annurev-ecolsys-110617-062519 (2018).

3 Ojanen, S. P., Nieminen, M., Meyke, E., Pöyry, J. & Hanski, I. Long-term metapopulation study of the Glanville fritillary butterfly (Melitaea cinxia): survey methods, data management, and long-term population trends. *Ecol Evol* **3**, 3713-3737, doi:10.1002/ece3.733 (2013).

4 Saastamoinen, M., Hirai, N. & van Nouhuys, S. Direct and trans-generational responses to food deprivation during development in the Glanville fritillary butterfly. *Oecologia* **171**, 93-104, doi:10.1007/s00442-012-2412-y (2013).

5 Hanski, I., Saastamoinen, M. & Ovaskainen, O. Dispersal-related life-history trade-offs in a butterfly metapopulation. *J Anim Ecol* **75**, 91-100, doi:10.1111/j.1365-2656.2005.01024.x (2006).

6 Niitepõld, K. *et al.* Flight metabolic rate and Pgi genotype influence butterfly dispersal rate in the field. *Ecology* **90**, 2223-2232, doi:Doi 10.1890/08-1498.1 (2009).

7 Hanski, I. & Singer, M. C. Extinction-colonization dynamics and host-plant choice in butterfly metapopulations. *Am Nat* **158**, 341-353, doi:Doi 10.1086/321985 (2001).

8 Rosa, E., Woestmann, L., Biere, A. & Saastamoinen, M. A plant pathogen modulates the effects of secondary metabolites on the performance and immune function of an insect herbivore. *Oikos* **127**, 1539-1549, doi:10.1111/oik.05437 (2018).

9 Rosa, E., Minard, G., Lindholm, J. & Saastamoinen, M. Moderate plant water stress improves larval development, and impacts immunity and gut microbiota of a specialist herbivore. *Plos One* **14**, doi:ARTN e020429210.1371/journal.pone.0204292 (2019).

10 Salgado, A. L. & Saastamoinen, M. Developmental stage-dependent response and preference for host plant quality in an insect herbivore. *Anim Behav* **150**, 27-38, doi:10.1016/j.anbehav.2019.01.018 (2019).

11 Van Nouhuys, S. & Lei, G. C. Parasitoid-host metapopulation dynamics: the causes and consequences of phenological asynchrony. *J Anim Ecol* **73**, 526-535, doi:DOI 10.1111/j.0021-8790.2004.00827.x (2004).

12 de Jong, M. A. & Saastamoinen, M. Environmental and genetic control of cold tolerance in the Glanville fritillary butterfly. *J Evolution Biol* **31**, 636-645, doi:10.1111/jeb.13247 (2018).

13 Saastamoinen, M., Ikonen, S., Wong, S. C., Lehtonen, R. & Hanski, I. Plastic larval development in a butterfly has complex environmental and genetic causes and consequences for population dynamics. *J Anim Ecol* **82**, 529-539, doi:10.1111/1365-2656.12034 (2013).

14 Niitepõld, K. & Saastamoinen, M. A candidate gene in an ecological model species: Phosphoglucose isomerase (Pgi) in the Glanville fritillary butterfly (Melitaea cinxia). *Ann Zool Fenn* **54**, 259-273, doi:Doi 10.5735/086.054.0122 (2017).

15 de Jong, M. A., Wong, S. C., Lehtonen, R. & Hanski, I. Cytochrome P450 gene CYP337 and heritability of fitness traits in the Glanville fritillary butterfly. *Mol Ecol* **23**, 1994-2005, doi:10.1111/mec.12697 (2014).

16 Fountain, T. *et al.* Predictable allele frequency changes due to habitat fragmentation in the Glanville fritillary butterfly (vol 113, pg 2678, 2016). *P Natl Acad Sci USA* **113**, E5363-E5363, doi:10.1073/pnas.1613041113 (2016).

17 Fountain, T. *et al.* Inferring dispersal across a fragmented landscape using reconstructed families in the Glanville fritillary butterfly. *Evol Appl* **11**, 287-297, doi:10.1111/eva.12552 (2018).

18 Dileo, M. F., Husby, A. & Saastamoinen, M. Landscape permeability and individual variation in a dispersal-linked gene jointly determine genetic structure in the Glanville fritillary butterfly. *Evol Lett* **2**, 544-556, doi:10.1002/evl3.90 (2018).

19 Haag, C. R., Saastamoinen, M., Marden, J. H. & Hanski, I. A candidate locus for variation in dispersal rate in a butterfly metapopulation. *P Roy Soc B-Biol Sci* **272**, 2449-2456, doi:10.1098/rspb.2005.3235 (2005).

20 Mattila, A. L. K. & Hanski, I. Heritability of flight and resting metabolic rates in the Glanville fritillary butterfly. *J Evolution Biol* **27**, 1733-1743, doi:10.1111/jeb.12426 (2014).

21 Klemme, I. & Hanski, I. Heritability of and strong single gene (Pgi) effects on life-history traits in the Glanville fritillary butterfly. *J Evolution Biol* **22**, 1944-1953, doi:10.1111/j.1420-9101.2009.01807.x (2009).

22 Kvist, J. *et al.* Flight-induced changes in gene expression in the Glanville fritillary butterfly. *Mol Ecol* **24**, 4886-4900, doi:10.1111/mec.13359 (2015).

23 Kvist, J. *et al.* Temperature treatments during larval development reveal extensive heritable and plastic variation in gene expression and life history traits. *Mol Ecol* **22**, 602-619, doi:10.1111/j.1365-294X.2012.05521.x (2013).

24 Ahola, V. *et al.* The Glanville fritillary genome retains an ancient karyotype and reveals selective chromosomal fusions in Lepidoptera. *Nat Commun* **5**, doi:ARTN 473710.1038/ncomms5737 (2014).

25 Rastas, P., Paulin, L., Hanski, I., Lehtonen, R. & Auvinen, P. Lep-MAP: fast and accurate linkage map construction for large SNP datasets. *Bioinformatics* **29**, 3128-3134, doi:10.1093/bioinformatics/btt563 (2013).

26 Chin, C. S. *et al.* Nonhybrid, finished microbial genome assemblies from long-read SMRT sequencing data. *Nat Methods* **10**, 563-+, doi:10.1038/Nmeth.2474 (2013).

27 Chin, C. S. *et al.* Phased diploid genome assembly with single-molecule real-time sequencing. *Nat Methods* **13**, 1050-+, doi:10.1038/Nmeth.4035 (2016).

28 Campbell, M. S., Holt, C., Moore, B. & Yandell, M. Genome Annotation and Curation Using MAKER and MAKER-P. *Curr Protoc Bioinformatics* **48**, 4.11.11-39 (2014).

29 Thomas, C. D. & Hanski, I. in *Ecology, genetics and evolution of metapopulations* 489-514 (Elsevier, 2004).

30 van Bergen, E. *et al.* Summer drought decreases the predictability of local extinctions in a butterfly metapopulation. Preprint at https://doi.org/10.1101/863795 (2019).

31 Schulz, T., Vanhatalo, J. & Saastamoinen, M. Long-term demographic surveys reveal a consistent relationship between average occupancy and abundance within local populations of a butterfly metapopulation. *Ecography* **43**, 306-317, doi:10.1111/ecog.04799 (2020).

32 Salojärvi, J. *et al.* Genome sequencing and population genomic analyses provide insights into the adaptive landscape of silver birch. *Nat Genet* **49**, 904-912 (2017).

33 Li, H. Minimap and miniasm: fast mapping and de novo assembly for noisy long sequences. *Bioinformatics* **32**, 2103-2110, doi:10.1093/bioinformatics/btw152 (2016).

34 Li, H. Aligning sequence reads, clone sequences and assembly contigs with BWA-MEM. Preprint at *arXiv:1303.3997* (2013).

35 Mattila, A. L. K. *et al.* High genetic load in an old isolated butterfly population. *P Natl Acad Sci USA* **109**, E2496-E2505, doi:10.1073/pnas.1205789109 (2012).

36 Li, H. A statistical framework for SNP calling, mutation discovery, association mapping and population genetical parameter estimation from sequencing data. *Bioinformatics (Oxford, England)* **27**, 2987-2993 (2011).

37 Li, H. *et al.* The Sequence Alignment/Map format and SAMtools. *Bioinformatics (Oxford, England)* **25**, 2078-2079 (2009).

38 Huang, S., Kang, M. & Xu, A. HaploMerger2: rebuilding both haploid sub-assemblies from high-heterozygosity diploid genome assembly. *Bioinformatics (Oxford, England)* **33**, 2577-2579 (2017).

39 English, A. C. *et al.* Mind the Gap: Upgrading Genomes with Pacific Biosciences RS Long-Read Sequencing Technology. *Plos One* **7**, doi:ARTN e4776810.1371/journal.pone.0047768 (2012).

40 Walker, B. J. *et al.* Pilon: An Integrated Tool for Comprehensive Microbial Variant Detection and Genome Assembly Improvement. *Plos One* **9**, doi:ARTN e11296310.1371/journal.pone.0112963 (2014).

41 Davey, J. W. *et al.* No evidence for maintenance of a sympatric Heliconius species barrier by chromosomal inversions. *Evol Lett* **1**, 138-154, doi:10.1002/evl3.12 (2017).

42 Davey, J. W. *et al.* Major Improvements to the Heliconius melpomene Genome Assembly Used to Confirm 10 Chromosome Fusion Events in 6 Million Years of Butterfly Evolution. *G3-Genes Genom Genet* **6**, 695-708, doi:10.1534/g3.115.023655 (2016).

43 Hill, J. *et al.* Unprecedented reorganization of holocentric chromosomes provides insights into the enigma of lepidopteran chromosome evolution. *Sci Adv* **5**, doi:ARTN eaau3648 10.1126/sciadv.aau3648 (2019).

44 Kielbasa, S. M., Wan, R., Sato, K., Horton, P. & Frith, M. C. Adaptive seeds tame genomic sequence comparison. *Genome Res* **21**, 487-493, doi:10.1101/gr.113985.110 (2011).

45 Cabanettes, F. & Klopp, C. D-GENIES: dot plot large genomes in an interactive, efficient and simple way. *Peerj* **6**, doi:ARTN e495810.7717/peerj.4958 (2018).

46 Ellinghaus, D., Kurtz, S. & Willhoeft, U. LTRharvest, an efficient and flexible software for de novo detection of LTR retrotransposons. *BMC Bioinformatics* **9**, doi:Artn 1810.1186/1471-2105-9-18 (2008).

47 Steinbiss, S., Willhoeft, U., Gremme, G. & Kurtz, S. Fine-grained annotation and classification of de novo predicted LTR retrotransposons. *Nucleic Acids Res* **37**, 7002-7013, doi:10.1093/nar/gkp759 (2009).

48 Fu, L. M., Niu, B. F., Zhu, Z. W., Wu, S. T. & Li, W. Z. CD-HIT: accelerated for clustering the next-generation sequencing data. *Bioinformatics* **28**, 3150-3152, doi:10.1093/bioinformatics/bts565 (2012).

49 Jurka, J. Repbase Update - a database and an electronic journal of repetitive elements. *Trends Genet* **16**, 418-420, doi:Doi 10.1016/S0168-9525(00)02093-X (2000).

50 Hubley, R. *et al.* The Dfam database of repetitive DNA families. *Nucleic Acids Res* **44**, D81-D89, doi:10.1093/nar/gkv1272 (2016).

51 Bolger, A. M., Lohse, M. & Usadel, B. Trimmomatic: a flexible trimmer for Illumina sequence data. *Bioinformatics (Oxford, England)* **30**, 2114-2120 (2014).

52 Grabherr, M. G. *et al.* Full-length transcriptome assembly from RNA-Seq data without a reference genome. *Nat Biotechnol* **29**, 644-652 (2011).

53 Schulz, M. H., Zerbino, D. R., Vingron, M. & Birney, E. Oases: robust de novo RNA-seq assembly across the dynamic range of expression levels. *Bioinformatics (Oxford, England)* **28**, 1086-1092 (2012).

54 Gilbert, D. Gene-omes built from mRNA-seq not genome DNA. (2013).

55 Chen, S., Zhou, Y., Chen, Y. & Gu, J. fastp: an ultra-fast all-in-one FASTQ preprocessor. *Bioinformatics (Oxford, England)* **34**, i884-i890 (2018).

56 Pertea, M., Kim, D., Pertea, G. M., Leek, J. T. & Salzberg, S. L. Transcript-level expression analysis of RNA-seq experiments with HISAT, StringTie and Ballgown. *Nat Protoc* **11**, 1650-1667 (2016).

57 Korf, I. Gene finding in novel genomes. *BMC Bioinformatics* **5**, 59 (2004).

58 Lomsadze, A., Ter-Hovhannisyan, V., Chernoff, Y. O. & Borodovsky, M. Gene identification in novel eukaryotic genomes by self-training algorithm. *Nucleic Acids Res* **33**, 6494-6506 (2005).

59 Simao, F. A., Waterhouse, R. M., Ioannidis, P., Kriventseva, E. V. & Zdobnov, E. M. BUSCO: assessing genome assembly and annotation completeness with single-copy orthologs. *Bioinformatics* **31**, 3210-3212, doi:10.1093/bioinformatics/btv351 (2015).

60 Waterhouse, R. M. *et al.* BUSCO Applications from Quality Assessments to Gene Prediction and Phylogenomics. *Mol Biol Evol* **35**, 543-548, doi:10.1093/molbev/msx319 (2018).

61 Dunn, N. A. *et al.* Apollo: Democratizing genome annotation. *PLoS Comput Biol* **15**, doi:ARTN e100679010.1371/journal.pcbi.1006790 (2019).

62 Törönen, P., Medlar, A. & Holm, L. PANNZER2: a rapid functional annotation web server. *Nucleic Acids Res* **46**, W84-W88, doi:10.1093/nar/gky350 (2018).

63 Kawamoto, M. *et al.* High-quality genome assembly of the silkworm, Bombyx mori. *Insect Biochem Mol Biol* **107**, 53-62, doi:10.1016/j.ibmb.2019.02.002 (2019).

64 Challis, R. J., Kumar, S., Dasmahapatra, K. K., Jiggins, C. D. & Blaxter, M. Lepbase: the Lepidopteran genome database. Preprint at https://www.biorxiv.org/content/10.1101/056994v1.abstract doi:10.1101/056994 (2016).

65 Emms, D. M. & Kelly, S. OrthoFinder: phylogenetic orthology inference for comparative genomics. *Genome Biol* **20**, doi:ARTN 23810.1186/s13059-019-1832-y (2019).

66 Medlar, A. J., Törönen, P. & Holm, L. AAI-profiler: fast proteome-wide exploratory analysis reveals taxonomic identity, misclassification and contamination. *Nucleic Acids Res* **46**, W479-W485, doi:10.1093/nar/gky359 (2018).

67 Dobin, A. *et al.* STAR: ultrafast universal RNA-seq aligner. *Bioinformatics* **29**, 15-21, doi:10.1093/bioinformatics/bts635 (2013).

68 Slater, G.S and Birney, E. Automated generation of heuristics for biological sequence comparison. *BMC Bioinformatics* **6:31**; doi: 10.1186/1471-2105-6-31 (2005).

Figure

Click here to access/download;Figure;Fig1.png

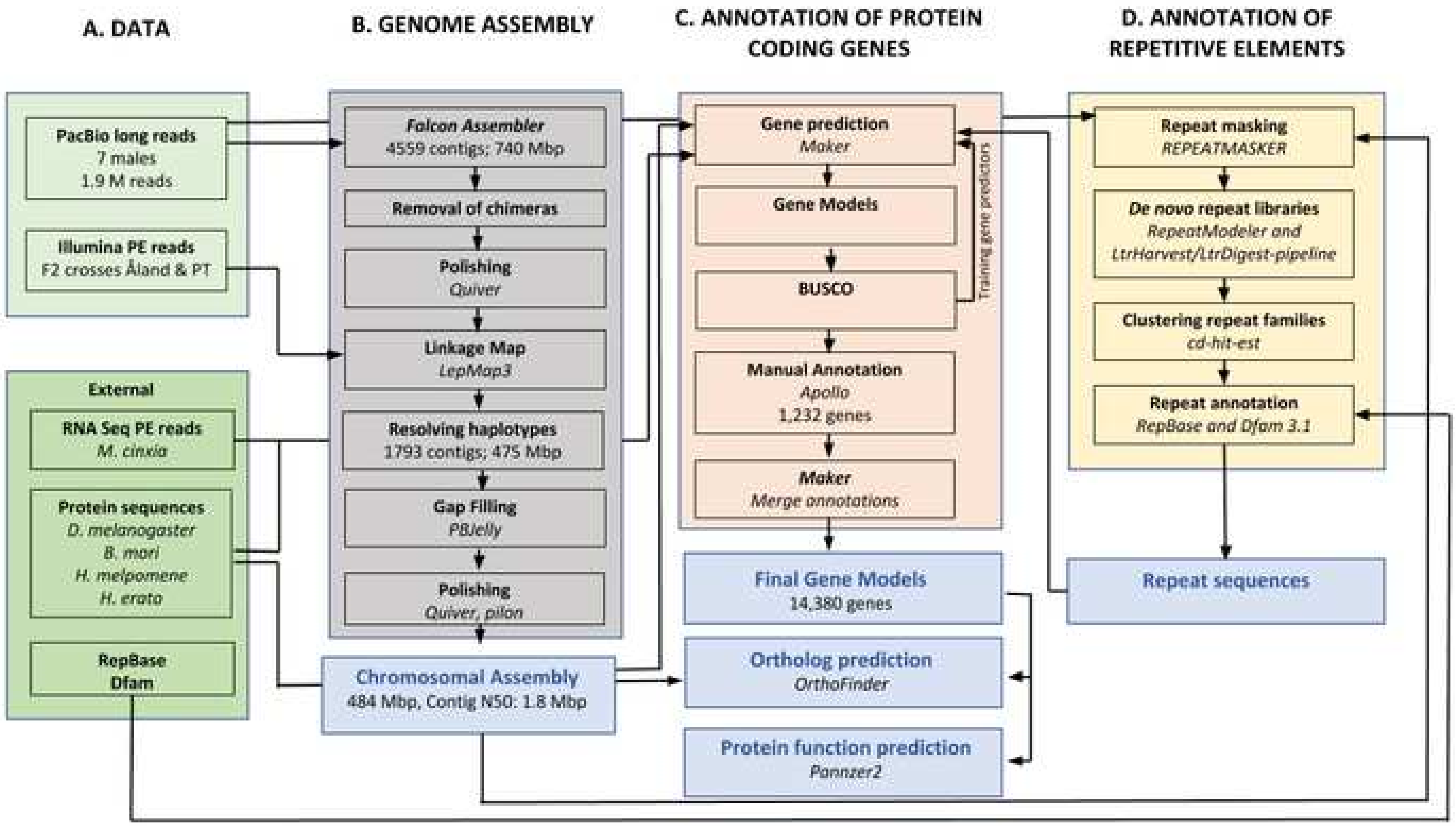

Figure Click here to access/download;Figure;Fig2.png

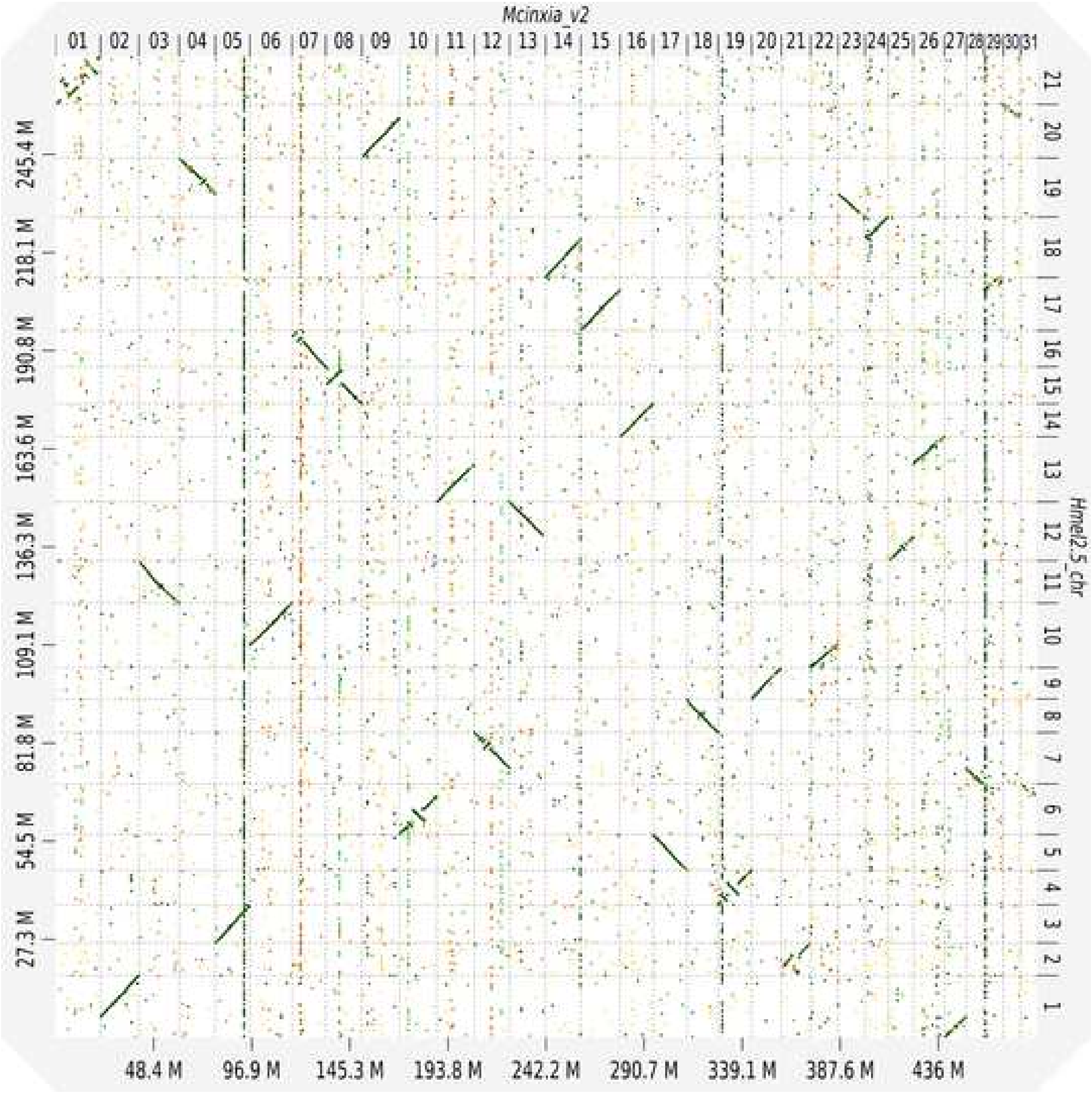

Figure

Click here to access/download;Figure;Fig3.png

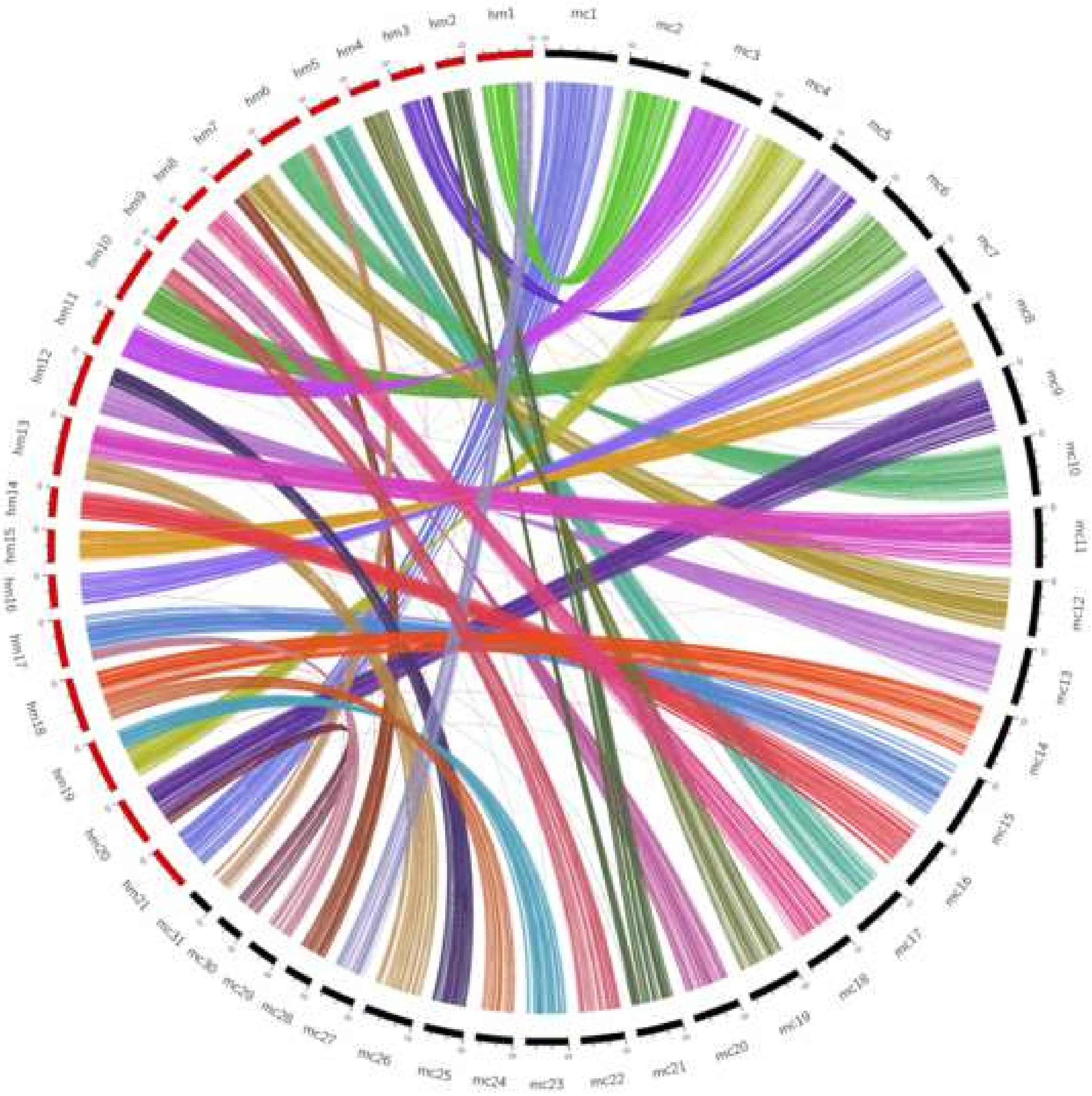

Figure

Click here to access/download;Figure;Fig4.png

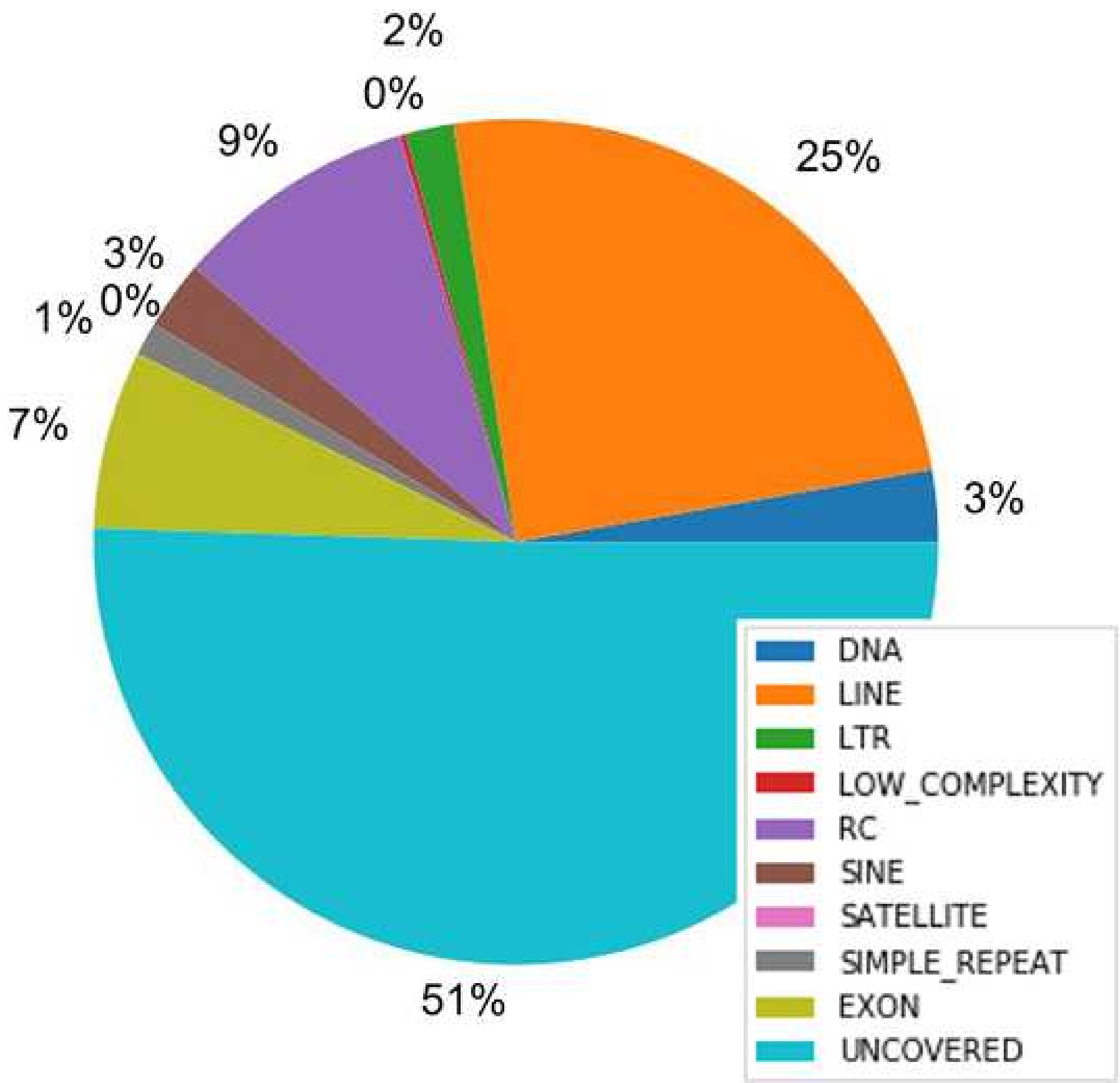

Click here to access/download
Supplementary Material
Supplementary_Figures.docx

Supplementary Material

Click here to access/download

Supplementary Material

AAI.html

Click here to access/download
Supplementary Material
krona.html

Click here to access/download
Supplementary Material
matrix.html

Click here to access/download
Supplementary Material
Supplementary_table1.xlsx
