# Supplementary material for: Improved chromosome-level genome assembly of the Glanville fritillary butterfly (Melitaea cinxia) integrating Pacific Biosciences long reads and a high-density linkage map
Source: Gigascience. 2022 Jan 12;11:giab097. doi: 10.1093/gigascience/giab097 (PMC8756199; doi:10.1093/gigascience/giab097)
Supplement: giab097_GIGA-D-20-00318_Revision_1 [file giab097_giga-d-20-00318_revision_1.pdf]

GigaScience

Improved chromosome-level genome assembly of the Glanville fritillary butterfly (Melitaea cinxia) integrating PacBio long reads and a high-density linkage map.

--Manuscript Draft--

**Manuscript Number:**	GIGA-D-20-00318R1
**Full Title:**	Improved chromosome-level genome assembly of the Glanville fritillary butterfly (Melitaea cinxia) integrating PacBio long reads and a high-density linkage map.
**Article Type:**	Data Note
**Funding Information:**	H2020 European Research Council (637412) — Dr Marjo Saastamoinen<br>Academy of Finland (304041) — Dr Marjo Saastamoinen<br>Academy of Finland (283108) — Not applicable<br>H2020 Marie Skłodowska-Curie Actions (790531) — Ms Anne Duploy<br>Sihtasutus Archimedes (2014-2020.4.01.16-0032) — Not applicable
**Abstract:**	The Glanville fritillary (Melitaea cinxia) butterfly is a long-term model system for metapopulation dynamics research in fragmented landscapes. Here, we provide a chromosome level assembly of the butterfly’s genome produced from Pacific Biosciences sequencing of a pool of males, combined with a linkage map from population crosses. The final assembly size of 484 Mb is an increase of 94 Mb on the previously published genome. Estimation of the completeness of the genome with BUSCO, indicates that the genome contains 93 - 95% of the BUSCO genes in complete and single copies. We predicted 14,830 gene models using the MAKER pipeline and manually curated 1,232 of these gene models. The genome and its annotated gene models are a valuable resource for future comparative genomics, molecular biology, transcriptome and genetics studies on this species.
**Corresponding Author:**	Olli-Pekka Smolander<br>Tallinn University of Technology<br>Tallinn, Harju ESTONIA
**Corresponding Author Secondary Information:**	
**Corresponding Author's Institution:**	Tallinn University of Technology
**Corresponding Author's Secondary Institution:**	
**First Author:**	Olli-Pekka Smolander
**First Author Secondary Information:**	
**Order of Authors:**	Olli-Pekka Smolander
	Daniel Blande
	Virpi Ahola
	Pasi Rastas
	Jaakko Tanskanen
	Juhana I Kammonen
	Vincencio Oostra
	Lorenzo Pellegrini
	Suvi Ikonen
	Tad Dallas

Powered by Editorial Manager® and ProduXion Manager® from Aries Systems Corporation

	Michelle F DiLeo
	Anne Duploy
	Ilhan Cem Duru
	Pauliina Halimaa
	Aapo Kahilainen
	Suyog S Kuwar
	Sirpa O Kärenlampi
	Elvira Lafuente
	Shiqi Luo
	Jenny Makkonen
	Abhilash Nair
	Maria de la Paz Celorio-Mancera
	Ville Pennanen
	Annukka Ruokolainen
	Tarja Sundell
	Arja I Tervahauta
	Victoria Twort
	Erik van Bergen
	Janina Österman-Udd
	Lars Paulin
	Mikko J Frilander
	Petri Auvinen
	Marjo Saastamoinen
**Order of Authors Secondary Information:**	
**Response to Reviewers:**	We thank the reviewers for their invaluable comments and their work on reviewing this manuscript that helped us to further improve our work. We have addressed their concerns and made several additions and changes to the manuscript according to their suggestions. We have highlighted all the changes and added text in the manuscript. Detailed description of the changes is below with the context of reviewer comments:<br><br>Reviewer reports:<br><br>Reviewer #1: In this manuscript, Blande, Smolander and colleagues report an improved chromosome-level genome assembly of the important ecological model lepidopteran species Melitaea cinxta. The manuscript would benefit from further language review by a native English speaker to improve readability, but the intentions of the authors are nevertheless clearly articulated throughout, the workflow is logical, and the assembly quality is a clear improvement on the earlier draft release.<br><br>I would suggest revisiting the title to better reflect the work- as it stands it is a little underwhelming. One suggestion would be "Improved chromosome-level genome assembly of the Glanville fritillary butterfly (Melitaea cinxia) integrating PacBio long reads and a high-density linkage map". I would ideally also like to see more discussion of the more unusual aspects of this project- for example, long-read assemblies are commonplace now, but the linkage map approach (and the extent to which there was manual curation of potential chimeric scaffolds) is less frequently employed these days and often super-scaffolding and error correction is undertaken with Hi-C methods only. Similarly the extensive manual curation of gene annotations and the impact that this had on the models is likely of more general interest (e.g. how many gene models were

corrected, what type of errors were encountered?). Particularly also some mention of some of the specific challenges of this project
(e.g. the need to combine multiple individuals to obtain sufficient quantities of gDNA) might be interesting for the readership.

We thank the reviewer for the suggestion. We have revised the title accordingly. We have also added parts discussing the use and benefit of linkage map and the extensive manual curation of gene models as well as clarified the challenge of using multiple individuals for DNA.

The absence of line numbers is a little cumbersome for reviewing purposes, I'll below refer to specific parts of the text by page number (as printed on pdf document) - paragraph -line(within paragraph).

We have now added line numbers to allow accurate and easy referencing to manuscript while reviewing.

3-1-6: suggest changing ".... and included both laboratory and natural environmental conditions" to "…and have included…"

We have modified the text accordingly.

3-2-1: change "The first M. cinxia genome was released in 2014" to "The first M. cinxia draft genome" or "The first M. cinxia genome assembly"

We have again modified the text accordingly.

Table1: reporting both GC and AT % is unnecessary. There are some discrepancies between the statistics reported for the chromosomal assembly in the Ahola et al (2014) paper vs this table. This may simply be due to different methods for assessing summary statistics (e.g. whether or not gaps are included by default), but warrants investigation/clarification. For example, the largest scaffold reported in the Ahola et al (2014) paper is 14,178,551bp. The description of the generation of a chromosomal build for the previous version indicates >280Mb were assigned to chromosomes, whereas the total assembly size in this table is reported to be only 251Mb.

We thank the reviewer for pointing this out. We have also removed the version 1 chromosomes from Table 1 and modified the text accordingly. The chromosome file that was used in constructing the table statistics was not published with the version 1 of the genome and, additionally, it is not well representative of the genome so we chose to omit the comparison. We keep the published version 1 scaffolds in the comparison which highlights even more the improvement over the previously published genome.

6-2-2: What are the units for the cut-off (read length?)? If available, the data exploring the impact of different cut-offs on the assembly error rate could be of interest to others assembling genomes de novo.

We thank the reviewer for pointing out this inaccuracy. We have modified the text on genome assembly accordingly to include information about the read length. We have also added text clarifying the procedure that was used to detect and split chimeric contigs and added a general error statistic.

6-2-6: As a specific example of a more general comment on number reporting, perhaps state 24.4 Gb instead of 24,409,505,551 bp? I am not sure that the precision is always necessary and scaling/rounding can help readability.

We thank the reviewer for this suggestion and we have modified the text accordingly.

6-2-10: Are the alternative contigs extracted by default by the FALCON pipeline? Are there any adjustments that need to be made for an input of >1 individual, for example?

We have added the information about alternative contig extraction: it was done by FALCON automatically. The default FALCON pipeline was also used without any

further modifications.

7-2-2: The raw data for the linkage map crosses, and also the RNAseq data for the transcriptome studies (on) is described as "unpublished", but I believe public sequence accessions are also being released with this manuscript. Is there additional information that would need to be disclosed for this information to be utilised by others or is the intention to highlight that the data will also be presented in upcoming publications?

We have removed the mention about the F2 data being unpublished at this point and later in the manuscript clarified the text concerning the RNAseq data to make clear that all the data is being released with the manuscript but has been produced in separate studies and will be further analysed in them on their own. We have also added a citation to the study by Kahilainen et al. which has now been deposited to biorxiv.

7-2-6 "Part of" should be "Some of"

We have modified the text accordingly.

7-3-3: Specify "relative humidity" instead of RH.
Discuss why different approaches used for different RNAseq experiments.

We have modified the text accordingly. The use of different approaches in RNAseq experiments has also been described now in more detail.

8-1-5: Sequencing was "performed" rather than "made". Can you specify which HiSeq model and which sequencing library kit (or at the very least whether it was PCR-free)?

We have added the information about the instrument model (2000) and mention of standard PE protocol for sequencing library kit. So no PCR-free kit was used.

9-2-6: Presumably "de novo transcripts" refers to both transcriptomes 1 and 2, in which case I think it would be helpful to state this here. I assume the different analysis approaches for datasets 1 and 2 reflect different histories of the two datasets but it would be interesting to see some assessment of the relative performances of these approaches.

We thank the reviewer for pointing out the need for clarification here. We have improved the description of the RNAseq experiments and added some statistics of the results.

13-2-4: I think that http://butterflygenome.org would be sufficient for the URL here.

We have modified the text according to the reviewer's suggestion.

14-1-4: Are there any flow cytometry (or other) estimates of genome size that can be used to set alongside the v1 and v2 assembly sizes?

We have added the information about k-mer based genome size estimate to the manuscript and added supplementary data to explain the procedure. We have also compared the estimates to the obtained assembly size, which is well in line with them.

Reviewer #2: The authors presented us with an improved genome for Glanville fritillary butterfly. However, there are several issues that need to be addressed before its acceptance.

Major:

What the current manuscript lacks the most is the comparison between the improved

genome assembly and its former version. Although the authors showed us an improved N50, I failed to find the explanations for several critical differences. For example, (1) the authors stated that ca. 90 MB additional assembly sequences were achieved, but no further information is available for those new sequences, are they redundancies or missed fragments in the version 1; (2) the improved genome predicted less genes compared to its former version, decreasing from ca. 16,000 genes to ~ 14,000 genes, which is contradictory to the aforementioned longer genome assembly; (3) the former genome version observed unevenly distributed repeat elements across chromosomes, while not in this improved one, which also needs explanations.

We thank the reviewer for pointing out the need for these comparisons. We have added text to highlight the differences between the version 1 and version 2 genome and explaining the observed differences. The decreasing number of genes is likely to be caused by less fragmented gene models due to the improved assembly. Many fragmented gene models and frameshifts were observed in the manual annotation of the genes in version 1. The BUSCO estimates also support the claim that the approximately 14,700 genes identified in the version 2 assembly reflect well the actual reality.
The added sequence of version 2 and the even distribution of repeat elements both show the issue of collapsed repeats in version 1. We see this also when observing individual alignments in full genome alignment between version 1 and 2 where many areas of version 1 are aligned to several positions and chromosomes in version 2. Below is an example of one such alignment group from version 1 to version 2:

scaffold1689 668473 40307 41078 - M21_B16_H02 14417991 14404121 14404897 623 776 18 tp:A:P cm:i:48 s1:i:622 s2:i:571 dv:f:0.0213 rl:i:41165
scaffold1689 668473 40307 41078 - M03_B15_H11 19852298 15915449 15916225 572 776 0 tp:A:S cm:i:43 s1:i:571 dv:f:0.0271 rl:i:41165
scaffold1689 668473 40353 41078 + M11_B22_H13a 18534680 8043072 8043801 562 730 0 tp:A:S cm:i:42 s1:i:561 dv:f:0.0269 rl:i:41165
scaffold1689 668473 40307 41078 + M13_B08_H12a 17792744 16168014 16168790 557 776 0 tp:A:S cm:i:42 s1:i:556 dv:f:0.0284 rl:i:41165
scaffold1689 668473 40307 41078 - M22_B28_H10b 13644878 615914 616690 556 776 0 tp:A:S cm:i:43 s1:i:555 dv:f:0.0271 rl:i:41165
scaffold1689 668473 40342 41078 - M13_B08_H12a 17792744 14324285 14325025 555 741 0 tp:A:S cm:i:43 s1:i:554 dv:f:0.0264 rl:i:41165
scaffold1689 668473 40307 41078 + M11_B22_H13a 18534680 1239116 1239892 546 776 0 tp:A:S cm:i:41 s1:i:545 dv:f:0.0296 rl:i:41165
scaffold1689 668473 40307 41078 - M23_B26_H19b 13251621 10196154 10196929 540 776 0 tp:A:S cm:i:41 s1:i:539 dv:f:0.0296 rl:i:41165
scaffold1689 668473 40307 41078 + M04_B12_H19a 17855385 8285805 8286582 538 777 0 tp:A:S cm:i:39 s1:i:537 dv:f:0.0323 rl:i:41165
scaffold1689 668473 40307 41078 - M13_B08_H12a 17792744 14934132 14934910 537 778 0 tp:A:S cm:i:38 s1:i:536 dv:f:0.0336 rl:i:41165
scaffold1689 668473 40307 41078 + M29_B24b_H17b 9593829 8608573 8609349 536 776 0 tp:A:S cm:i:38 s1:i:535 dv:f:0.0336 rl:i:41165
scaffold1689 668473 40342 41078 - M24_B27_H18b 11526678 5031139 5031880 531 741 0 tp:A:S cm:i:38 s1:i:530 dv:f:0.0329 rl:i:41165
scaffold1689 668473 40307 41078 - M03_B15_H11 19852298 6053639 6054414 526 776 0 tp:A:S cm:i:40 s1:i:525 dv:f:0.0309 rl:i:41165
scaffold1689 668473 40307 41078 + M11_B22_H13a 18534680 13085188 13085964 524 776 0 tp:A:S cm:i:38 s1:i:523 dv:f:0.0336 rl:i:41165

Powered by Editorial Manager® and ProduXion Manager® from Aries Systems Corporation

	scaffold1689 668473 40342 41078 + M18_B25_H08 15256489 10835487 10836227 519 743 0 tp:A:S cm:i:38 s1:i:518 dv:f:0.0329 rl:i:41165<br>scaffold1689 668473 40307 41078 + M01_B01_H21 22190643 5160201 5160952 519 776 0 tp:A:S cm:i:40 s1:i:512 dv:f:0.0309 rl:i:41165<br>scaffold1689 668473 40307 41078 - M19_B21_H04 16576953 11089111 11089891 512 780 0 tp:A:S cm:i:38 s1:i:511 dv:f:0.0336 rl:i:41165<br>scaffold1689 668473 40342 41078 - M09_B10_H20a 18518805 10342385 10343125 512 740 0 tp:A:S cm:i:38 s1:i:511 dv:f:0.0329 rl:i:41165<br>scaffold1689 668473 40342 41044 - M05_B06_H03 17119925 7922038 7922742 513 708 0 tp:A:S cm:i:38 s1:i:511 dv:f:0.0306 rl:i:41165<br>scaffold1689 668473 40307 41078 - M01_B01_H21 22190643 11229175 11229951 510 776 0 tp:A:S cm:i:38 s1:i:509 dv:f:0.0336 rl:i:41165<br>scaffold1689 668473 40307 41078 + M01_B01_H21 22190643 8298393 8299169 510 776 0 tp:A:S cm:i:38 s1:i:509 dv:f:0.0336 rl:i:41165<br>scaffold1689 668473 40307 41078 + M06_B05_H10a 20826893 9408332 9409105 510 776 0 tp:A:S cm:i:38 s1:i:509 dv:f:0.0336 rl:i:41165<br>scaffold1689 668473 40307 41078 - M22_B28_H10b 13644878 12441833 12442607 510 777 0 tp:A:S cm:i:38 s1:i:509 dv:f:0.0336 rl:i:41165<br>scaffold1689 668473 40307 41052 - M04_B12_H19a 17855385 13621791 13622541 509 750 0 tp:A:S cm:i:38 s1:i:508 dv:f:0.0322 rl:i:41165<br>scaffold1689 668473 40307 41078 - M12_B11a_H07a 17359406 16022782 16023558 508 776 0 tp:A:S cm:i:37 s1:i:507 dv:f:0.0350 rl:i:41165<br>scaffold1689 668473 40307 41059 + M24_B27_H18b 11526678 2180441 2181195 507 757 0 tp:A:S cm:i:38 s1:i:506 dv:f:0.0329 rl:i:41165<br>scaffold1689 668473 40307 41078 + M16_B19_H14 16354949 2929659 2930435 506 776 0 tp:A:S cm:i:38 s1:i:505 dv:f:0.0336 rl:i:41165<br>scaffold1689 668473 40307 40975 + M15_B13_H17a 19434445 3062681 3063356 506 675 0 tp:A:S cm:i:38 s1:i:505 dv:f:0.0240 rl:i:41165<br>scaffold1689 668473 40307 41052 + M18_B25_H08 15256489 10031389 10032138 505 749 0 tp:A:S cm:i:38 s1:i:504 dv:f:0.0322 rl:i:41165<br>scaffold1689 668473 40342 41078 - M11_B22_H13a 18534680 16915687 16916427 504 740 0 tp:A:S cm:i:38 s1:i:503 dv:f:0.0329 rl:i:41165<br>scaffold1689 668473 40342 41078 - M14_B23a_H18a 17331753 12110681 12111424 504 743 0 tp:A:S cm:i:40 s1:i:503 dv:f:0.0302 rl:i:41165<br>scaffold1689 668473 40307 41030 - M01_B01_H21 22190643 1375513 1376237 503 728 0 tp:A:S cm:i:37 s1:i:502 dv:f:0.0313 rl:i:41165<br>scaffold1689 668473 40307 41078 - M18_B25_H08 15256489 8205547 8206325 503 778 0 tp:A:S cm:i:33 s1:i:502 dv:f:0.0411 rl:i:41165<br>scaffold1689 668473 40307 41078 + M03_B15_H11 19852298 17422003 17422788 502 786 0 tp:A:S cm:i:37 s1:i:499 dv:f:0.0350 rl:i:41165<br>scaffold1689 668473 40307 41078 + M27_B24a_H01b 10766681 833012 833789 499 777 0 tp:A:S cm:i:39 s1:i:499 dv:f:0.0323 rl:i:41165<br>scaffold1689 668473 40342 41052 - M02_B04_H01a 18938283

Powered by Editorial Manager® and ProduXion Manager® from Aries Systems Corporation

2532309 2533024 499 715 0 tp:A:S cm:i:37 s1:i:498 dv:f:0.0328 rl:i:41165

However, we feel that a detailed investigation of the additional sequence of version 2 compared to version 1 is outside of the scope of this current data note manuscript but we will further elucidate it in a research work concentrating on the repeat structure of the genome and structural variation between the individuals.

Another important issue of the present manuscript is the confusion introduced by varied genome assembly sizes. Firstly, the authors did not provide this critical information that can be estimated using several well-known methods, such as C value based on flow cytometry, or estimations based on kmer frequency information. Secondly, the author firstly mentioned that they sampled individuals that have low heterozygosity, but later the FALCON generated an assembly almost twice the size of the final genome. The authors may want to add extra analysis or words to clarify the genome size uncertainty.

We have added the information about k-mer based genome size estimate to the manuscript and added supplementary data to explain the procedure. We have also compared the estimates to the obtained assembly size, which is well in line with them. We have also clarified the text about initial “low heterozygosity” and the high assembly size due to the use of several (7) individuals and their differing haplotypes.

Same to the above concern, Haplomerge seems an important step to obtain the final version assembly, and if I understand it correctly, the authors did not use a standardized analysis pipeline, please consider to include a schematic plot for your procedure to help readers better understand your steps and the principle behind them.

We thank the reviewer for pointing out unclarity in this part of the manuscript. Haplomerger (v2) was used only to calculate alignment chains (contig-contig alignments) to find haplotypes. (We tried running HaploMerger all the way but it only removed about 30Mb, about 10% of haplotypes.)

In addition, lots of methods are vaguely described, the authors should provide details for them to make sure the analyses are repeatable, e. g. on Page 6, the authors wrote: "This cut-off was experimentally found to give the best contiguity for the assembly, while minimizing (within a small margin of error) the percentage of possibly erroneous contigs". But I failed to find any details of their experiments. And on the same page, the authors checked putative chimerics manually, saying the error regions are with low coverage or repeat regions, the authors should give demonstration examples and statistics for different kinds of errors. Meanwhile, when they say the error regions were split, the authors should give details about how they determined the split positions since what they found are error regions instead of error bases. Also, on page 7, the authors stated "The contigs orders and orientations were manually fixed when needed", please list the different situations that meet your criteria.

We thank the reviewer for pointing this out. We have clarified the text about genome assembly and improvement. We have also cleared the text about linkage map.
Here the orientation was fixed if the map had support for alternative orientation or the contig-contig alignments were linking contigs together. Since this genome work, an automatic pipeline has been published in the article "Lep-Anchor: automated construction of linkage map anchored haploid genomes, Bioinformatics, 36:8 (2020)"

The author may want to explain why they choose the 1,232 genes for manual annotation. Random?

We thank the reviewer for pointing out the need for further explanation here. We have added the text for explaining the choice of prioritized gene families and allocation of the genes for annotators. The number of genes was limited by practical reasons (limited

Powered by Editorial Manager® and ProduXion Manager® from Aries Systems Corporation

	amount of time, limited number of annotators).<br><br>Minor:<br><br>Remove "(e.g. Kahilainen et al. unpubl.)", it provides no useful information.<br><br>We have modified the text according to the reviewer's suggestion and added a reference to the recently submitted biorxiv pre-print.<br><br>Table 1. N(%) of the verion 2 genome is zero? The scaffolding step does not introduce any Ns? I doubt that.<br><br>We have modified the text according to reviewers suggestion and added '<0.01' to the table<br><br>Page 5, please give the location information instead of a citation.<br><br>We have modified the text according to the reviewer's suggestion and added the location information and removed the redundant citation.<br><br>Page 7, please clarify the assembly version for raw read mapping, is it the one generated by FALCON with a genome size ~ 700 MB?<br><br>We have modified the text according to the reviewer's suggestion and made it clear that the primary assembly with that size from FALCON was used.<br><br>Page 9, "the first two steps (bath A1 and bath A2)", please provide biological explanations.<br><br>We have rephrased this " running the first two steps (batch A and B to calculate the alignment chain)" to make it clear that it is part of the HaploMerger2 pipeline that was used to initiate the process.<br><br>Marey map needs citation and a brief explanation of its debut.<br><br>We thank the reviewer for this suggestion. We have added a citation and a brief description of Marey map for clarity.<br><br>"In M. cinxia the repeats are placed in single chromosomes whereas in H. melpomene they are present in all chromosomes. " How does it help to show the power of long read assembly? Need explanation.<br><br>We thank the reviewer for this suggestion. We have added the text "With long reads spanning the repeats and allowing their accurate placement in the contigs," to clarify this statement.<br><br>Page 10, how does Velvet apply a kmer size of 99 bp when you only have a read length as long as 85 bp?<br><br>We thank the reviewer for pointing out this error. This was a mistake in the text and the statement about the used k-mer size range has been corrected. The Velvet was only run up to 71 pb.<br><br>Table 2 title: species name should be in format of italic.<br><br>We thank the reviewer for pointing this out. We have modified the text accordingly.<br><br>Please give a full name for BUSCO in its first appearance.<br><br>We thank the reviewer for pointing this out. We have again modified the text accordingly.
**Additional Information:**	

Question	Response
Are you submitting this manuscript to a special series or article collection?	No
**Experimental design and statistics**<br><br>Full details of the experimental design and statistical methods used should be given in the Methods section, as detailed in our Minimum Standards Reporting Checklist. Information essential to interpreting the data presented should be made available in the figure legends.<br><br>Have you included all the information requested in your manuscript?	Yes
**Resources**<br><br>A description of all resources used, including antibodies, cell lines, animals and software tools, with enough information to allow them to be uniquely identified, should be included in the Methods section. Authors are strongly encouraged to cite Research Resource Identifiers (RRIDs) for antibodies, model organisms and tools, where possible.<br><br>Have you included the information requested as detailed in our Minimum Standards Reporting Checklist?	Yes
**Availability of data and materials**<br><br>All datasets and code on which the conclusions of the paper rely must be either included in your submission or deposited in publicly available repositories (where available and ethically appropriate), referencing such data using a unique identifier in the references and in the "Availability of Data and Materials" section of your manuscript.	Yes

Have you have met the above requirement as detailed in our Minimum Standards Reporting Checklist?	

Manuscript
Click here to access/download;Manuscript;Cinxia_genome_manuscript_giga

Title page

Improved chromosome-level genome assembly of the Glanville fritillary butterfly (Melitaea cinxia) integrating PacBio long reads and a high-density linkage map.

Olli-Pekka Smolander*[1,3], Daniel Blande[*2], Virpi Ahola[2,4], Pasi Rastas[1], Jaakko Tanskanen[5], Juhana I. Kammonen[1], Vicencio Oostra[2,6], Lorenzo Pellegrini[1], Suvi Ikonen[2], Tad Dallas[7], Michelle F. DiLeo[2], Anne Duplouy[2,8], Ilhan Cem Duru[1], Pauliina Halimaa[9], Aapo Kahilainen[2], Suyog S. Kuwar[10,11], Sirpa O. Kärenlampi[9], Elvira Lafuente[12], Shiqi Luo[13], Jenny Makkonen[9], Abhilash Nair[2], Maria de la Paz Celorio-Mancera[14], Ville Pennanen[15], Annukka Ruokolainen[2], Tarja Sundell[1], Arja I. Tervahauta[9], Victoria Twort[8], Erik van Bergen[2], Janina Österman-Udd[2], Lars Paulin[1], Mikko J. Frilander[#1], Petri Auvinen[#1], Marjo Saastamoinen[#2,16]

* These authors contributed equally to the work

# These authors contributed equally to the work

Abstract

The Glanville fritillary (*Melitaea cinxia*) butterfly is a long-term model system for metapopulation dynamics research in fragmented landscapes. Here, we provide a chromosome level assembly of the butterfly's genome produced from Pacific Biosciences sequencing of a pool of males, combined with a linkage map from population crosses. The final assembly size of 484 Mb is an increase of 94 Mb on the previously published genome. Estimation of the completeness of the genome with Benchmarking Universal Single-Copy Orthologs (BUSCO), indicates that the genome contains 93 - 95% of the BUSCO genes in

complete and single copies. We predicted 14,830 gene models using the MAKER pipeline and manually curated 1,232 of these gene models. The genome and its annotated gene models are a valuable resource for future comparative genomics, molecular biology, transcriptome and genetics studies on this species.

Keywords

Melitaea cinxia, Glanville fritillary, Genome, Spatial Ecology

Data Description

Context

Identifying and characterizing genes underlying ecologically and evolutionarily relevant phenotypes in natural populations has become possible with novel genomic tools that can also be utilized in 'non-model' organisms. The Glanville fritillary (*Melitaea cinxia*) butterfly, and in particular its metapopulation in the Åland Islands (SW Finland), is an ecological model system in spatial ecology[1,2]. In short, within this archipelago, the species inhabits a network of dry outcrop meadows and pastures, and persists as a classic metapopulation with high turnover in patch occupancy[1]. The network of 4,500 potential habitat patches has been systematically surveyed bi-annually for butterfly occupancy and abundance since 1993[3], providing a vast amount of ecological data on population dynamics[2]. Experimental manipulations under more controlled conditions are also possible due to the small size, high fecundity and relatively short generation time of the species. Consequently, the ecological understanding of the species expands to the life history ecology across development stages[4,5], dispersal dynamics[6,7], species interactions (host plants and parasitoids)[8-12],

and stress tolerance[13,14]. During the last decade, the system has also been used to study genetic and evolutionary processes, such as identifying candidate genes underlying variation and evolution of dispersal in fragmented habitats[15] and host plant preference[16], and assessing allelic variation and their dynamics in space and time with SNP data across populations[17-19]. Several approaches have been used to explore the genetic underpinnings of phenotypic variation in the Glanville fritillary metapopulation, ranging from candidate gene approaches[13,20] to quantitative genetics[21,22], and whole-genome scans[23,24] , and have included both laboratory and natural environmental conditions.

The first *M. cinxia* genome assembly was released in 2014[25]. This genome was produced from a combination of 454 sequencing for contig assembly, followed by scaffolding with Illumina paired-end (PE), SOLiD mate-pair reads and PacBio data. The size of the final assembly was 390 Mb made up from 8,261 scaffolds, with a scaffold N50 of 119,328. Scaffolds were assigned to chromosomes based on a linkage map produced from RAD sequencing[25]. We recently assessed the actual genome size using a k-mer based approach on Illumina sequencing data and obtained estimates ranging from 488 to 494 Mbp (Supplementary File 5, (Kmer_analysis_for_genome_size.docx)). It was considered that a new genome, sequenced using longer PacBio reads, would result in a more complete assembly and better represent the repetitive areas of the genome.

Here, a new sequencing and assembly of the *M. cinxia* genome has been carried out using a pool of seven male butterflies from a single larval family collected from Sottunga, an island in an eastern part of the archipelago. Sequencing was conducted using the PacBio RSII sequencer. An initial assembly was created using FALCON[27,28] followed by polishing performed with Quiver[27]. A new linkage map was created and used to assign the assembled scaffolds to their correct positions and orientations within the 31 chromosomes. The scaffolds were then gap-filled producing a final assembly of 484 Mb with a scaffold N50 of 17,331,753

bp. The obtained genome size is well in line with the k-mer estimates. Gene prediction on the genome assembly was carried out using MAKER v 2.31.10[29] that was run iteratively using several independent training sets. Manual annotation was performed for 1,232 of the gene models. The genome assembly increases greatly in contiguity and completeness compared to the first genome (Table 1) with chromosomal superscaffold N50 values of 17,331,753 bp in the new genome compared to 119,328 bp in the version 1 genome.

The significant increase in assembly size warrants a further investigation of the composition of these added sequences. Initial observations of individual alignments from genome-to-genome alignment show many collapsed repeat regions in the version 1 genome which are mapped to multiple chromosomes in version 2.

Table 1. Assembly statistics were calculated for the *M. cinxia* v2 genome, *M. cinxia* v1 scaffolds, and *B. mori* using the assembly-stats program (https://zenodo.org/badge/latestdoi/20772/rjchallis/assembly-stats). Statistics for *H. melpomene* v2.5 and *P. napi* v1.1 were obtained from LepBase[65].

	***M. cinxia* Version 2**	***M. cinxia* Version 1 Scaffolds**	***Bombyx mori***	***Pieris napi* v1.1**
Length (bp)	484,462,241	389,907,520	460,334,017	349,759,982
N(%)	<0.00	7.42	0.10	22.47
Scaffold count	31	8,261	696	2,969
Longest scaffold (bp)	22,190,643	668,473	21,465,692	15,427,984
Scaffold N50 length (bp)	17,331,753	119,328	16,796.068	12,597,868
Scaffold N50 count (L50)	13	970	13	13
Contig Count	529	48,180	726	53,510
Contig N50 length (bp)	1,831,849	14,057	12,201,325	10,538
Contig N50 count (L50)	79	7,366	16	6,914

Methods

An overview of the processing pipeline for the work is shown in Figure 1.

Genomic samples and DNA extraction

Owing to the facultatively univoltine life cycle of the butterfly in Finland, experimental inbreeding of the species would have taken several years. Therefore, we chose to sample individuals from an island population, Sottunga, expected to harbour lower genetic diversity compared to less isolated populations. Sottunga is part of the Åland Islands archipelago in the northern Baltic Sea, and the population was introduced here in 1991 using individuals collected on the mainland of Åland Island[30]. This introduction was carried out with 71 larval families. The distance to the nearest *M. cinxia* population across the water is 5 km, and we therefore assume that the introduced population has been (almost) completely isolated ever since. Furthermore, the effective population size of *M. cinxia* in Sottunga has been very low during the last 24 years (on average 57 larval nests/year in 1993-2019), and it has experienced several strong bottlenecks[31]. Using genomic markers, Fountain et al.[17] demonstrated that samples from the Sottunga population separate clearly from samples collected on the mainland.

During the fall survey of 2014 (see Ojanen et al. for details of the survey[3]) we collected individuals from one larval group on the island of Sottunga (patch number 1439, Lat: 60.13628 Long: 20.66869). The larvae were collected once they were in diapause and most likely comprise full-sibs[18]. The larval group was kept in diapause (+5 °C) until the following spring and then reared to adulthood under common garden conditions (28:8°C; 12L:12D) at the Lammi Biological Station, University of Helsinki. After eclosion, butterflies were sexed and stored at -80°C. High-molecular-weight DNA was isolated from seven adult males using the caesium chloride (CsCl) method[25]. Several individuals and the selected

method were used to obtain enough starting material for constructing SMRT sequencing library.

SMRT sequencing libraries and sequencing

Library construction for Pacific Biosciences sequencing was carried out using the protocols recommended by the manufacturer (Pacific Biosciences, Menlo Park, CA, USA). Genomic DNA was sheared using a Megaruptor (Diagenode, Seraing, Belgium) followed by damage repair, end-repair, hairpin ligation, and size selection using BluePippin (Sage Science, Beverly, MA, USA). After primer annealing and polymerase binding, the DNA templates were sequenced on a PacBio RSII sequencer using P6/C4 chemistry and 360 min video time at the DNA Sequencing and Genomics Laboratory, Institute of Biotechnology, University of Helsinki, Finland[32].

Genome Assembly

The genome was assembled using the FALCON assembler (FALCON-Integrate-1.8.6)[26,27] with a read length cut-off of 18,000 bp. This cut-off was found to give the best contiguity for the assembly based on N50 value, while minimizing the percentage of possibly erroneous contigs. The erroneous contigs were detected by mapping markers of the linkage map from the previously published genome[25] to contigs, and calculating the percentage of chimeric contigs. We tested three different read length cut-offs 16,000 bp, 18,000 bp, and 20, 000 bp, all of which included approximately 9% of chimeric contigs. The assembly was based on 1,9M PacBio reads, 24,4 Gbp in total, with an N50 of 18,479 bp which is approximately 50x coverage based on the final genome size. With the selected read cut-off the data produced 10.8 Gb of corrected reads which were further assembled using the FALCON software. The assembly yielded 4,559 primary contigs containing 739.9 Mb with an N50 of 340 kb and 1,661 alternative contigs containing 118.1 Mb with an N50 of 85,246

bp. The alternative contigs were automatically separated by the FALCON pipeline. The data were also assembled using miniasm software (0.2-r137-dirty)[33] which yielded similar results. The larger than expected initial assembly size, approximately 1.5 times the k-mer estimate, is due to the multiple haplotypes originating from the 7 individuals used in sequencing.

To evaluate the putative chimeric contigs and assembly errors suggested by the genetic map, the raw SMRT sequencing data were mapped to the assembly primary contigs using the Burrows-Wheeler Aligner (BWA-0.7.17) with the MEM algorithm[34]. The alignments of the 425 regions discovered as possibly chimeric were visually inspected. Of these regions, 92 showed even read coverage and no evident signs of assembly errors, while 333 regions contained areas with low coverage and/or repeat regions indicated by high coverage that had led to erroneous overlaps and mis-assemblies. These errors were identified by positions where the majority of the reads did not fully align, i.e. the alignments ended mid-read. The assembly was split in the positions where the coverage was at minimum . The resulting assembly was polished using the SMRT sequencing data and Quiver[26] software from the SMRT Tools-package (PacBio).

Linkage Map

Linkage mapping was constructed from whole genome resequencing data of F2 crosses of *M. cinxia*. The grandparents of these F2 crosses are offspring of wild collected *M. cinxia* originating from two distantly related *M. cinxia* populations around the Baltic Sea; the Åland Islands (ÅL)[1] and Pieni Tytärsaari (PT) populations[35]. Between population crosses of type ÅL♂xPT♀ and ÅL♀xPT♂ were established to create the F1 population. Some of these F1 individuals were used to establish the F2 families, actively avoiding mating among siblings. A subset of the resulting full-sib families were reared to adulthood, and five of these

F2 families, together with their parents and grandparents, were selected for resequencing. In total, resequencing included ten grandparental individuals, ten F1 parents and 165 F2 individuals (N=185).

All the larvae from different generations completed development under common garden conditions (28:15°C; 12L:12D) utilizing fresh leaves of greenhouse grown *Veronica spicata*. Diapausing larvae were kept in a growth chamber at +5°C and 80% relative humidity for approximately seven months to mimic the normal wintertime conditions for these butterflies. Adults were kept in hanging cages (of 50 cm height and 40 cm diameter) at ~26:18°C; 9L:15, and fed *ad libitum* with 20% honey-water solution throughout the experiments.

Before DNA extraction the adult butterflies were stored at -80°C, and either thorax or abdomen tissue of these individuals was used for sequencing. Tissues were homogenized prior to extraction using TissueLyser (Qiagen, Venlo, The Netherlands) at 30/s for 1.5 mins with Tungsten Carbide Beads, 3 mm (Qiagen, Venlo, The Netherlands) and ATL buffer (Qiagen, Venlo, The Netherlands). DNA was extracted using the NucleoSpin 96 Tissue Core Kit (Macherey-Nagel) according to the manufacturer's protocol with the exception that lysing time was extended to overnight. The samples were additionally treated with RNase A (Thermo Scientific) before sequencing. Sequencing was performed using standard PE library preparation and Illumina Hi Seq 2000 with 125 bp paired-end reads.

The mapping procedure followed the Lep-MAP3[26] pipeline. First, individual fastq files were mapped to the contig assembly using BWA MEM (BWA-0.7.17) [34] and individual bam files were created using SAMtools (1.6)[36,37]. SAMtools mpileup and the scripts pileupParser2.awk and pileup2posterior.awk were used to obtain input data for Lep-MAP3. Then ParentCall2 (parameter: ZLimit=2) and Filtering2 (parameters: dataTolerance=0.0001; removeNonInformative=1; familyInformativeLimit=4) were run to obtain data with at least

four informative families for each marker, resulting in a final input with almost 2.5M markers.

SeparateChromosomes2 was run on the final data (parameters lodLimit=20; samplePair=0.2;numThreads=48) to obtain 31 linkage groups with a total of 2.4M markers. OrderMarkers2 was run (parameter recombination2=0) on each linkage group (chromosome). This map was used to anchor the contig assembly into chromosomes. To validate anchoring, the map construction was repeated in the same way except that OrderMarkers2 was run on the physical order of markers to reduce noise in the linkage map. Finally, the raw data were re-mapped to the gap-filled chromosome level assembly and the linkage map was re-done in the new physical order to infer final recombination rates.

Anchoring the genome and resolving haplotypes using the linkage map

The contigs were aligned against each other and lift-over chains were created by running the first two steps (batch A and B to calculate the alignment chain) of HaploMerger2[38] pipeline. By inspecting this chain, contigs fully contained in some longer contig were removed. Initial contig order and orientation within each chromosome was calculated by the median map position of each contig and the longest increasing subsequence of markers, respectively. For each chromosome, Marey map [39], a scatter plot of physical and linkage positions combining the genetic and physical maps, and contig-contig alignments from the chain were recorded. The contigs orders and orientations were manually fixed when needed if the map had support for alternative orientation or the contig-contig alignments were linking contigs together and any assembly errors that were found were corrected by splitting the contigs accordingly. Also, partially haplotypic contigs were found and collapsed based on the Marey maps and contig-contig alignments. This manual work resulted in the final reference genome sequence including start and end positions of contigs in the correct order and

orientation for each chromosome. Finally, the haplotype corrected genome was gap-filled using PBJelly software (PBSuite_15.8.24)[40] with the original SMRT sequencing data, and polished with the Quiver tool[26] from the SMRT Tools-package 2.3.0 (PacBio) and with Pilon (1.21)[41].

The chromosomes were aligned against the *Heliconius melpomene* (2.5)[42,43] and *Pieris napi*[44] genomes using the LAST aligner(938)[45] to check structural similarity between the species (Supplementary Figures S1-13). An overview alignment for *H. melpomene* was created using D-GENIES (1.2.0)[46] (Figure 2). The data show a high level of collinearity between *M. cinxia* and *H. melpomene* chromosomes, as described before in Ahola et al.[25]. A notably interesting point is the lack of collinearity with sex chromosomes (*M. cinxia* chromosome 1 & *H. melpomene* chromosome 21). Furthermore, the visible vertical lines show the effect of long read assembly on repeat resolution. With long reads spanning the repeats and allowing their accurate placement in the contigs, in *M. cinxia* the repeats are placed in single chromosomes whereas in *H. melpomene* they are present in all chromosomes.

Repeat masking and annotation

Genomic assemblies were masked with *de novo* repeat libraries by RepeatMasker v.4.0.9 (http://www.repeatmasker.org/). *De novo* repeat libraries were constructed from original PacBio reads with lengths over 30,000bp and assembled scaffolds (pseudo chromosomes) using RepeatModeler v 1.0.10 (http://www.repeatmasker.org/RepeatModeler/) and the LtrHarvest/LtrDigest-pipeline[47,48]. Repeat families were clustered using cd-hit-est applying 80/80-rule (80% identity over 80% length)[49]. Repeat annotations were confirmed by RepBase Release 20181026[50] and Dfam version 3.1[51].

Transcriptome assembly

In order to aid construction of gene models, we capitalised on two transcriptome assemblies which we produced as part of separate projects in our lab to be presented in upcoming publications ([5] Oostra et al. unpubl. data). Importantly for gene model construction, they represent a wide range of transcriptional diversity, as the RNAseq data are derived from various developmental stages (first instar larvae, fourth instar larvae, and adult thorax and abdomen). All individuals were lab-reared but originated from the same butterfly metapopulation. Transcriptome 1 was produced using a set of 78 individually sequenced female larvae (fourth developmental instar)[5], sequenced to an average depth of 17.3M reads (read lengths 85 bp and 65 bp for forward and reverse PE reads, respectively). As the two sexes are practically indistinguishable in the larval stages, the females were identified based on homozygosity across a set of 22 Z-chromosome specific SNP loci[5]. To remove Illumina adapter sequences, we trimmed raw reads using Trimmomatic (Trimmomatic-0.35)[52], and normalised using Trinity v2.6.5[53]. We then used two separate procedures to construct *de novo* transcriptome assemblies, Trinity (v2.6.5) and Velvet / Oases (1.2.10)[54]. Trinity was run with standard settings, whereas Velvet / Oases used a range of seven kmer sizes (21 bp to 71 bp), producing a separate assembly for each kmer size. We then combined the resulting assemblies, filtered the combined assembly using the EvidentialGene (tr2aacds.pl VERSION 2017.12.21)[55] pipeline, and removed contigs smaller than 200 bp or expressed at a low level (< 1 normalized counts per million), yielding the final assembly. Transcriptome 2 was constructed from a set of 12 adult females (thorax and abdomen, without ovaries) and 48 first instar larvae, as part of a separate gene expression study (Oostra et al. unpubl. data). RNA from these 60 individual samples was sequenced to an average depth of 16.6M reads (86/74 bp PE). The stranded RNA-seq libraries were made using Ovation® Universal RNA-Seq System (Nugen) with custom ribosomal RNA removal. The libraries were paired-end sequenced on a NextSeq 500 using the 150 bp kit (Illumina) at the DNA sequencing and

genomics laboratory Institute of Biotechnology University of Helsinki. We trimmed the reads using fastp (v0.20.0)[56], and used the HISAT2 (2.0.4) / StringTie (1.3.5) pipeline[57] to construct a genome-guided transcriptome assembly, mapping the RNAseq reads to the new genome assembly. Transcriptome 1 yielded 69,182 putative transcripts with average length of 727 bp (95% CI: 206 - 3433), while transcriptome 2 yielded 137,250 putative transcripts with average length of 1737 (95% CI: 203 - 9106). These statistics should be interpreted with caution, as the assemblies derive from different life stages, and different assembly and filtering approaches were used (reflecting differences in histories of the datasets as they were produced for different projects).

Gene model Annotation

Initial gene predictions were obtained by running the MAKER v 2.31.10[29] gene prediction programme in an iterative procedure. In the first round of MAKER, transcriptome assembly 1, described above, was provided as evidence, and genes were predicted solely from the aligned transcripts. This resulted in 14,738 gene models. These gene models were then used for training the SNAP (2013-02-16)[58] and AUGUSTUS (3.3.2)[59] gene predictors. A second round of MAKER was run providing the *de novo* transcripts from both transcriptomes (see previous paragraph), trained gene prediction models, repeat masking file and protein data from other lepidopteran species. The MAKER settings were adjusted to allow prediction of gene models without requiring a corresponding transcript in the *de novo* transcriptome assembly. Following each round of MAKER gene prediction, the annotation completeness was assessed using BUSCO[60,61].

Manual Annotation

Manual annotation was performed for 1,232 genes, using the Apollo collaborative annotation system version 2.1.0[62]. The collaborative annotation environment was set up in Ubuntu

Linux 14.04 server with 250 GB RAM and 48 AMD Opteron 6,168 processing cores. This was later upgraded to a cloud server provided by the Finnish IT Center for Science (CSC) and running on Ubuntu Linux 18.04 with 200 GB RAM and 40 Intel Xeon model 85 processing cores. Evidence tracks were produced containing gene predictions from three rounds of MAKER, RNASeq alignments of sequence reads and protein alignments from other species (Table 2). RNASeq alignments comprised a mixed tissue pooled sample, an abdomen pooled sample and six larval samples (from transcriptome 1) selected to represent a diverse range and included, for example, both sexes and different family backgrounds. A list of gene families that were considered of particular interest in butterfly research were identified for prioritisation during the manual annotation. (Supplementary File 4, Prioritized_gene_families.docx)). The gene annotators were able to select a family of genes for annotation or a random selection from the prioritized families was given. Gene models were corrected by examining the evidence tracks in the browser, conducting blast searches and examining multiple alignments of protein sequences. In total for the 1,232 genes, 1,455 mRNAs were manually inspected of which 814 genes and mRNAs were changed. Most changes were made to exon borders and mRNA exon structure, especially in the case of multiple isoforms.

Table 2. Evidence tracks that were used during the manual annotation of 1,232 *M. cinxia* genes

**Evidence track**	**Type**	**Description**
Maker 1	Gene prediction	Initial maker gene predictions based on EST alignments
Maker 2	Gene prediction	Second round of gene predictions from EST alignments, protein alignments and gene predictors

		trained on maker 1.
RNASeq abdomen pool	RNASeq alignment	RNASeq reads aligned to the genome with STAR[68]
RNASeq mixed tissue pool	RNASeq alignment	
B. mori proteins	Protein alignment	Proteins sequences aligned to the genome with AAT.
H. melpomene proteins	Protein alignment	
D. melanogaster proteins	Protein alignment	
H. erato proteins	Protein alignment	
RNASeq - female larvae family 80	RNASeq alignment	RNASeq reads aligned to the genome with STAR[68]
RNASeq - female larvae family 70	RNASeq alignment	
RNASeq female larvae family 119	RNASeq alignment	
RNASeq female larvae family 120	RNASeq alignment	
RNASeq male larvae family 80	RNASeq alignment	
RNASeq male larvae family 119	RNASeq alignment	

Final Gene Models

Following the manual annotation, the SNAP[58] and AUGUSTUS[59] gene predictors were retrained using the manually annotated gene models. MAKER was run using the updated gene predictors, transcriptome 1 and 2, and also using a masking file for repeats. As a final step to incorporate the manually annotated gene models, MAKER was run providing the

previous MAKER file to pred_gff and the manually annotated models to model_gff. Gene functional prediction was performed using Pannzer v2[63].

Ortholog identification

Predicted protein sequences from *Bombyx* mori[64] (January 2017 gene models), *P. napi*[44] and *H. melpomene* (Hmel2.5)[42,43] were downloaded from silkbase http://silkbase.ab.a.u-tokyo.ac.jp/cgi-bin/download.cgi, LepBase[65] and the Butterfly Genome Database http://butterflygenome.org respectively. OrthoFinder v2.3.3[66] was run to identify orthologs between *M. cinxia*, *B. mori, P napi* and *H. melpomene* using blast as the search tool (Figure 3 & Supplementary Figure S14).

Data Validation and quality control

To assess the quality of the assembly, assembly statistics were generated using assembly-stats[65] and compared to the v1 genome as well as the *H. melpomene*, *B. mori* and *P. napi* genome assemblies (Table 1). The new genome contains 94 Mb more sequence than the previous scaffold assembly. Based on the observations of individual alignments in the full genome alignment between the version 1 and version 2, there are many regions in the genome 1 which are aligned into multiple positions in version 2. This points to collapsed repeat regions in version 1 and more accurate repeat placement due to the long read sequencing in version 2. The N50 length and L50 value at scaffold or chromosome level have improved greatly compared to the previous genome. To check for possible duplication or missing areas in the assembly, an assessment was made of the completeness of single copy orthologs from BUSCO[60,61] eukaryota, arthropoda and metazoa gene sets (Table 3). In each of the gene sets, 93.4-94.9% of the expected single copy orthologs were found in complete and single copies. The duplication rate was estimated to be between 1.4 and 2.3%. A total of 1,232 gene models were manually curated using the Apollo annotation system[62] to ensure the quality

of the models. To test for contamination, the predicted protein sequences were checked with AAI-profiler[67] to identify sequences originating from different taxa (Supplementary Files 1-3 (AAI.html, matrix.html, krona.html)). Overall, 42% of the genome was composed of repeat sequences (Figure 4 and Supplementary Figures S15-20 (chromosome specific repeat classes)). There were no clear differences in the repeat contents between chromosomes (Supplementary Table 1) which further supports the more accurate placement of repeats due to the long read sequencing in version 2. Long interspersed elements (LINE) were the most prevalent.

Table 3. BUSCO completeness estimates of the v2 genome based on the eukaryota, arthropoda and metazoa gene sets.

Lineage	BUSCO Category				
	Complete	Single-copy	Duplicated	Fragmented	Missing
Eukaryota	290	283	7	5	8
	95.7%	93.4%	2.3%	1.7%	2.6%
Arthropoda	1027	1012	15	7	32
	96.3%	94.9%	1.4%	0.7%	3.0%
Metazoa	935	921	14	12	31
	95.6%	94.2%	1.4%	1.2%	3.2%

Re-use potential

The substantial improvements in contiguity and gene annotation quality of the new genome will enable a range of important new studies and open up possibilities for future work. The results also demonstrate that with the use of proper computational tools and data, it is

possible to obtain a high quality, chromosome scale reference genome even when a single individual organism will not provide enough high molecular weight DNA for long read sequencing. Furthermore, we show the potential of the linkage mapping: it anchors contigs to actual chromosomes, instead of just linking different contigs together as is done for example in Hi-C approach. Moreover, the haplotype problem is not tackled by Hi-C. Our high-density linkage map allows us to put basically all contigs into chromosomes. It is worth noting that the linkage map is not scaffolding directly but it will put contigs into map positions. Scaffolding is possible if a contig spans two or more map positions. Otherwise the contig can be placed only partially. In addition to the linkage map approach, we used extensive manual curation of the assembly to avoid chimeric parts and improve the assembly quality. Current research aims at identifying mechanisms underlying key life history adaptations, exploring the extent of natural variation and selection on these adaptations in wild populations, and integrating these insights with the exceptional ecological, demographic, and climatic data available for this system. Future studies in this direction will help understand the mechanisms maintaining variation in life-histories across spatial and temporal scales, and on the extent to which phenotypic variation in these and other traits may contribute to a population's adaptive capacity under climate change. Several studies in different species illustrate how stress responses can be crucial for survival under variable environments, both within and between generations. The Glanville fritillary is being used to explore how environmental information is translated into adaptive phenotypic changes, and how these responses are transmitted to future generations, using transcriptomic and epigenetic approaches. Such studies will greatly benefit from an improved annotation permitting exon-specific expression quantification, and identification of epigenetic marks and other functional variants outside coding regions. Exploiting current and past large-scale sampling efforts, these new studies apply population genomic approaches that are greatly facilitated by the increased assembly contiguity, for

instance by permitting linkage disequilibrium (LD) and haplotype-based selection analyses. Other avenues of research enabled by the improved genome assembly include structural variation, regulatory evolution, recombination rate variation, and coalescent-based demographic analyses. The increasing availability of chromosome-level lepidopteran genomes such as ours permits exciting new comparative phylogenetic analyses, for example of chromosome and genome evolution.

Availability of source code and requirements (if used in the paper)

Not applicable

Availability of supporting data

The SMRT sequencing reads used for the genome assembly have been deposited to the sequence read archive under Bioproject PRJNA607899 accession number SRR11184190.

The genome has been deposited to GenBank under Bioproject PRJNA607899

The Illumina reads used for the linkage map have been deposited to the sequence read archive under Bioproject PRJNA608928 accession numbers SRR11186917- SRR11187107.

Transcriptome 1 RNASeq reads have been deposited to NCBI GEO under accession number GSE159376

Transcriptome 2 RNASeq reads have been deposited to NCBI SRA under ioproject PRJNA670126

Declarations

List of abbreviations

Not applicable

Ethics approval and consent to participate

There are no ethical policies related to working with insect data. The Glanville fritillary is not considered endangered in the Åland islands and no permits are required for sampling. However, we note that within this project the larval sampling for genetic analyses is done non-invasively in the field. ensuring insignificant demographic impact. In addition, as the sampling will take place prior diapause (Åland) when mortality is generally the highest – the collection has negligible effect on the family survival or the demography of populations.

Consent for publication

Not Applicable

Competing interests

'The authors declare that they have no competing interests'.

Funding

Funding for M.S, D.B, V.O, E.vB, J.T & A.K was provided by a grant from the European Research Council (Independent Starting Grant No. 637412 'META-STRESS' to MS) and J Ö-U, V.A and D.B from the Academy of Finland grant (Decision No. 304041 to MS & Decision No. 283108 to Ilkka Hanski). A.D was funded by a Marie Sklodowska Curie Individual Fellowship (#790531, Host Sweet Home). O-P.S. was supported by the "TTÜ development program 2016– 2022", project code 2014-2020.4.01.16-0032.

Authors' contributions

O-P.S assembled the genome, processed the chimeric contigs, performed the gap filling and the polishing of the assembly, and participated in the genome analysis.

V.A was responsible for the initial idea of the approach for the genome related activities, coordinated the first part of the project, designed and produced data for the linkage map, and worked on solving the haplotypes from the initial assembly.

D.B performed gene prediction, functional annotation, ortholog prediction, and managed the manual annotation.

J.K installed and managed the Apollo annotation server.

S.I was responsible for larval rearing and preparation of butterfly crosses.

P.R performed the linkage mapping and anchored the genome onto chromosomes.

V.O assembled the transcriptomes used for gene prediction.

Lo.P manually inspected the chimeric contigs.

A.R performed DNA extraction.

D.B, J.K, V.O, T.D, M.F.D, A.D, I.C.D, P.H, A.K, S.S.K, S.O.K, E.L, S.L, J.M, A.N, M.C-M, V.P, T.S, A.I.T, V.T, E.vB, J.Ö-U and M.S participated in manual annotation.

J.T performed the annotation of transposable elements and repeat classes.

L.P was responsible for the management of the DNA sequencing.

M.J.F was responsible for the management of the genome analysis.

P.A was responsible for the initial idea of the approach for the genome related activities, and the management of the genome analysis.

M.S was responsible for the management of the *M. cinxia* database and genome analysis.

O-P.S, D.B, V.A, P.R, J.T, J.K, V.O, L.P, M.J.F, P.A and M.S wrote the manuscript.

Acknowledgements

The authors wish to acknowledge CSC – IT Center for Science, Finland, for computational resources. We thank Torsti Schulz and Emily Hornett for annotating > 10 genes. We thank the personnel of the DNA sequencing and genomics laboratory (Institute of Biotechnology, Helsinki, Finland) for performing the NGS sequencing.

Authors' information

[1.] Institute of Biotechnology, University of Helsinki, Finland

[2.] Organismal and Evolutionary Biology Research Programme, University of Helsinki, Finland

[3.] Department of Chemistry and Biotechnology, Tallinn University of Technology, Estonia

[4.] Ming Wai Lau Centre for Reparative Medicine, Karolinska Institutet, Hong Kong

[5.] Natural Resource Institute (LUKE), Finland

[6.] Department of Evolution, Ecology and Behaviour, University of Liverpool, UK

[7.] Department of Biological Sciences, Louisiana State University, USA

[8.] Department of Biology, Lund University, Sweden

[9.] Department of Environmental and Biological Sciences, University of Eastern Finland, Finland

[10.] Department of Entomology and Nematology, University of Florida, USA

[11.] Department of Zoology, Loknete Vyankatrao Hiray Arts, Science & Commerce College, India

[12.] Swiss Federal Institute of Aquatic Science and Technology, Department of Aquatic Ecology, Switzerland

[13.] College of Plant Protection, China Agricultural University, China

[14.] Department of Zoology, Stockholm University, Sweden

[15.] Viikki Plant Science Centre, Organismal and Evolutionary Biology Research Programme, University of Helsinki, Finland

[16.] Helsinki Institute of Life Science (HiLIFE), University of Helsinki, Finland

Present address:

Lo.P - School of Life Sciences, University of Warwick, UK

V.T - Luomus, Finnish Museum of Natural History, Zoology Unit, University of Helsinki, Helsinki, Finland

S.O.K, P.H and J.M - Biosafe – Biological Safety Solutions Ltd, Microkatu 1 M, 70210 Kuopio, Finland

corresponding author(s): marjo.saastamoinen@helsinki.fi, olli-pekka.smolander@taltech.ee

Endnotes

Not applicable

Figure 1. An overview of the assembly and annotation process of the improved Glanville fritillary genome.

Figure 2. A dot-plot structural comparison of the *H. melpomene* genome against the *M. cinxia* v2 genome.

The alignment was created using D-GENIES (1.2.0)[46]. The diagonal lines indicate the collinearity between the two species. The lack of collinearity in sex chromosomes is visible in the upper left corner between Mcnxia_v2 chr 01 and Hmel2.5 chr 21. The visible vertical lines show repeats that are resolved in Mcinxia_v2 but are present in all chromosomes in Hmel2.5_chr.

Figure 3. A circos plot showing the orthologs between *M. cinxia* and *H. melpomene*

Orthologs between *M. cinxia* and *H. melpomene* were identifies using OrthoFinder and filtered for one-to-one orthologs. The internal links in the circos plot indicate the orthologs between *M. cinxia* and *H. melpomene*. The links are coloured according to the *M. cinxia* chromosome.

Figure 4. Relative amounts of different repeat classes in *M. cinxia* genome

Repeat classes and coverage of the *M. cinxia* genome v2: DNA = classII; LINE = Long interspersed elements; LTR = Long terminal repeats; LOW_COMPLEXITY = Low complexity repeated DNA; RC = Rolling circle elements (e.g. Helitrons); SINE = Short interspersed elements; Satellite = Satellite DNA; SIMPLE_REPEAT = Simple repeated motifs; EXON = Exonic regions; UNCOVERED = rest of the chromosomes.

References

1 Hanski, I. Habitat connectivity, habitat continuity, and metapopulations in dynamic landscapes. *Oikos* **87**, 209-219, doi:Doi 10.2307/3546736 (1999).

2 Ovaskainen, O. & Saastamoinen, M. Frontiers in Metapopulation Biology: The Legacy of Ilkka Hanski. *Annu Rev Ecol Evol S* **49**, 231-252, doi:10.1146/annurev-ecolsys-110617-062519 (2018).

3 Ojanen, S. P., Nieminen, M., Meyke, E., Pöyry, J. & Hanski, I. Long-term metapopulation study of the Glanville fritillary butterfly (*Melitaea cinxia*): survey methods, data management, and long-term population trends. *Ecol Evol* **3**, 3713-3737, doi:10.1002/ece3.733 (2013).

4 Saastamoinen, M., Hirai, N. & van Nouhuys, S. Direct and trans-generational responses to food deprivation during development in the Glanville fritillary butterfly. *Oecologia* **171**, 93-104, doi:10.1007/s00442-012-2412-y (2013).

5 Kahilainen, A., Oostra, V., Somervuo, P., Minard, G., & Saastamoinen, M. Alternative developmental and transcriptomic responses to host plant water limitation in a butterfly metapopulation. bioRxiv, 2021.02.24.432453 (2021).

6 Hanski, I., Saastamoinen, M. & Ovaskainen, O. Dispersal-related life-history trade-offs in a butterfly metapopulation. *J Anim Ecol* **75**, 91-100, doi:10.1111/j.1365-2656.2005.01024.x (2006).

7 Niitepõld, K. *et al.* Flight metabolic rate and Pgi genotype influence butterfly dispersal rate in the field. *Ecology* **90**, 2223-2232, doi:Doi 10.1890/08-1498.1 (2009).

8 Hanski, I. & Singer, M. C. Extinction-colonization dynamics and host-plant choice in butterfly metapopulations. *Am Nat* **158**, 341-353, doi:Doi 10.1086/321985 (2001).

9 Rosa, E., Woestmann, L., Biere, A. & Saastamoinen, M. A plant pathogen modulates the effects of secondary metabolites on the performance and immune function of an insect herbivore. *Oikos* **127**, 1539-1549, doi:10.1111/oik.05437 (2018).

10 Rosa, E., Minard, G., Lindholm, J. & Saastamoinen, M. Moderate plant water stress improves larval development, and impacts immunity and gut microbiota of a specialist herbivore. *Plos One* **14**, doi:ARTN e020429210.1371/journal.pone.0204292 (2019).

11 Salgado, A. L. & Saastamoinen, M. Developmental stage-dependent response and preference for host plant quality in an insect herbivore. *Anim Behav* **150**, 27-38, doi:10.1016/j.anbehav.2019.01.018 (2019).

12 Van Nouhuys, S. & Lei, G. C. Parasitoid-host metapopulation dynamics: the causes and consequences of phenological asynchrony. *J Anim Ecol* **73**, 526-535, doi:DOI 10.1111/j.0021-8790.2004.00827.x (2004).

13 de Jong, M. A. & Saastamoinen, M. Environmental and genetic control of cold tolerance in the Glanville fritillary butterfly. *J Evolution Biol* **31**, 636-645, doi:10.1111/jeb.13247 (2018).

14 Saastamoinen, M., Ikonen, S., Wong, S. C., Lehtonen, R. & Hanski, I. Plastic larval development in a butterfly has complex environmental and genetic causes and consequences for population dynamics. *J Anim Ecol* **82**, 529-539, doi:10.1111/1365-2656.12034 (2013).

15 Niitepõld, K. & Saastamoinen, M. A candidate gene in an ecological model species: Phosphoglucose isomerase (Pgi) in the Glanville fritillary butterfly (*Melitaea cinxia*). *Ann Zool Fenn* **54**, 259-273, doi:Doi 10.5735/086.054.0122 (2017).

16 de Jong, M. A., Wong, S. C., Lehtonen, R. & Hanski, I. Cytochrome P450 gene CYP337 and heritability of fitness traits in the Glanville fritillary butterfly. *Mol Ecol* **23**, 1994-2005, doi:10.1111/mec.12697 (2014).

17 Fountain, T. *et al.* Predictable allele frequency changes due to habitat fragmentation in the Glanville fritillary butterfly (vol 113, pg 2678, 2016). *P Natl Acad Sci USA* **113**, E5363-E5363, doi:10.1073/pnas.1613041113 (2016).

18 Fountain, T. *et al.* Inferring dispersal across a fragmented landscape using reconstructed families in the Glanville fritillary butterfly. *Evol Appl* **11**, 287-297, doi:10.1111/eva.12552 (2018).

19 Dileo, M. F., Husby, A. & Saastamoinen, M. Landscape permeability and individual variation in a dispersal-linked gene jointly determine genetic structure in the Glanville fritillary butterfly. *Evol Lett* **2**, 544-556, doi:10.1002/evl3.90 (2018).

20 Haag, C. R., Saastamoinen, M., Marden, J. H. & Hanski, I. A candidate locus for variation in dispersal rate in a butterfly metapopulation. *P Roy Soc B-Biol Sci* **272**, 2449-2456, doi:10.1098/rspb.2005.3235 (2005).

21 Mattila, A. L. K. & Hanski, I. Heritability of flight and resting metabolic rates in the Glanville fritillary butterfly. *J Evolution Biol* **27**, 1733-1743, doi:10.1111/jeb.12426 (2014).

22 Klemme, I. & Hanski, I. Heritability of and strong single gene (Pgi) effects on life-history traits in the Glanville fritillary butterfly. *J Evolution Biol* **22**, 1944-1953, doi:10.1111/j.1420-9101.2009.01807.x (2009).

23 Kvist, J. *et al.* Flight-induced changes in gene expression in the Glanville fritillary butterfly. *Mol Ecol* **24**, 4886-4900, doi:10.1111/mec.13359 (2015).

24 Kvist, J. *et al.* Temperature treatments during larval development reveal extensive heritable and plastic variation in gene expression and life history traits. *Mol Ecol* **22**, 602-619, doi:10.1111/j.1365-294X.2012.05521.x (2013).

25 Ahola, V. *et al.* The Glanville fritillary genome retains an ancient karyotype and reveals selective chromosomal fusions in Lepidoptera. *Nat Commun* **5**, doi:ARTN 473710.1038/ncomms5737 (2014).

26 Rastas, P., Paulin, L., Hanski, I., Lehtonen, R. & Auvinen, P. Lep-MAP: fast and accurate linkage map construction for large SNP datasets. *Bioinformatics* **29**, 3128-3134, doi:10.1093/bioinformatics/btt563 (2013).

27 Chin, C. S. *et al.* Nonhybrid, finished microbial genome assemblies from long-read SMRT sequencing data. *Nat Methods* **10**, 563-+, doi:10.1038/Nmeth.2474 (2013).

28 Chin, C. S. *et al.* Phased diploid genome assembly with single-molecule real-time sequencing. *Nat Methods* **13**, 1050-+, doi:10.1038/Nmeth.4035 (2016).

29 Campbell, M. S., Holt, C., Moore, B. & Yandell, M. Genome Annotation and Curation Using MAKER and MAKER-P. *Curr Protoc Bioinformatics* **48**, 4.11.11-39 (2014).

30 Thomas, C. D. & Hanski, I. in *Ecology, genetics and evolution of metapopulations* 489-514 (Elsevier, 2004).

31 van Bergen, E. *et al.* Summer drought decreases the predictability of local extinctions in a butterfly metapopulation. Preprint at https://doi.org/10.1101/863795 (2019).

32 Salojärvi, J. *et al.* Genome sequencing and population genomic analyses provide insights into the adaptive landscape of silver birch. *Nat Genet* **49**, 904-912 (2017).

33 Li, H. Minimap and miniasm: fast mapping and de novo assembly for noisy long sequences. *Bioinformatics* **32**, 2103-2110, doi:10.1093/bioinformatics/btw152 (2016).

34 Li, H. Aligning sequence reads, clone sequences and assembly contigs with BWA-MEM. Preprint at *arXiv:1303.3997* (2013).

35 Mattila, A. L. K. *et al.* High genetic load in an old isolated butterfly population. *P Natl Acad Sci USA* **109**, E2496-E2505, doi:10.1073/pnas.1205789109 (2012).

36 Li, H. A statistical framework for SNP calling, mutation discovery, association mapping and population genetical parameter estimation from sequencing data. *Bioinformatics (Oxford, England)* **27**, 2987-2993 (2011).

37 Li, H. *et al.* The Sequence Alignment/Map format and SAMtools. *Bioinformatics (Oxford, England)* **25**, 2078-2079 (2009).

38 Huang, S., Kang, M. & Xu, A. HaploMerger2: rebuilding both haploid sub-assemblies from high-heterozygosity diploid genome assembly. *Bioinformatics (Oxford, England)* **33**, 2577-2579 (2017).

39 Chakravarti, A. A graphical representation of genetic and physical maps: the Marey map. Genomics. 11, 219-22. doi: 10.1016/0888-7543(91)90123-v (1991).

40 English, A. C. *et al.* Mind the Gap: Upgrading Genomes with Pacific Biosciences RS Long-Read Sequencing Technology. *Plos One* **7**, doi:ARTN e4776810.1371/journal.pone.0047768 (2012).

41 Walker, B. J. *et al.* Pilon: An Integrated Tool for Comprehensive Microbial Variant Detection and Genome Assembly Improvement. *Plos One* **9**, doi:ARTN e11296310.1371/journal.pone.0112963 (2014).

42 Davey, J. W. *et al.* No evidence for maintenance of a sympatric Heliconius species barrier by chromosomal inversions. *Evol Lett* **1**, 138-154, doi:10.1002/evl3.12 (2017).

43 Davey, J. W. *et al.* Major Improvements to the *Heliconius melpomene* Genome Assembly Used to Confirm 10 Chromosome Fusion Events in 6 Million Years of Butterfly Evolution. *G3-Genes Genom Genet* **6**, 695-708, doi:10.1534/g3.115.023655 (2016).

44 Hill, J. *et al.* Unprecedented reorganization of holocentric chromosomes provides insights into the enigma of lepidopteran chromosome evolution. *Sci Adv* **5**, doi:ARTN eaau3648 10.1126/sciadv.aau3648 (2019).

45 Kielbasa, S. M., Wan, R., Sato, K., Horton, P. & Frith, M. C. Adaptive seeds tame genomic sequence comparison. *Genome Res* **21**, 487-493, doi:10.1101/gr.113985.110 (2011).

46 Cabanettes, F. & Klopp, C. D-GENIES: dot plot large genomes in an interactive, efficient and simple way. *Peerj* **6**, doi:ARTN e495810.7717/peerj.4958 (2018).

47 Ellinghaus, D., Kurtz, S. & Willhoeft, U. LTRharvest, an efficient and flexible software for de novo detection of LTR retrotransposons. *BMC Bioinformatics* **9**, doi:Artn 1810.1186/1471-2105-9-18 (2008).

48 Steinbiss, S., Willhoeft, U., Gremme, G. & Kurtz, S. Fine-grained annotation and classification of de novo predicted LTR retrotransposons. *Nucleic Acids Res* **37**, 7002-7013, doi:10.1093/nar/gkp759 (2009).

49 Fu, L. M., Niu, B. F., Zhu, Z. W., Wu, S. T. & Li, W. Z. CD-HIT: accelerated for clustering the next-generation sequencing data. *Bioinformatics* **28**, 3150-3152, doi:10.1093/bioinformatics/bts565 (2012).

50 Jurka, J. Repbase Update - a database and an electronic journal of repetitive elements. *Trends Genet* **16**, 418-420, doi:Doi 10.1016/S0168-9525(00)02093-X (2000).

51 Hubley, R. *et al.* The Dfam database of repetitive DNA families. *Nucleic Acids Res* **44**, D81-D89, doi:10.1093/nar/gkv1272 (2016).

52 Bolger, A. M., Lohse, M. & Usadel, B. Trimmomatic: a flexible trimmer for Illumina sequence data. *Bioinformatics (Oxford, England)* **30**, 2114-2120 (2014).

53 Grabherr, M. G. *et al.* Full-length transcriptome assembly from RNA-Seq data without a reference genome. *Nat Biotechnol* **29**, 644-652 (2011).

54 Schulz, M. H., Zerbino, D. R., Vingron, M. & Birney, E. Oases: robust de novo RNA-seq assembly across the dynamic range of expression levels. *Bioinformatics (Oxford, England)* **28**, 1086-1092 (2012).

55 Gilbert, D. Gene-omes built from mRNA-seq not genome DNA. (2013).

56 Chen, S., Zhou, Y., Chen, Y. & Gu, J. fastp: an ultra-fast all-in-one FASTQ preprocessor. *Bioinformatics (Oxford, England)* **34**, i884-i890 (2018).

57 Pertea, M., Kim, D., Pertea, G. M., Leek, J. T. & Salzberg, S. L. Transcript-level expression analysis of RNA-seq experiments with HISAT, StringTie and Ballgown. *Nat Protoc* **11**, 1650-1667 (2016).

58 Korf, I. Gene finding in novel genomes. *BMC Bioinformatics* **5**, 59 (2004).

59 Lomsadze, A., Ter-Hovhannisyan, V., Chernoff, Y. O. & Borodovsky, M. Gene identification in novel eukaryotic genomes by self-training algorithm. *Nucleic Acids Res* **33**, 6494-6506 (2005).

60 Simao, F. A., Waterhouse, R. M., Ioannidis, P., Kriventseva, E. V. & Zdobnov, E. M. BUSCO: assessing genome assembly and annotation completeness with single-copy orthologs. *Bioinformatics* **31**, 3210-3212, doi:10.1093/bioinformatics/btv351 (2015).

61 Waterhouse, R. M. *et al.* BUSCO Applications from Quality Assessments to Gene Prediction and Phylogenomics. *Mol Biol Evol* **35**, 543-548, doi:10.1093/molbev/msx319 (2018).

62 Dunn, N. A. *et al.* Apollo: Democratizing genome annotation. *PLoS Comput Biol* **15**, doi:ARTN e100679010.1371/journal.pcbi.1006790 (2019).

63 Törönen, P., Medlar, A. & Holm, L. PANNZER2: a rapid functional annotation web server. *Nucleic Acids Res* **46**, W84-W88, doi:10.1093/nar/gky350 (2018).

64 Kawamoto, M. *et al.* High-quality genome assembly of the silkworm, *Bombyx mori*. *Insect Biochem Mol Biol* **107**, 53-62, doi:10.1016/j.ibmb.2019.02.002 (2019).

65 Challis, R. J., Kumar, S., Dasmahapatra, K. K., Jiggins, C. D. & Blaxter, M. Lepbase: the Lepidopteran genome database. Preprint at https://www.biorxiv.org/content/10.1101/056994v1.abstract doi:10.1101/056994 (2016).

66 Emms, D. M. & Kelly, S. OrthoFinder: phylogenetic orthology inference for comparative genomics. *Genome Biol* **20**, doi:ARTN 23810.1186/s13059-019-1832-y (2019).

67 Medlar, A. J., Törönen, P. & Holm, L. AAI-profiler: fast proteome-wide exploratory analysis reveals taxonomic identity, misclassification and contamination. *Nucleic Acids Res* **46**, W479-W485, doi:10.1093/nar/gky359 (2018).

68 Dobin, A. *et al.* STAR: ultrafast universal RNA-seq aligner. *Bioinformatics* **29**, 15-21, doi:10.1093/bioinformatics/bts635 (2013).

Manuscript
Click here to access/download;Manuscript;Cinxia_genome_manuscript_giga

Title page

Improved chromosome-level genome assembly of the Glanville fritillary butterfly (Melitaea cinxia) integrating PacBio long reads and a high-density linkage map.

Olli-Pekka Smolander*[1,3], Daniel Blande*[2], Virpi Ahola[2,4], Pasi Rastas[1], Jaakko Tanskanen[5], Juhana I. Kammonen[1], Vicencio Oostra[2,6], Lorenzo Pellegrini[1], Suvi Ikonen[2], Tad Dallas[7], Michelle F. DiLeo[2], Anne Duplouy[2,8], Ilhan Cem Duru[1], Pauliina Halimaa[9], Aapo Kahilainen[2], Suyog S. Kuwar[10,11], Sirpa O. Kärenlampi[9], Elvira Lafuente[12], Shiqi Luo[13], Jenny Makkonen[9], Abhilash Nair[2], Maria de la Paz Celorio-Mancera[14], Ville Pennanen[15], Annukka Ruokolainen[2], Tarja Sundell[1], Arja I. Tervahauta[9], Victoria Twort[8], Erik van Bergen[2], Janina Österman-Udd[2], Lars Paulin[1], Mikko J. Frilander[#1], Petri Auvinen[#1], Marjo Saastamoinen[#2,16]

* These authors contributed equally to the work

# These authors contributed equally to the work

Abstract

The Glanville fritillary (*Melitaea cinxia*) butterfly is a long-term model system for metapopulation dynamics research in fragmented landscapes. Here, we provide a chromosome level assembly of the butterfly's genome produced from Pacific Biosciences sequencing of a pool of males, combined with a linkage map from population crosses. The final assembly size of 484 Mb is an increase of 94 Mb on the previously published genome. Estimation of the completeness of the genome with Benchmarking Universal Single-Copy Orthologs (BUSCO), indicates that the genome contains 93 - 95% of the BUSCO genes in

complete and single copies. We predicted 14,830 gene models using the MAKER pipeline and manually curated 1,232 of these gene models. The genome and its annotated gene models are a valuable resource for future comparative genomics, molecular biology, transcriptome and genetics studies on this species.

Keywords

Melitaea cinxia, Glanville fritillary, Genome, Spatial Ecology

Data Description

Context

Identifying and characterizing genes underlying ecologically and evolutionarily relevant phenotypes in natural populations has become possible with novel genomic tools that can also be utilized in 'non-model' organisms. The Glanville fritillary (*Melitaea cinxia*) butterfly, and in particular its metapopulation in the Åland Islands (SW Finland), is an ecological model system in spatial ecology[1,2]. In short, within this archipelago, the species inhabits a network of dry outcrop meadows and pastures, and persists as a classic metapopulation with high turnover in patch occupancy[1]. The network of 4,500 potential habitat patches has been systematically surveyed bi-annually for butterfly occupancy and abundance since 1993[3], providing a vast amount of ecological data on population dynamics[2]. Experimental manipulations under more controlled conditions are also possible due to the small size, high fecundity and relatively short generation time of the species. Consequently, the ecological understanding of the species expands to the life history ecology across development stages[4,5], dispersal dynamics[6,7], species interactions (host plants and parasitoids)[8-12],

and stress tolerance[13,14]. During the last decade, the system has also been used to study genetic and evolutionary processes, such as identifying candidate genes underlying variation and evolution of dispersal in fragmented habitats[15] and host plant preference[16], and assessing allelic variation and their dynamics in space and time with SNP data across populations[17-19]. Several approaches have been used to explore the genetic underpinnings of phenotypic variation in the Glanville fritillary metapopulation, ranging from candidate gene approaches[13,20] to quantitative genetics[21,22], and whole-genome scans[23,24] , and have included both laboratory and natural environmental conditions.

The first *M. cinxia* genome assembly was released in 2014[25]. This genome was produced from a combination of 454 sequencing for contig assembly, followed by scaffolding with Illumina paired-end (PE), SOLiD mate-pair reads and PacBio data. The size of the final assembly was 390 Mb made up from 8,261 scaffolds, with a scaffold N50 of 119,328. Scaffolds were assigned to chromosomes based on a linkage map produced from RAD sequencing[25]. We recently assessed the actual genome size using a k-mer based approach on Illumina sequencing data and obtained estimates ranging from 488 to 494 Mbp (Supplementary File 5, (Kmer_analysis_for_genome_size.docx)). It was considered that a new genome, sequenced using longer PacBio reads, would result in a more complete assembly and better represent the repetitive areas of the genome.

Here, a new sequencing and assembly of the *M. cinxia* genome has been carried out using a pool of seven male butterflies from a single larval family collected from Sottunga, an island in an eastern part of the archipelago. Sequencing was conducted using the PacBio RSII sequencer. An initial assembly was created using FALCON[27,28] followed by polishing performed with Quiver[27]. A new linkage map was created and used to assign the assembled scaffolds to their correct positions and orientations within the 31 chromosomes. The scaffolds were then gap-filled producing a final assembly of 484 Mb with a scaffold N50 of 17,331,753

bp. The obtained genome size is well in line with the k-mer estimates. Gene prediction on the genome assembly was carried out using MAKER v 2.31.10[29] that was run iteratively using several independent training sets. Manual annotation was performed for 1,232 of the gene models. The genome assembly increases greatly in contiguity and completeness compared to the first genome (Table 1) with chromosomal superscaffold N50 values of 17,331,753 bp in the new genome compared to 119,328 bp in the version 1 genome.

The significant increase in assembly size warrants a further investigation of the composition of these added sequences. Initial observations of individual alignments from genome-to-genome alignment show many collapsed repeat regions in the version 1 genome which are mapped to multiple chromosomes in version 2.

Table 1. Assembly statistics were calculated for the *M. cinxia* v2 genome, *M. cinxia* v1 scaffolds, and *B. mori* using the assembly-stats program (https://zenodo.org/badge/latestdoi/20772/rjchallis/assembly-stats). Statistics for *H. melpomene* v2.5 and *P. napi* v1.1 were obtained from LepBase[65].

	***M. cinxia* Version 2**	***M. cinxia* Version 1 Scaffolds**	***Bombyx mori***	***Pieris napi* v1.1**
Length (bp)	484,462,241	389,907,520	460,334,017	349,759,982
N(%)	<0.00	7.42	0.10	22.47
Scaffold count	31	8,261	696	2,969
Longest scaffold (bp)	22,190,643	668,473	21,465,692	15,427,984
Scaffold N50 length (bp)	17,331,753	119,328	16,796.068	12,597,868
Scaffold N50 count (L50)	13	970	13	13
Contig Count	529	48,180	726	53,510
Contig N50 length (bp)	1,831,849	14,057	12,201,325	10,538
Contig N50 count (L50)	79	7,366	16	6,914

Methods

An overview of the processing pipeline for the work is shown in Figure 1.

Genomic samples and DNA extraction

Owing to the facultatively univoltine life cycle of the butterfly in Finland, experimental inbreeding of the species would have taken several years. Therefore, we chose to sample individuals from an island population, Sottunga, expected to harbour lower genetic diversity compared to less isolated populations. Sottunga is part of the Åland Islands archipelago in the northern Baltic Sea, and the population was introduced here in 1991 using individuals collected on the mainland of Åland Island[30]. This introduction was carried out with 71 larval families. The distance to the nearest *M. cinxia* population across the water is 5 km, and we therefore assume that the introduced population has been (almost) completely isolated ever since. Furthermore, the effective population size of *M. cinxia* in Sottunga has been very low during the last 24 years (on average 57 larval nests/year in 1993-2019), and it has experienced several strong bottlenecks[31]. Using genomic markers, Fountain et al.[17] demonstrated that samples from the Sottunga population separate clearly from samples collected on the mainland.

During the fall survey of 2014 (see Ojanen et al. for details of the survey[3]) we collected individuals from one larval group on the island of Sottunga (patch number 1439, Lat: 60.13628 Long: 20.66869). The larvae were collected once they were in diapause and most likely comprise full-sibs[18]. The larval group was kept in diapause (+5 °C) until the following spring and then reared to adulthood under common garden conditions (28:8°C; 12L:12D) at the Lammi Biological Station, University of Helsinki. After eclosion, butterflies were sexed and stored at -80°C. High-molecular-weight DNA was isolated from seven adult males using the caesium chloride (CsCl) method[25]. Several individuals and the selected

method were used to obtain enough starting material for constructing SMRT sequencing library.

SMRT sequencing libraries and sequencing

Library construction for Pacific Biosciences sequencing was carried out using the protocols recommended by the manufacturer (Pacific Biosciences, Menlo Park, CA, USA). Genomic DNA was sheared using a Megaruptor (Diagenode, Seraing, Belgium) followed by damage repair, end-repair, hairpin ligation, and size selection using BluePippin (Sage Science, Beverly, MA, USA). After primer annealing and polymerase binding, the DNA templates were sequenced on a PacBio RSII sequencer using P6/C4 chemistry and 360 min video time at the DNA Sequencing and Genomics Laboratory, Institute of Biotechnology, University of Helsinki, Finland[32].

Genome Assembly

The genome was assembled using the FALCON assembler (FALCON-Integrate-1.8.6)[26,27] with a read length cut-off of 18,000 bp. This cut-off was found to give the best contiguity for the assembly based on N50 value, while minimizing the percentage of possibly erroneous contigs. The erroneous contigs were detected by mapping markers of the linkage map from the previously published genome[25] to contigs, and calculating the percentage of chimeric contigs. We tested three different read length cut-offs 16,000 bp, 18,000 bp, and 20, 000 bp, all of which included approximately 9% of chimeric contigs. The assembly was based on 1,9M PacBio reads, 24,4 Gbp in total, with an N50 of 18,479 bp which is approximately 50x coverage based on the final genome size. With the selected read cut-off the data produced 10.8 Gb of corrected reads which were further assembled using the FALCON software. The assembly yielded 4,559 primary contigs containing 739.9 Mb with an N50 of 340 kb and 1,661 alternative contigs containing 118.1 Mb with an N50 of 85,246

bp. The alternative contigs were automatically separated by the FALCON pipeline. The data were also assembled using miniasm software (0.2-r137-dirty)[33] which yielded similar results. The larger than expected initial assembly size, approximately 1.5 times the k-mer estimate, is due to the multiple haplotypes originating from the 7 individuals used in sequencing.

To evaluate the putative chimeric contigs and assembly errors suggested by the genetic map, the raw SMRT sequencing data were mapped to the assembly primary contigs using the Burrows-Wheeler Aligner (BWA-0.7.17) with the MEM algorithm[34]. The alignments of the 425 regions discovered as possibly chimeric were visually inspected. Of these regions, 92 showed even read coverage and no evident signs of assembly errors, while 333 regions contained areas with low coverage and/or repeat regions indicated by high coverage that had led to erroneous overlaps and mis-assemblies. These errors were identified by positions where the majority of the reads did not fully align, i.e. the alignments ended mid-read. The assembly was split in the positions where the coverage was at minimum . The resulting assembly was polished using the SMRT sequencing data and Quiver[26] software from the SMRT Tools-package (PacBio).

Linkage Map

Linkage mapping was constructed from whole genome resequencing data of F2 crosses of *M. cinxia*. The grandparents of these F2 crosses are offspring of wild collected *M. cinxia* originating from two distantly related *M. cinxia* populations around the Baltic Sea; the Åland Islands (ÅL)[1] and Pieni Tytärsaari (PT) populations[35]. Between population crosses of type ÅL♂xPT♀ and ÅL♀xPT♂ were established to create the F1 population. Some of these F1 individuals were used to establish the F2 families, actively avoiding mating among siblings. A subset of the resulting full-sib families were reared to adulthood, and five of these

F2 families, together with their parents and grandparents, were selected for resequencing. In total, resequencing included ten grandparental individuals, ten F1 parents and 165 F2 individuals (N=185).

All the larvae from different generations completed development under common garden conditions (28:15°C; 12L:12D) utilizing fresh leaves of greenhouse grown *Veronica spicata*. Diapausing larvae were kept in a growth chamber at +5°C and 80% relative humidity for approximately seven months to mimic the normal wintertime conditions for these butterflies. Adults were kept in hanging cages (of 50 cm height and 40 cm diameter) at ~26:18°C; 9L:15, and fed *ad libitum* with 20% honey-water solution throughout the experiments.

Before DNA extraction the adult butterflies were stored at -80°C, and either thorax or abdomen tissue of these individuals was used for sequencing. Tissues were homogenized prior to extraction using TissueLyser (Qiagen, Venlo, The Netherlands) at 30/s for 1.5 mins with Tungsten Carbide Beads, 3 mm (Qiagen, Venlo, The Netherlands) and ATL buffer (Qiagen, Venlo, The Netherlands). DNA was extracted using the NucleoSpin 96 Tissue Core Kit (Macherey-Nagel) according to the manufacturer's protocol with the exception that lysing time was extended to overnight. The samples were additionally treated with RNase A (Thermo Scientific) before sequencing. Sequencing was performed using standard PE library preparation and Illumina Hi Seq 2000 with 125 bp paired-end reads.

The mapping procedure followed the Lep-MAP3[26] pipeline. First, individual fastq files were mapped to the contig assembly using BWA MEM (BWA-0.7.17) [34] and individual bam files were created using SAMtools (1.6)[36,37]. SAMtools mpileup and the scripts pileupParser2.awk and pileup2posterior.awk were used to obtain input data for Lep-MAP3. Then ParentCall2 (parameter: ZLimit=2) and Filtering2 (parameters: dataTolerance=0.0001; removeNonInformative=1; familyInformativeLimit=4) were run to obtain data with at least

four informative families for each marker, resulting in a final input with almost 2.5M markers.

SeparateChromosomes2 was run on the final data (parameters lodLimit=20; samplePair=0.2;numThreads=48) to obtain 31 linkage groups with a total of 2.4M markers. OrderMarkers2 was run (parameter recombination2=0) on each linkage group (chromosome). This map was used to anchor the contig assembly into chromosomes. To validate anchoring, the map construction was repeated in the same way except that OrderMarkers2 was run on the physical order of markers to reduce noise in the linkage map. Finally, the raw data were re-mapped to the gap-filled chromosome level assembly and the linkage map was re-done in the new physical order to infer final recombination rates.

Anchoring the genome and resolving haplotypes using the linkage map

The contigs were aligned against each other and lift-over chains were created by running the first two steps (batch A and B to calculate the alignment chain) of HaploMerger2[38] pipeline. By inspecting this chain, contigs fully contained in some longer contig were removed. Initial contig order and orientation within each chromosome was calculated by the median map position of each contig and the longest increasing subsequence of markers, respectively. For each chromosome, Marey map [39], a scatter plot of physical and linkage positions combining the genetic and physical maps, and contig-contig alignments from the chain were recorded. The contigs orders and orientations were manually fixed when needed if the map had support for alternative orientation or the contig-contig alignments were linking contigs together and any assembly errors that were found were corrected by splitting the contigs accordingly. Also, partially haplotypic contigs were found and collapsed based on the Marey maps and contig-contig alignments. This manual work resulted in the final reference genome sequence including start and end positions of contigs in the correct order and

orientation for each chromosome. Finally, the haplotype corrected genome was gap-filled using PBJelly software (PBSuite_15.8.24)[40] with the original SMRT sequencing data, and polished with the Quiver tool[26] from the SMRT Tools-package 2.3.0 (PacBio) and with Pilon (1.21)[41].

The chromosomes were aligned against the *Heliconius melpomene* (2.5)[42,43] and *Pieris napi*[44] genomes using the LAST aligner(938)[45] to check structural similarity between the species (Supplementary Figures S1-13). An overview alignment for *H. melpomene* was created using D-GENIES (1.2.0)[46] (Figure 2). The data show a high level of collinearity between *M. cinxia* and *H. melpomene* chromosomes, as described before in Ahola et al.[25]. A notably interesting point is the lack of collinearity with sex chromosomes (*M. cinxia* chromosome 1 & *H. melpomene* chromosome 21). Furthermore, the visible vertical lines show the effect of long read assembly on repeat resolution. With long reads spanning the repeats and allowing their accurate placement in the contigs, in *M. cinxia* the repeats are placed in single chromosomes whereas in *H. melpomene* they are present in all chromosomes.

Repeat masking and annotation

Genomic assemblies were masked with *de novo* repeat libraries by RepeatMasker v.4.0.9 (http://www.repeatmasker.org/). *De novo* repeat libraries were constructed from original PacBio reads with lengths over 30,000bp and assembled scaffolds (pseudo chromosomes) using RepeatModeler v 1.0.10 (http://www.repeatmasker.org/RepeatModeler/) and the LtrHarvest/LtrDigest-pipeline[47,48]. Repeat families were clustered using cd-hit-est applying 80/80-rule (80% identity over 80% length)[49]. Repeat annotations were confirmed by RepBase Release 20181026[50] and Dfam version 3.1[51].

Transcriptome assembly

In order to aid construction of gene models, we capitalised on two transcriptome assemblies which we produced as part of separate projects in our lab to be presented in upcoming publications ([5] Oostra et al. unpubl. data). Importantly for gene model construction, they represent a wide range of transcriptional diversity, as the RNAseq data are derived from various developmental stages (first instar larvae, fourth instar larvae, and adult thorax and abdomen). All individuals were lab-reared but originated from the same butterfly metapopulation. Transcriptome 1 was produced using a set of 78 individually sequenced female larvae (fourth developmental instar)[5], sequenced to an average depth of 17.3M reads (read lengths 85 bp and 65 bp for forward and reverse PE reads, respectively). As the two sexes are practically indistinguishable in the larval stages, the females were identified based on homozygosity across a set of 22 Z-chromosome specific SNP loci[5]. To remove Illumina adapter sequences, we trimmed raw reads using Trimmomatic (Trimmomatic-0.35)[52], and normalised using Trinity v2.6.5[53]. We then used two separate procedures to construct *de novo* transcriptome assemblies, Trinity (v2.6.5) and Velvet / Oases (1.2.10)[54]. Trinity was run with standard settings, whereas Velvet / Oases used a range of seven kmer sizes (21 bp to 71 bp), producing a separate assembly for each kmer size. We then combined the resulting assemblies, filtered the combined assembly using the EvidentialGene (tr2aacds.pl VERSION 2017.12.21)[55] pipeline, and removed contigs smaller than 200 bp or expressed at a low level (< 1 normalized counts per million), yielding the final assembly. Transcriptome 2 was constructed from a set of 12 adult females (thorax and abdomen, without ovaries) and 48 first instar larvae, as part of a separate gene expression study (Oostra et al. unpubl. data). RNA from these 60 individual samples was sequenced to an average depth of 16.6M reads (86/74 bp PE). The stranded RNA-seq libraries were made using Ovation® Universal RNA-Seq System (Nugen) with custom ribosomal RNA removal. The libraries were paired-end sequenced on a NextSeq 500 using the 150 bp kit (Illumina) at the DNA sequencing and

genomics laboratory Institute of Biotechnology University of Helsinki. We trimmed the reads using fastp (v0.20.0)[56], and used the HISAT2 (2.0.4) / StringTie (1.3.5) pipeline[57] to construct a genome-guided transcriptome assembly, mapping the RNAseq reads to the new genome assembly. Transcriptome 1 yielded 69,182 putative transcripts with average length of 727 bp (95% CI: 206 - 3433), while transcriptome 2 yielded 137,250 putative transcripts with average length of 1737 (95% CI: 203 - 9106). These statistics should be interpreted with caution, as the assemblies derive from different life stages, and different assembly and filtering approaches were used (reflecting differences in histories of the datasets as they were produced for different projects).

Gene model Annotation

Initial gene predictions were obtained by running the MAKER v 2.31.10[29] gene prediction programme in an iterative procedure. In the first round of MAKER, transcriptome assembly 1, described above, was provided as evidence, and genes were predicted solely from the aligned transcripts. This resulted in 14,738 gene models. These gene models were then used for training the SNAP (2013-02-16)[58] and AUGUSTUS (3.3.2)[59] gene predictors. A second round of MAKER was run providing the *de novo* transcripts from both transcriptomes (see previous paragraph), trained gene prediction models, repeat masking file and protein data from other lepidopteran species. The MAKER settings were adjusted to allow prediction of gene models without requiring a corresponding transcript in the *de novo* transcriptome assembly. Following each round of MAKER gene prediction, the annotation completeness was assessed using BUSCO[60,61].

Manual Annotation

Manual annotation was performed for 1,232 genes, using the Apollo collaborative annotation system version 2.1.0[62]. The collaborative annotation environment was set up in Ubuntu

Linux 14.04 server with 250 GB RAM and 48 AMD Opteron 6,168 processing cores. This was later upgraded to a cloud server provided by the Finnish IT Center for Science (CSC) and running on Ubuntu Linux 18.04 with 200 GB RAM and 40 Intel Xeon model 85 processing cores. Evidence tracks were produced containing gene predictions from three rounds of MAKER, RNASeq alignments of sequence reads and protein alignments from other species (Table 2). RNASeq alignments comprised a mixed tissue pooled sample, an abdomen pooled sample and six larval samples (from transcriptome 1) selected to represent a diverse range and included, for example, both sexes and different family backgrounds. A list of gene families that were considered of particular interest in butterfly research were identified for prioritisation during the manual annotation. (Supplementary File 4, Prioritized_gene_families.docx)). The gene annotators were able to select a family of genes for annotation or a random selection from the prioritized families was given. Gene models were corrected by examining the evidence tracks in the browser, conducting blast searches and examining multiple alignments of protein sequences. In total for the 1,232 genes, 1,455 mRNAs were manually inspected of which 814 genes and mRNAs were changed. Most changes were made to exon borders and mRNA exon structure, especially in the case of multiple isoforms.

Table 2. Evidence tracks that were used during the manual annotation of 1,232 *M. cinxia* genes

**Evidence track**	**Type**	**Description**
Maker 1	Gene prediction	Initial maker gene predictions based on EST alignments
Maker 2	Gene prediction	Second round of gene predictions from EST alignments, protein alignments and gene predictors

		trained on maker 1.
RNASeq abdomen pool	RNASeq alignment	RNASeq reads aligned to the genome with STAR[68]
RNASeq mixed tissue pool	RNASeq alignment	
B. mori proteins	Protein alignment	Proteins sequences aligned to the genome with AAT.
H. melpomene proteins	Protein alignment	
D. melanogaster proteins	Protein alignment	
H. erato proteins	Protein alignment	
RNASeq - female larvae family 80	RNASeq alignment	RNASeq reads aligned to the genome with STAR[68]
RNASeq - female larvae family 70	RNASeq alignment	
RNASeq female larvae family 119	RNASeq alignment	
RNASeq female larvae family 120	RNASeq alignment	
RNASeq male larvae family 80	RNASeq alignment	
RNASeq male larvae family 119	RNASeq alignment	

Final Gene Models

Following the manual annotation, the SNAP[58] and AUGUSTUS[59] gene predictors were retrained using the manually annotated gene models. MAKER was run using the updated gene predictors, transcriptome 1 and 2, and also using a masking file for repeats. As a final step to incorporate the manually annotated gene models, MAKER was run providing the

previous MAKER file to pred_gff and the manually annotated models to model_gff. Gene functional prediction was performed using Pannzer v2[63].

Ortholog identification

Predicted protein sequences from *Bombyx* mori[64] (January 2017 gene models), *P. napi*[44] and *H. melpomene* (Hmel2.5)[42,43] were downloaded from silkbase http://silkbase.ab.a.u-tokyo.ac.jp/cgi-bin/download.cgi, LepBase[65] and the Butterfly Genome Database http://butterflygenome.org respectively. OrthoFinder v2.3.3[66] was run to identify orthologs between *M. cinxia*, *B. mori, P napi* and *H. melpomene* using blast as the search tool (Figure 3 & Supplementary Figure S14).

Data Validation and quality control

To assess the quality of the assembly, assembly statistics were generated using assembly-stats[65] and compared to the v1 genome as well as the *H. melpomene*, *B. mori* and *P. napi* genome assemblies (Table 1). The new genome contains 94 Mb more sequence than the previous scaffold assembly. Based on the observations of individual alignments in the full genome alignment between the version 1 and version 2, there are many regions in the genome 1 which are aligned into multiple positions in version 2. This points to collapsed repeat regions in version 1 and more accurate repeat placement due to the long read sequencing in version 2. The N50 length and L50 value at scaffold or chromosome level have improved greatly compared to the previous genome. To check for possible duplication or missing areas in the assembly, an assessment was made of the completeness of single copy orthologs from BUSCO[60,61] eukaryota, arthropoda and metazoa gene sets (Table 3). In each of the gene sets, 93.4-94.9% of the expected single copy orthologs were found in complete and single copies. The duplication rate was estimated to be between 1.4 and 2.3%. A total of 1,232 gene models were manually curated using the Apollo annotation system[62] to ensure the quality

of the models. To test for contamination, the predicted protein sequences were checked with AAI-profiler[67] to identify sequences originating from different taxa (Supplementary Files 1-3 (AAI.html, matrix.html, krona.html)). Overall, 42% of the genome was composed of repeat sequences (Figure 4 and Supplementary Figures S15-20 (chromosome specific repeat classes)). There were no clear differences in the repeat contents between chromosomes (Supplementary Table 1) which further supports the more accurate placement of repeats due to the long read sequencing in version 2. Long interspersed elements (LINE) were the most prevalent.

Table 3. BUSCO completeness estimates of the v2 genome based on the eukaryota, arthropoda and metazoa gene sets.

**Lineage**	**BUSCO Category**				
	**Complete**	**Single-copy**	**Duplicated**	**Fragmented**	**Missing**
Eukaryota	290	283	7	5	8
	95.7%	93.4%	2.3%	1.7%	2.6%
Arthropoda	1027	1012	15	7	32
	96.3%	94.9%	1.4%	0.7%	3.0%
Metazoa	935	921	14	12	31
	95.6%	94.2%	1.4%	1.2%	3.2%

Re-use potential

The substantial improvements in contiguity and gene annotation quality of the new genome will enable a range of important new studies and open up possibilities for future work. The results also demonstrate that with the use of proper computational tools and data, it is

possible to obtain a high quality, chromosome scale reference genome even when a single individual organism will not provide enough high molecular weight DNA for long read sequencing. Furthermore, we show the potential of the linkage mapping: it anchors contigs to actual chromosomes, instead of just linking different contigs together as is done for example in Hi-C approach. Moreover, the haplotype problem is not tackled by Hi-C. Our high-density linkage map allows us to put basically all contigs into chromosomes. It is worth noting that the linkage map is not scaffolding directly but it will put contigs into map positions. Scaffolding is possible if a contig spans two or more map positions. Otherwise the contig can be placed only partially. In addition to the linkage map approach, we used extensive manual curation of the assembly to avoid chimeric parts and improve the assembly quality. Current research aims at identifying mechanisms underlying key life history adaptations, exploring the extent of natural variation and selection on these adaptations in wild populations, and integrating these insights with the exceptional ecological, demographic, and climatic data available for this system. Future studies in this direction will help understand the mechanisms maintaining variation in life-histories across spatial and temporal scales, and on the extent to which phenotypic variation in these and other traits may contribute to a population's adaptive capacity under climate change. Several studies in different species illustrate how stress responses can be crucial for survival under variable environments, both within and between generations. The Glanville fritillary is being used to explore how environmental information is translated into adaptive phenotypic changes, and how these responses are transmitted to future generations, using transcriptomic and epigenetic approaches. Such studies will greatly benefit from an improved annotation permitting exon-specific expression quantification, and identification of epigenetic marks and other functional variants outside coding regions. Exploiting current and past large-scale sampling efforts, these new studies apply population genomic approaches that are greatly facilitated by the increased assembly contiguity, for

instance by permitting linkage disequilibrium (LD) and haplotype-based selection analyses. Other avenues of research enabled by the improved genome assembly include structural variation, regulatory evolution, recombination rate variation, and coalescent-based demographic analyses. The increasing availability of chromosome-level lepidopteran genomes such as ours permits exciting new comparative phylogenetic analyses, for example of chromosome and genome evolution.

Availability of source code and requirements (if used in the paper)

Not applicable

Availability of supporting data

The SMRT sequencing reads used for the genome assembly have been deposited to the sequence read archive under Bioproject PRJNA607899 accession number SRR11184190.

The genome has been deposited to GenBank under Bioproject PRJNA607899

The Illumina reads used for the linkage map have been deposited to the sequence read archive under Bioproject PRJNA608928 accession numbers SRR11186917- SRR11187107.

Transcriptome 1 RNASeq reads have been deposited to NCBI GEO under accession number GSE159376

Transcriptome 2 RNASeq reads have been deposited to NCBI SRA under ioproject PRJNA670126

Declarations

List of abbreviations

Not applicable

Ethics approval and consent to participate

There are no ethical policies related to working with insect data. The Glanville fritillary is not considered endangered in the Åland islands and no permits are required for sampling. However, we note that within this project the larval sampling for genetic analyses is done non-invasively in the field. ensuring insignificant demographic impact. In addition, as the sampling will take place prior diapause (Åland) when mortality is generally the highest – the collection has negligible effect on the family survival or the demography of populations.

Consent for publication

Not Applicable

Competing interests

'The authors declare that they have no competing interests'.

Funding

Funding for M.S, D.B, V.O, E.vB, J.T & A.K was provided by a grant from the European Research Council (Independent Starting Grant No. 637412 'META-STRESS' to MS) and J Ö-U, V.A and D.B from the Academy of Finland grant (Decision No. 304041 to MS & Decision No. 283108 to Ilkka Hanski). A.D was funded by a Marie Sklodowska Curie Individual Fellowship (#790531, Host Sweet Home). O-P.S. was supported by the "TTÜ development program 2016– 2022", project code 2014-2020.4.01.16-0032.

Authors' contributions

O-P.S assembled the genome, processed the chimeric contigs, performed the gap filling and the polishing of the assembly, and participated in the genome analysis.

V.A was responsible for the initial idea of the approach for the genome related activities, coordinated the first part of the project, designed and produced data for the linkage map, and worked on solving the haplotypes from the initial assembly.

D.B performed gene prediction, functional annotation, ortholog prediction, and managed the manual annotation.

J.K installed and managed the Apollo annotation server.

S.I was responsible for larval rearing and preparation of butterfly crosses.

P.R performed the linkage mapping and anchored the genome onto chromosomes.

V.O assembled the transcriptomes used for gene prediction.

Lo.P manually inspected the chimeric contigs.

A.R performed DNA extraction.

D.B, J.K, V.O, T.D, M.F.D, A.D, I.C.D, P.H, A.K, S.S.K, S.O.K, E.L, S.L, J.M, A.N, M.C-M, V.P, T.S, A.I.T, V.T, E.vB, J.Ö-U and M.S participated in manual annotation.

J.T performed the annotation of transposable elements and repeat classes.

L.P was responsible for the management of the DNA sequencing.

M.J.F was responsible for the management of the genome analysis.

P.A was responsible for the initial idea of the approach for the genome related activities, and the management of the genome analysis.

M.S was responsible for the management of the *M. cinxia* database and genome analysis.

O-P.S, D.B, V.A, P.R, J.T, J.K, V.O, L.P, M.J.F, P.A and M.S wrote the manuscript.

Acknowledgements

The authors wish to acknowledge CSC – IT Center for Science, Finland, for computational resources. We thank Torsti Schulz and Emily Hornett for annotating > 10 genes. We thank the personnel of the DNA sequencing and genomics laboratory (Institute of Biotechnology, Helsinki, Finland) for performing the NGS sequencing.

Authors' information

[1] Institute of Biotechnology, University of Helsinki, Finland

[2] Organismal and Evolutionary Biology Research Programme, University of Helsinki, Finland

[3] Department of Chemistry and Biotechnology, Tallinn University of Technology, Estonia

[4] Ming Wai Lau Centre for Reparative Medicine, Karolinska Institutet, Hong Kong

[5] Natural Resource Institute (LUKE), Finland

[6] Department of Evolution, Ecology and Behaviour, University of Liverpool, UK

[7] Department of Biological Sciences, Louisiana State University, USA

[8] Department of Biology, Lund University, Sweden

[9] Department of Environmental and Biological Sciences, University of Eastern Finland, Finland

[10] Department of Entomology and Nematology, University of Florida, USA

[11.] Department of Zoology, Loknete Vyankatrao Hiray Arts, Science & Commerce College, India

[12.] Swiss Federal Institute of Aquatic Science and Technology, Department of Aquatic Ecology, Switzerland

[13.] College of Plant Protection, China Agricultural University, China

[14.] Department of Zoology, Stockholm University, Sweden

[15.] Viikki Plant Science Centre, Organismal and Evolutionary Biology Research Programme, University of Helsinki, Finland

[16.] Helsinki Institute of Life Science (HiLIFE), University of Helsinki, Finland

Present address:

Lo.P - School of Life Sciences, University of Warwick, UK

V.T - Luomus, Finnish Museum of Natural History, Zoology Unit, University of Helsinki, Helsinki, Finland

S.O.K, P.H and J.M - Biosafe – Biological Safety Solutions Ltd, Microkatu 1 M, 70210 Kuopio, Finland

corresponding author(s): marjo.saastamoinen@helsinki.fi, olli-pekka.smolander@taltech.ee

Endnotes

Not applicable

Figure 1. An overview of the assembly and annotation process of the improved Glanville fritillary genome.

Figure 2. A dot-plot structural comparison of the *H. melpomene* genome against the *M. cinxia* v2 genome.

The alignment was created using D-GENIES (1.2.0)[46]. The diagonal lines indicate the collinearity between the two species. The lack of collinearity in sex chromosomes is visible in the upper left corner between Mcnxia_v2 chr 01 and Hmel2.5 chr 21. The visible vertical lines show repeats that are resolved in Mcinxia_v2 but are present in all chromosomes in Hmel2.5_chr.

Figure 3. A circos plot showing the orthologs between *M. cinxia* and *H. melpomene*

Orthologs between *M. cinxia* and *H. melpomene* were identifies using OrthoFinder and filtered for one-to-one orthologs. The internal links in the circos plot indicate the orthologs between *M. cinxia* and *H. melpomene*. The links are coloured according to the *M. cinxia* chromosome.

Figure 4. Relative amounts of different repeat classes in *M. cinxia* genome

Repeat classes and coverage of the *M. cinxia* genome v2: DNA = classII; LINE = Long interspersed elements; LTR = Long terminal repeats; LOW_COMPLEXITY = Low complexity repeated DNA; RC = Rolling circle elements (e.g. Helitrons); SINE = Short interspersed elements; Satellite = Satellite DNA; SIMPLE_REPEAT = Simple repeated motifs; EXON = Exonic regions; UNCOVERED = rest of the chromosomes.

References

1 Hanski, I. Habitat connectivity, habitat continuity, and metapopulations in dynamic landscapes. *Oikos* **87**, 209-219, doi:Doi 10.2307/3546736 (1999).

2 Ovaskainen, O. & Saastamoinen, M. Frontiers in Metapopulation Biology: The Legacy of Ilkka Hanski. *Annu Rev Ecol Evol S* **49**, 231-252, doi:10.1146/annurev-ecolsys-110617-062519 (2018).

3 Ojanen, S. P., Nieminen, M., Meyke, E., Pöyry, J. & Hanski, I. Long-term metapopulation study of the Glanville fritillary butterfly (*Melitaea cinxia*): survey methods, data management, and long-term population trends. *Ecol Evol* **3**, 3713-3737, doi:10.1002/ece3.733 (2013).

4 Saastamoinen, M., Hirai, N. & van Nouhuys, S. Direct and trans-generational responses to food deprivation during development in the Glanville fritillary butterfly. *Oecologia* **171**, 93-104, doi:10.1007/s00442-012-2412-y (2013).

5 Kahilainen, A., Oostra, V., Somervuo, P., Minard, G., & Saastamoinen, M. Alternative developmental and transcriptomic responses to host plant water limitation in a butterfly metapopulation. bioRxiv, 2021.02.24.432453 (2021).

6 Hanski, I., Saastamoinen, M. & Ovaskainen, O. Dispersal-related life-history trade-offs in a butterfly metapopulation. *J Anim Ecol* **75**, 91-100, doi:10.1111/j.1365-2656.2005.01024.x (2006).

7 Niitepõld, K. *et al.* Flight metabolic rate and Pgi genotype influence butterfly dispersal rate in the field. *Ecology* **90**, 2223-2232, doi:Doi 10.1890/08-1498.1 (2009).

8 Hanski, I. & Singer, M. C. Extinction-colonization dynamics and host-plant choice in butterfly metapopulations. *Am Nat* **158**, 341-353, doi:Doi 10.1086/321985 (2001).

9 Rosa, E., Woestmann, L., Biere, A. & Saastamoinen, M. A plant pathogen modulates the effects of secondary metabolites on the performance and immune function of an insect herbivore. *Oikos* **127**, 1539-1549, doi:10.1111/oik.05437 (2018).

10 Rosa, E., Minard, G., Lindholm, J. & Saastamoinen, M. Moderate plant water stress improves larval development, and impacts immunity and gut microbiota of a specialist herbivore. *Plos One* **14**, doi:ARTN e020429210.1371/journal.pone.0204292 (2019).

11 Salgado, A. L. & Saastamoinen, M. Developmental stage-dependent response and preference for host plant quality in an insect herbivore. *Anim Behav* **150**, 27-38, doi:10.1016/j.anbehav.2019.01.018 (2019).

12 Van Nouhuys, S. & Lei, G. C. Parasitoid-host metapopulation dynamics: the causes and consequences of phenological asynchrony. *J Anim Ecol* **73**, 526-535, doi:DOI 10.1111/j.0021-8790.2004.00827.x (2004).

13 de Jong, M. A. & Saastamoinen, M. Environmental and genetic control of cold tolerance in the Glanville fritillary butterfly. *J Evolution Biol* **31**, 636-645, doi:10.1111/jeb.13247 (2018).

14 Saastamoinen, M., Ikonen, S., Wong, S. C., Lehtonen, R. & Hanski, I. Plastic larval development in a butterfly has complex environmental and genetic causes and consequences for population dynamics. *J Anim Ecol* **82**, 529-539, doi:10.1111/1365-2656.12034 (2013).

15 Niitepõld, K. & Saastamoinen, M. A candidate gene in an ecological model species: Phosphoglucose isomerase (Pgi) in the Glanville fritillary butterfly (*Melitaea cinxia*). *Ann Zool Fenn* **54**, 259-273, doi:Doi 10.5735/086.054.0122 (2017).

16 de Jong, M. A., Wong, S. C., Lehtonen, R. & Hanski, I. Cytochrome P450 gene CYP337 and heritability of fitness traits in the Glanville fritillary butterfly. *Mol Ecol* **23**, 1994-2005, doi:10.1111/mec.12697 (2014).

17 Fountain, T. *et al.* Predictable allele frequency changes due to habitat fragmentation in the Glanville fritillary butterfly (vol 113, pg 2678, 2016). *P Natl Acad Sci USA* **113**, E5363-E5363, doi:10.1073/pnas.1613041113 (2016).

18 Fountain, T. *et al.* Inferring dispersal across a fragmented landscape using reconstructed families in the Glanville fritillary butterfly. *Evol Appl* **11**, 287-297, doi:10.1111/eva.12552 (2018).

19 Dileo, M. F., Husby, A. & Saastamoinen, M. Landscape permeability and individual variation in a dispersal-linked gene jointly determine genetic structure in the Glanville fritillary butterfly. *Evol Lett* **2**, 544-556, doi:10.1002/evl3.90 (2018).

20 Haag, C. R., Saastamoinen, M., Marden, J. H. & Hanski, I. A candidate locus for variation in dispersal rate in a butterfly metapopulation. *P Roy Soc B-Biol Sci* **272**, 2449-2456, doi:10.1098/rspb.2005.3235 (2005).

21 Mattila, A. L. K. & Hanski, I. Heritability of flight and resting metabolic rates in the Glanville fritillary butterfly. *J Evolution Biol* **27**, 1733-1743, doi:10.1111/jeb.12426 (2014).

22 Klemme, I. & Hanski, I. Heritability of and strong single gene (Pgi) effects on life-history traits in the Glanville fritillary butterfly. *J Evolution Biol* **22**, 1944-1953, doi:10.1111/j.1420-9101.2009.01807.x (2009).

23 Kvist, J. *et al.* Flight-induced changes in gene expression in the Glanville fritillary butterfly. *Mol Ecol* **24**, 4886-4900, doi:10.1111/mec.13359 (2015).

24 Kvist, J. *et al.* Temperature treatments during larval development reveal extensive heritable and plastic variation in gene expression and life history traits. *Mol Ecol* **22**, 602-619, doi:10.1111/j.1365-294X.2012.05521.x (2013).

25 Ahola, V. *et al.* The Glanville fritillary genome retains an ancient karyotype and reveals selective chromosomal fusions in Lepidoptera. *Nat Commun* **5**, doi:ARTN 473710.1038/ncomms5737 (2014).

26 Rastas, P., Paulin, L., Hanski, I., Lehtonen, R. & Auvinen, P. Lep-MAP: fast and accurate linkage map construction for large SNP datasets. *Bioinformatics* **29**, 3128-3134, doi:10.1093/bioinformatics/btt563 (2013).

27 Chin, C. S. *et al.* Nonhybrid, finished microbial genome assemblies from long-read SMRT sequencing data. *Nat Methods* **10**, 563-+, doi:10.1038/Nmeth.2474 (2013).

28 Chin, C. S. *et al.* Phased diploid genome assembly with single-molecule real-time sequencing. *Nat Methods* **13**, 1050-+, doi:10.1038/Nmeth.4035 (2016).

29 Campbell, M. S., Holt, C., Moore, B. & Yandell, M. Genome Annotation and Curation Using MAKER and MAKER-P. *Curr Protoc Bioinformatics* **48**, 4.11.11-39 (2014).

30 Thomas, C. D. & Hanski, I. in *Ecology, genetics and evolution of metapopulations* 489-514 (Elsevier, 2004).

31 van Bergen, E. *et al.* Summer drought decreases the predictability of local extinctions in a butterfly metapopulation. Preprint at https://doi.org/10.1101/863795 (2019).

32 Salojärvi, J. *et al.* Genome sequencing and population genomic analyses provide insights into the adaptive landscape of silver birch. *Nat Genet* **49**, 904-912 (2017).

33 Li, H. Minimap and miniasm: fast mapping and de novo assembly for noisy long sequences. *Bioinformatics* **32**, 2103-2110, doi:10.1093/bioinformatics/btw152 (2016).

34 Li, H. Aligning sequence reads, clone sequences and assembly contigs with BWA-MEM. Preprint at *arXiv:1303.3997* (2013).

35 Mattila, A. L. K. *et al.* High genetic load in an old isolated butterfly population. *P Natl Acad Sci USA* **109**, E2496-E2505, doi:10.1073/pnas.1205789109 (2012).

36 Li, H. A statistical framework for SNP calling, mutation discovery, association mapping and population genetical parameter estimation from sequencing data. *Bioinformatics (Oxford, England)* **27**, 2987-2993 (2011).

37 Li, H. *et al.* The Sequence Alignment/Map format and SAMtools. *Bioinformatics (Oxford, England)* **25**, 2078-2079 (2009).

38 Huang, S., Kang, M. & Xu, A. HaploMerger2: rebuilding both haploid sub-assemblies from high-heterozygosity diploid genome assembly. *Bioinformatics (Oxford, England)* **33**, 2577-2579 (2017).

39 Chakravarti, A. A graphical representation of genetic and physical maps: the Marey map. Genomics. 11, 219-22. doi: 10.1016/0888-7543(91)90123-v (1991).

40 English, A. C. *et al.* Mind the Gap: Upgrading Genomes with Pacific Biosciences RS Long-Read Sequencing Technology. *Plos One* **7**, doi:ARTN e4776810.1371/journal.pone.0047768 (2012).

41 Walker, B. J. *et al.* Pilon: An Integrated Tool for Comprehensive Microbial Variant Detection and Genome Assembly Improvement. *Plos One* **9**, doi:ARTN e11296310.1371/journal.pone.0112963 (2014).

42 Davey, J. W. *et al.* No evidence for maintenance of a sympatric Heliconius species barrier by chromosomal inversions. *Evol Lett* **1**, 138-154, doi:10.1002/evl3.12 (2017).

43 Davey, J. W. *et al.* Major Improvements to the *Heliconius melpomene* Genome Assembly Used to Confirm 10 Chromosome Fusion Events in 6 Million Years of Butterfly Evolution. *G3-Genes Genom Genet* **6**, 695-708, doi:10.1534/g3.115.023655 (2016).

44 Hill, J. *et al.* Unprecedented reorganization of holocentric chromosomes provides insights into the enigma of lepidopteran chromosome evolution. *Sci Adv* **5**, doi:ARTN eaau3648 10.1126/sciadv.aau3648 (2019).

45 Kielbasa, S. M., Wan, R., Sato, K., Horton, P. & Frith, M. C. Adaptive seeds tame genomic sequence comparison. *Genome Res* **21**, 487-493, doi:10.1101/gr.113985.110 (2011).

46 Cabanettes, F. & Klopp, C. D-GENIES: dot plot large genomes in an interactive, efficient and simple way. *Peerj* **6**, doi:ARTN e495810.7717/peerj.4958 (2018).

47 Ellinghaus, D., Kurtz, S. & Willhoeft, U. LTRharvest, an efficient and flexible software for de novo detection of LTR retrotransposons. *BMC Bioinformatics* **9**, doi:Artn 1810.1186/1471-2105-9-18 (2008).

48 Steinbiss, S., Willhoeft, U., Gremme, G. & Kurtz, S. Fine-grained annotation and classification of de novo predicted LTR retrotransposons. *Nucleic Acids Res* **37**, 7002-7013, doi:10.1093/nar/gkp759 (2009).

49 Fu, L. M., Niu, B. F., Zhu, Z. W., Wu, S. T. & Li, W. Z. CD-HIT: accelerated for clustering the next-generation sequencing data. *Bioinformatics* **28**, 3150-3152, doi:10.1093/bioinformatics/bts565 (2012).

50 Jurka, J. Repbase Update - a database and an electronic journal of repetitive elements. *Trends Genet* **16**, 418-420, doi:Doi 10.1016/S0168-9525(00)02093-X (2000).

51 Hubley, R. *et al.* The Dfam database of repetitive DNA families. *Nucleic Acids Res* **44**, D81-D89, doi:10.1093/nar/gkv1272 (2016).

52 Bolger, A. M., Lohse, M. & Usadel, B. Trimmomatic: a flexible trimmer for Illumina sequence data. *Bioinformatics (Oxford, England)* **30**, 2114-2120 (2014).

53 Grabherr, M. G. *et al.* Full-length transcriptome assembly from RNA-Seq data without a reference genome. *Nat Biotechnol* **29**, 644-652 (2011).

54 Schulz, M. H., Zerbino, D. R., Vingron, M. & Birney, E. Oases: robust de novo RNA-seq assembly across the dynamic range of expression levels. *Bioinformatics (Oxford, England)* **28**, 1086-1092 (2012).

55 Gilbert, D. Gene-omes built from mRNA-seq not genome DNA. (2013).

56 Chen, S., Zhou, Y., Chen, Y. & Gu, J. fastp: an ultra-fast all-in-one FASTQ preprocessor. *Bioinformatics (Oxford, England)* **34**, i884-i890 (2018).

57 Pertea, M., Kim, D., Pertea, G. M., Leek, J. T. & Salzberg, S. L. Transcript-level expression analysis of RNA-seq experiments with HISAT, StringTie and Ballgown. *Nat Protoc* **11**, 1650-1667 (2016).

58 Korf, I. Gene finding in novel genomes. *BMC Bioinformatics* **5**, 59 (2004).

59 Lomsadze, A., Ter-Hovhannisyan, V., Chernoff, Y. O. & Borodovsky, M. Gene identification in novel eukaryotic genomes by self-training algorithm. *Nucleic Acids Res* **33**, 6494-6506 (2005).

60 Simao, F. A., Waterhouse, R. M., Ioannidis, P., Kriventseva, E. V. & Zdobnov, E. M. BUSCO: assessing genome assembly and annotation completeness with single-copy orthologs. *Bioinformatics* **31**, 3210-3212, doi:10.1093/bioinformatics/btv351 (2015).

61 Waterhouse, R. M. *et al.* BUSCO Applications from Quality Assessments to Gene Prediction and Phylogenomics. *Mol Biol Evol* **35**, 543-548, doi:10.1093/molbev/msx319 (2018).

62 Dunn, N. A. *et al.* Apollo: Democratizing genome annotation. *PLoS Comput Biol* **15**, doi:ARTN e100679010.1371/journal.pcbi.1006790 (2019).

63 Törönen, P., Medlar, A. & Holm, L. PANNZER2: a rapid functional annotation web server. *Nucleic Acids Res* **46**, W84-W88, doi:10.1093/nar/gky350 (2018).

64 Kawamoto, M. *et al.* High-quality genome assembly of the silkworm, *Bombyx mori*. *Insect Biochem Mol Biol* **107**, 53-62, doi:10.1016/j.ibmb.2019.02.002 (2019).

65 Challis, R. J., Kumar, S., Dasmahapatra, K. K., Jiggins, C. D. & Blaxter, M. Lepbase: the Lepidopteran genome database. Preprint at https://www.biorxiv.org/content/10.1101/056994v1.abstract doi:10.1101/056994 (2016).

66 Emms, D. M. & Kelly, S. OrthoFinder: phylogenetic orthology inference for comparative genomics. *Genome Biol* **20**, doi:ARTN 23810.1186/s13059-019-1832-y (2019).

67 Medlar, A. J., Törönen, P. & Holm, L. AAI-profiler: fast proteome-wide exploratory analysis reveals taxonomic identity, misclassification and contamination. *Nucleic Acids Res* **46**, W479-W485, doi:10.1093/nar/gky359 (2018).

68 Dobin, A. *et al.* STAR: ultrafast universal RNA-seq aligner. *Bioinformatics* **29**, 15-21, doi:10.1093/bioinformatics/bts635 (2013).

 Click here to access/download;Figure;Fig1.png

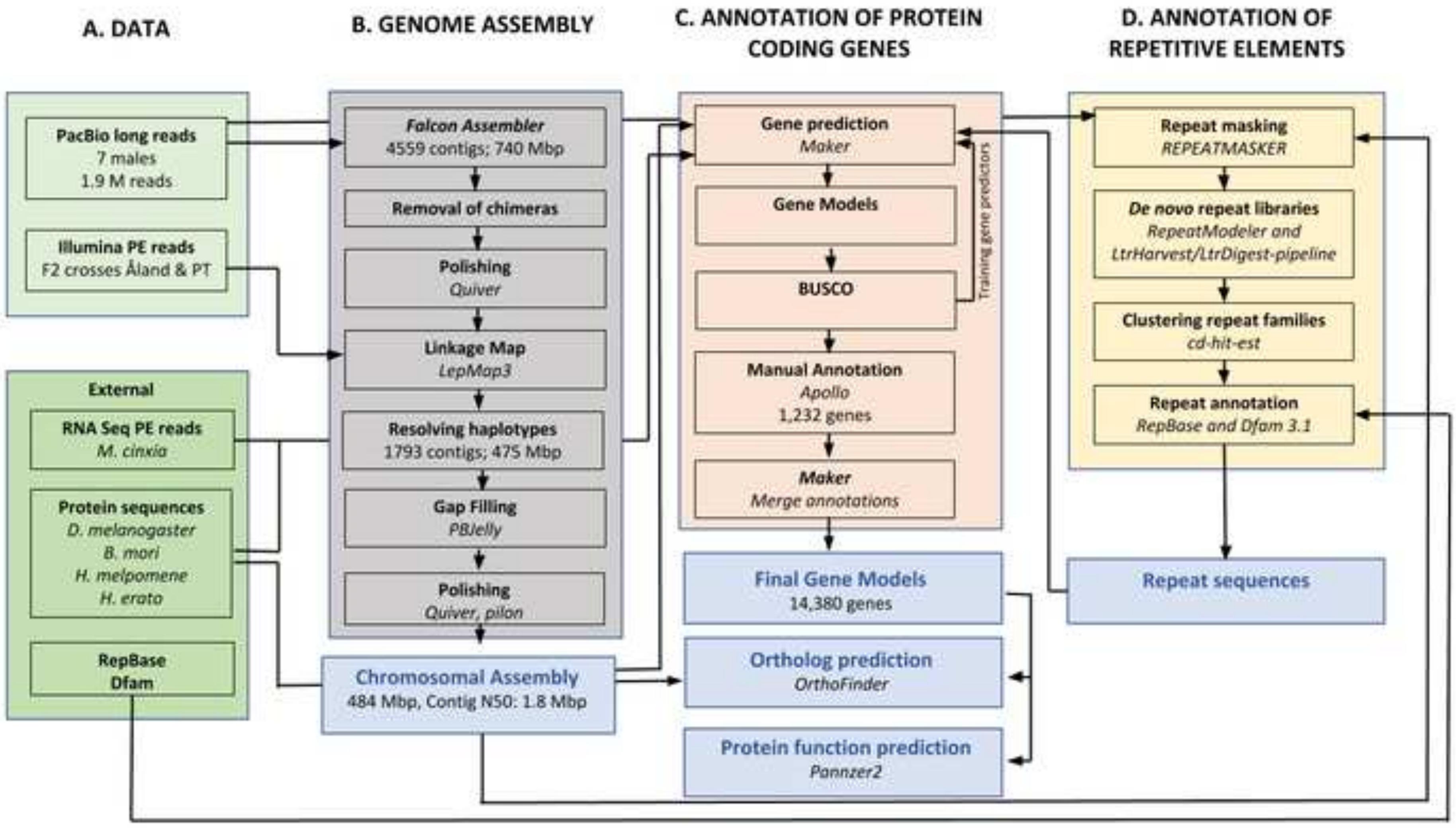

Figure

Click here to access/download;Figure;Fig2.png

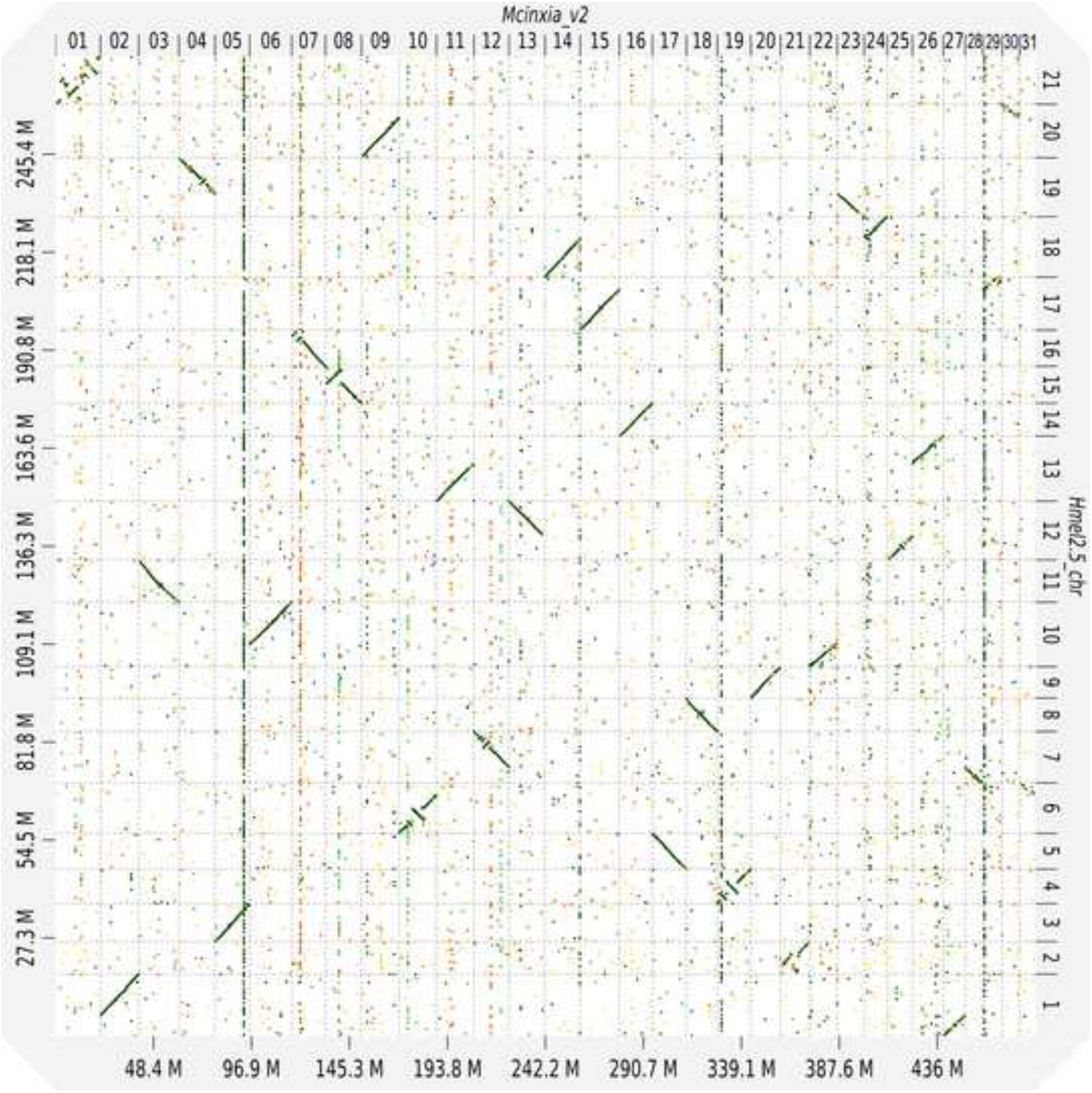

Figure

Click here to access/download;Figure;Fig3.png

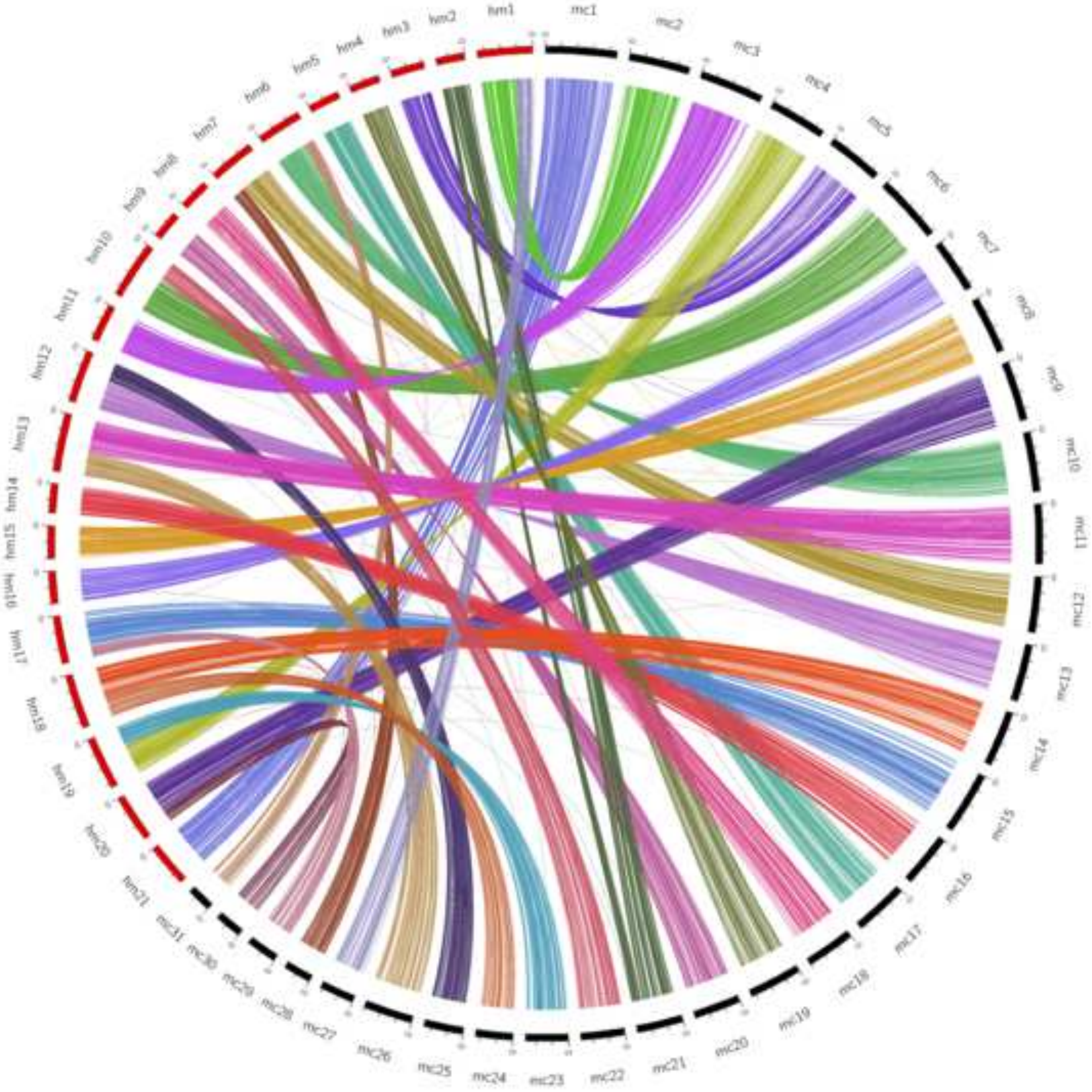

Click here to access/download;Figure;Fig4.png

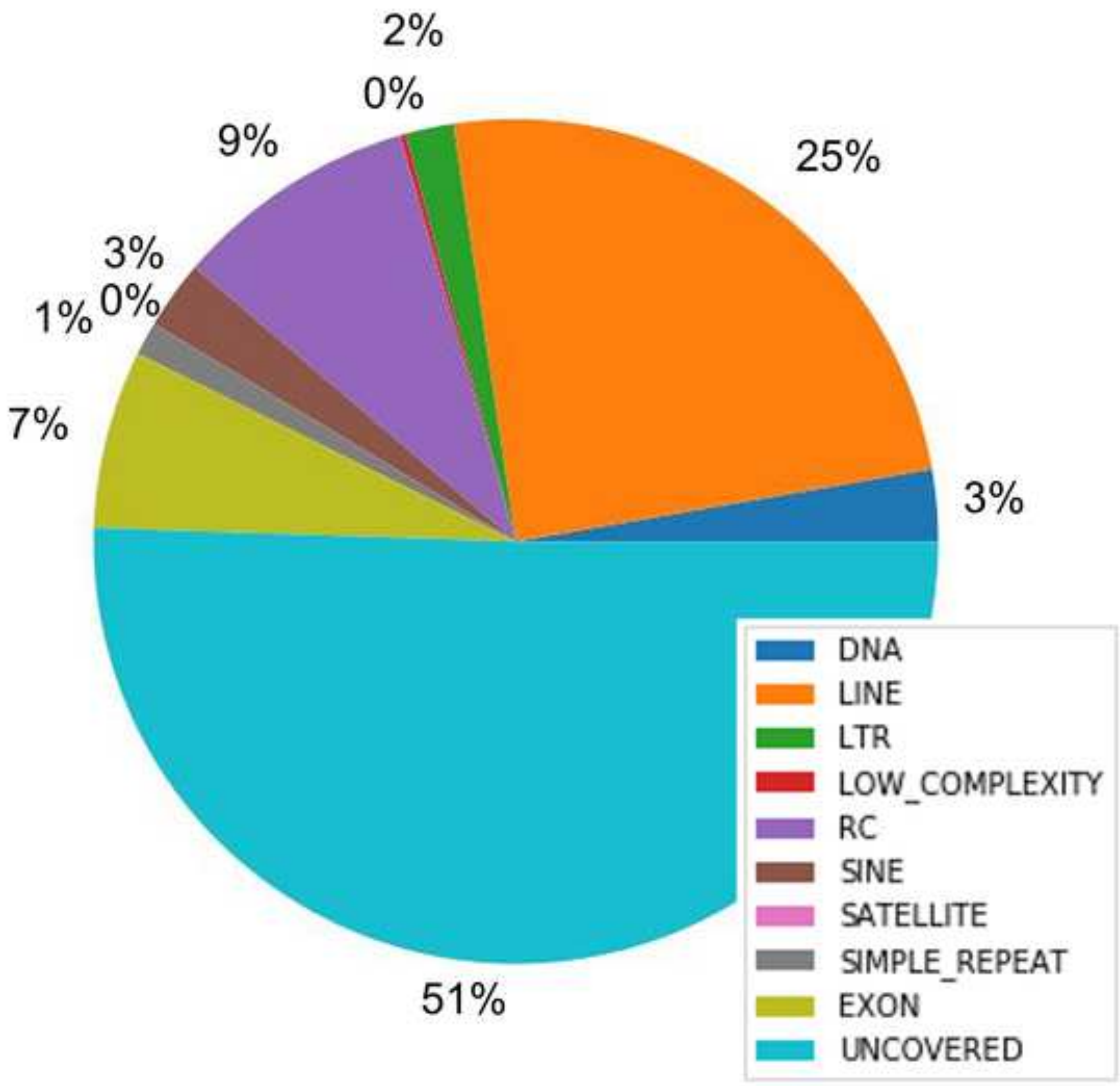

Click here to access/download
Supplementary Material
Supplementary_Figures.docx

Click here to access/download
Supplementary Material
AAI.html

Click here to access/download
Supplementary Material
krona.html

Click here to access/download
Supplementary Material
matrix.html

Click here to access/download
Supplementary Material
Supplementary_table1.xlsx

Click here to access/download
Supplementary Material
Prioritized_gene_families.docx

Click here to access/download
Supplementary Material
Kmer_analysis_for_genome_size.docx
