# Supplementary material for: Improved chromosome-level genome assembly of the Glanville fritillary butterfly (Melitaea cinxia) integrating Pacific Biosciences long reads and a high-density linkage map
Source: Gigascience. 2022 Jan 12;11:giab097. doi: 10.1093/gigascience/giab097 (PMC8756199; doi:10.1093/gigascience/giab097)
Supplement: giab097_Supplemental_Files [file giab097_supplemental_files.zip › matrix.html]

Browser compatibility: Chrome recommended. Windows Edge does not display Plotly heatmap correctly.
