# Supplementary material for: Improved chromosome-level genome assembly of the Glanville fritillary butterfly (Melitaea cinxia) integrating Pacific Biosciences long reads and a high-density linkage map
Source: Gigascience. 2022 Jan 12;11:giab097. doi: 10.1093/gigascience/giab097 (PMC8756199; doi:10.1093/gigascience/giab097)
Supplement: giab097_Supplemental_Files [file giab097_supplemental_files.zip › Prioritized_gene_families.docx]

**Gene families selected based on particular interest in butterfly research**

JAK/STAT and JNK pathways

Toll pathway

IMD pathway

Prophenoloxidases

Other proteases

Other immunity related genes

Odorant binding proteins

Circadian rhythm

Ionotropic Receptor

Storage proteins

HIF pathway

HOX genes

Glycolysis pathway

Growth GO:0040008

Muscle structure development

Heart development

Structural constituent of muscle

Intermediate filament

Locomotion, flight

Trehalases

Other muscle related

Heat shock proteins

Other chaperone related genes

Cytochrome P449

Cytochrome P450

Cytochrome P451

Cytochrome P452

Hormonal activity

Cuticular proteins and cuticle development

Esterases

Ribosomal proteins

Z chromosome
