# Supplementary material for: Improved chromosome-level genome assembly of the Glanville fritillary butterfly (Melitaea cinxia) integrating Pacific Biosciences long reads and a high-density linkage map
Source: Gigascience. 2022 Jan 12;11:giab097. doi: 10.1093/gigascience/giab097 (PMC8756199; doi:10.1093/gigascience/giab097)
Supplement: giab097_Response_to_Reviewer_Comments_Revision_2 [file giab097_response_to_reviewer_comments_revision_2.pdf]

# Author's Response To Reviewer Comments

Close

The minor editions required by the editor have been implemented and the figure 1 updated accordingly.

Close
