# Supplementary material for: Improved chromosome-level genome assembly of the Glanville fritillary butterfly (Melitaea cinxia) integrating Pacific Biosciences long reads and a high-density linkage map
Source: Gigascience. 2022 Jan 12;11:giab097. doi: 10.1093/gigascience/giab097 (PMC8756199; doi:10.1093/gigascience/giab097)
Supplement: giab097_Reviewer_1_Report_Original_Submission — Annabel Charlotte Whibley -- 1/12/2021 Reviewed [file giab097_reviewer_1_report_original_submission.pdf]

Reviewer Report

Title: Improved chromosome-level genome assembly of the Glanville fritillary butterfly (Melitaea cinxia) integrating PacBio long reads and a high-density linkage map.

Version: Original Submission **Date:** 1/12/2021

Reviewer name: Annabel Charlotte Whibley

Reviewer Comments to Author:

In this manuscript, Blande, Smolander and colleagues report an improved chromosome-level genome assembly of the important ecological model lepidopteran species Melitaea cinxta. The manuscript would benefit from further language review by a native English speaker to improve readability, but the intentions of the authors are nevertheless clearly articulated throughout, the workflow is logical, and the assembly quality is a clear improvement on the earlier draft release.
I would suggest revisiting the title to better reflect the work- as it stands it is a little underwhelming. One suggestion would be "Improved chromosome-level genome assembly of the Glanville fritillary butterfly (Melitaea cinxia) integrating PacBio long reads and a high-density linkage map". I would ideally also like to see more discussion of the more unusual aspects of this project- for example, long-read assemblies are commonplace now, but the linkage map approach (and the extent to which there was manual curation of potential chimeric scaffolds) is less frequently employed these days and often super-scaffolding and error correction is undertaken with Hi-C methods only. Similarly the extensive manual curation of gene annotations and the impact that this had on the models is likely of more general interest (e.g. how many gene models were corrected, what type of errors were encountered?). Particularly also some mention of some of the specific challenges of this project (e.g. the need to combine multiple individuals to obtain sufficient quantities of gDNA) might be interesting for the readership.
The absence of line numbers is a little cumbersome for reviewing purposes, I'll below refer to specific parts of the text by page number (as printed on pdf document) - paragraph -line(within paragraph).
3-1-6: suggest changing "…. and included both laboratory and natural environmental conditions" to "…and have included…"
3-2-1: change "The first M. cinxia genome was released in 2014" to "The first M. cinxia draft genome" or "The first M. cinxia genome assembly"
Table1: reporting both GC and AT % is unnecessary. There are some discrepancies between the statistics reported for the chromosomal assembly in the Ahola et al (2014) paper vs this table. This may simply be due to different methods for assessing summary statistics (e.g. whether or not gaps are included by default), but warrants investigation/clarification. For example, the largest scaffold reported in the Ahola et al (2014) paper is 14,178,551bp. The description of the generation of a chromosomal build for the previous version indicates >280Mb were assigned to chromosomes, whereas the total assembly size in this table is reported to be only 251Mb.
6-2-2: What are the units for the cut-off (read length?)? If available, the data exploring the impact of different cut-offs on the assembly error rate could be of interest to others assembling genomes de novo.

6-2-6: As a specific example of a more general comment on number reporting, perhaps state 24.4 Gb instead of 24,409,505,551 bp? I am not sure that the precision is always necessary and scaling/rounding can help readability.
6-2-10: Are the alternative contigs extracted by default by the FALCON pipeline? Are there any adjustments that need to be made for an input of >1 individual, for example?
7-2-2: The raw data for the linkage map crosses, and also the RNAseq data for the transcriptome studies (on) is described as "unpublished", but I believe public sequence accessions are also being released with this manuscript. Is there additional information that would need to be disclosed for this information to be utilised by others or is the intention to highlight that the data will also be presented in upcoming publications?
7-2-6 "Part of" should be "Some of"
7-3-3: Specify "relative humidity" instead of RH.
Discuss why different approaches used for different RNAseq experiments.
8-1-5: Sequencing was "performed" rather than "made". Can you specify which HiSeq model and which sequencing library kit (or at the very least whether it was PCR-free)?
9-2-6: Presumably "de novo transcripts" refers to both transcriptomes 1 and 2, in which case I think it would be helpful to state this here. I assume the different analysis approaches for datasets 1 and 2 reflect different histories of the two datasets but it would be interesting to see some assessment of the relative performances of these approaches.
13-2-4: I think that http://butterflygenome.org would be sufficient for the URL here.
14-1-4: Are there any flow cytometry (or other) estimates of genome size that can be used to set alongside the v1 and v2 assembly sizes?

Level of Interest

Please indicate how interesting you found the manuscript: Choose an item.

Quality of Written English

Please indicate the quality of language in the manuscript: Choose an item.

Declaration of Competing Interests

Please complete a declaration of competing interests, considering the following questions:

- Have you in the past five years received reimbursements, fees, funding, or salary from an organisation that may in any way gain or lose financially from the publication of this manuscript, either now or in the future?
- Do you hold any stocks or shares in an organisation that may in any way gain or lose financially from the publication of this manuscript, either now or in the future?

- Do you hold or are you currently applying for any patents relating to the content of the manuscript?
- Have you received reimbursements, fees, funding, or salary from an organization that holds or has applied for patents relating to the content of the manuscript?
- Do you have any other financial competing interests?
- Do you have any non-financial competing interests in relation to this paper?

If you can answer no to all of the above, write 'I declare that I have no competing interests' below. If your reply is yes to any, please give details below.

I declare that I have no competing interests.

I agree to the open peer review policy of the journal. I understand that my name will be included on my report to the authors and, if the manuscript is accepted for publication, my named report including any attachments I upload will be posted on the website along with the authors' responses. I agree for my report to be made available under an Open Access Creative Commons CC-BY license (http://creativecommons.org/licenses/by/4.0/). I understand that any comments which I do not wish to be included in my named report can be included as confidential comments to the editors, which will not be published.

Choose an item.

To further support our reviewers, we have joined with Publons, where you can gain additional credit to further highlight your hard work (see: https://publons.com/journal/530/gigascience). On publication of this paper, your review will be automatically added to Publons, you can then choose whether or not to claim your Publons credit. I understand this statement.

Yes Choose an item.
