# Supplementary material for: Improved chromosome-level genome assembly of the Glanville fritillary butterfly (Melitaea cinxia) integrating Pacific Biosciences long reads and a high-density linkage map
Source: Gigascience. 2022 Jan 12;11:giab097. doi: 10.1093/gigascience/giab097 (PMC8756199; doi:10.1093/gigascience/giab097)
Supplement: giab097_Reviewer_2_Report_Original_Submission — Shanlin Liu -- 1/26/2021 Reviewed [file giab097_reviewer_2_report_original_submission.pdf]

Reviewer Report

Title: Improved chromosome-level genome assembly of the Glanville fritillary butterfly (Melitaea cinxia) integrating PacBio long reads and a high-density linkage map.

Version: Original Submission **Date:** 1/26/2021

Reviewer name: Shanlin Liu

Reviewer Comments to Author:

The authors presented us with an improved genome for Glanville fritillary butterfly. However, there are several issues that need to be addressed before its acceptance.

Major:

What the current manuscript lacks the most is the comparison between the improved genome assembly and its former version. Although the authors showed us an improved N50, I failed to find the explanations for several critical differences. For example, (1) the authors stated that ca. 90 MB additional assembly sequences were achieved, but no further information is available for those new sequences, are they redundancies or missed fragments in the version 1; (2) the improved genome predicted less genes compared to its former version, decreasing from ca. 16,000 genes to ~ 14,000 genes, which is contradictory to the aforementioned longer genome assembly; (3) the former genome version observed unevenly distributed repeat elements across chromosomes, while not in this improved one, which also needs explanations.

Another important issue of the present manuscript is the confusion introduced by varied genome assembly sizes. Firstly, the authors did not provide this critical information that can be estimated using several well-known methods, such as C value based on flow cytometry, or estimations based on kmer frequency information. Secondly, the author firstly mentioned that they sampled individuals that have low heterozygosity, but later the FALCON generated an assembly almost twice the size of the final genome. The authors may want to add extra analysis or words to clarify the genome size uncertainty.

Same to the above concern, Haplomerge seems an important step to obtain the final version assembly, and if I understand it correctly, the authors did not use a standardized analysis pipeline, please consider to include a schematic plot for your procedure to help readers better understand your steps and the principle behind them.

In addition, lots of methods are vaguely described, the authors should provide details for them to make sure the analyses are repeatable, e. g. on Page 6, the authors wrote: "This cut-off was experimentally found to give the best contiguity for the assembly, while minimizing (within a small margin of error) the percentage of possibly erroneous contigs". But I failed to find any details of their experiments. And on the same page, the authors checked putative chimerics manually, saying the error regions are with low coverage or repeat regions, the authors should give demonstration examples and statistics for different kinds of errors. Meanwhile, when they say the error regions were split, the authors should give details about how they determined the split positions since what they found are error regions instead of error bases. Also, on page 7, the authors stated "The contigs orders and orientations were manually fixed when needed", please list the different situations that meet your criteria.

The author may want to explain why they choose the 1,232 genes for manual annotation. Random?
Minor:
Remove "(e.g. Kahilainen et al. unpubl.)", it provides no useful information.
Table 1. N(%) of the verion 2 genome is zero? The scaffolding step does not introduce any Ns? I doubt that.
Page 5, please give the location information instead of a citation.
Page 7, please clarify the assembly version for raw read mapping, is it the one generated by FALCON with a genome size ~ 700 MB?
Page 9, "the first two step (bath A1 and bath A2)", please provide biological explanations.
Marey map needs citation and a brief explanation of its debut.
"In M. cinxia the repeats are placed in single chromosomes whereas in H. melpomene they are present in all chromosomes. " How does it help to show the power of long read assembly? Need explanation.
Page 10, how does Velvet apply a kmer size of 99 bp when you only have a read length as long as 85 bp?
Table 2 title: species name should be in format of italic.
Please give a full name for BUSCO in its first appearance.

Level of Interest

Please indicate how interesting you found the manuscript: Choose an item.

Quality of Written English

Please indicate the quality of language in the manuscript: Choose an item.

Declaration of Competing Interests

Please complete a declaration of competing interests, considering the following questions:

- Have you in the past five years received reimbursements, fees, funding, or salary from an organisation that may in any way gain or lose financially from the publication of this manuscript, either now or in the future?
- Do you hold any stocks or shares in an organisation that may in any way gain or lose financially from the publication of this manuscript, either now or in the future?
- Do you hold or are you currently applying for any patents relating to the content of the manuscript?
- Have you received reimbursements, fees, funding, or salary from an organization that holds or has applied for patents relating to the content of the manuscript?
- Do you have any other financial competing interests?
- Do you have any non-financial competing interests in relation to this paper?

If you can answer no to all of the above, write 'I declare that I have no competing interests' below. If your reply is yes to any, please give details below.

I declare that I have no competing interests

I agree to the open peer review policy of the journal. I understand that my name will be included on my report to the authors and, if the manuscript is accepted for publication, my named report including any attachments I upload will be posted on the website along with the authors' responses. I agree for my report to be made available under an Open Access Creative Commons CC-BY license (http://creativecommons.org/licenses/by/4.0/). I understand that any comments which I do not wish to be included in my named report can be included as confidential comments to the editors, which will not be published.

Choose an item.

To further support our reviewers, we have joined with Publons, where you can gain additional credit to further highlight your hard work (see: https://publons.com/journal/530/gigascience). On publication of this paper, your review will be automatically added to Publons, you can then choose whether or not to claim your Publons credit. I understand this statement.

Yes Choose an item.
