# Supplementary material for: Improved chromosome-level genome assembly of the Glanville fritillary butterfly (Melitaea cinxia) integrating Pacific Biosciences long reads and a high-density linkage map
Source: Gigascience. 2022 Jan 12;11:giab097. doi: 10.1093/gigascience/giab097 (PMC8756199; doi:10.1093/gigascience/giab097)
Supplement: giab097_Reviewer_2_Report_Revision_1 — Shanlin Liu -- 6/4/2021 Reviewed [file giab097_reviewer_2_report_revision_1.pdf]

Reviewer Report

Title: Improved chromosome-level genome assembly of the Glanville fritillary butterfly (Melitaea cinxia) integrating PacBio long reads and a high-density linkage map.

Version: Revision 1 **Date:** 6/4/2021

Reviewer name: Shanlin Liu

Reviewer Comments to Author:

I would like to thank the authors for responding to my previous comments. However, I still have some concerns:

The first and foremost one is how the authors obtained their genome assemblies step by step. The current version lacks several critical pieces of information to explicitly explain the details for assembly redundancy removal. As the authors showed in this revised version, they obtained a genome assembly of size 1.5 times larger than expected. I thought the haplomerge step played a critical role in removing those redundancies. However, the authors explained that this step only removed 10% (~ 30 MB) of those redundancies. I failed to find any other clues in the current version in decreasing the initial 700 MB genome assembly to the final 400 MB genome assembly. I believe the authors have to add all the relevant details.

English is not my mother tongue, but some parts of the manuscript do read a little bit weird. Let the editor decide whether a native speaker has to be invited to edit the manuscript carefully.

Others:

In table 1, <0.00 should be < 0.01

Line 62: longer Pacbio reads -> Pacbio long reads

Line 110: SMRT abbreviated for?

Line 175: remove the spaces.

Line 331 - 333: Is there any literature to support your statement? Why can't chromosomes harbour various amounts of repeat elements?

Figure 4: Figure legend overlapped with the figure.

Level of Interest

Please indicate how interesting you found the manuscript: Choose an item.

Quality of Written English

Please indicate the quality of language in the manuscript: Choose an item.

Declaration of Competing Interests

Please complete a declaration of competing interests, considering the following questions:

- Have you in the past five years received reimbursements, fees, funding, or salary from an organisation that may in any way gain or lose financially from the publication of this manuscript, either now or in the future?
- Do you hold any stocks or shares in an organisation that may in any way gain or lose financially from the publication of this manuscript, either now or in the future?
- Do you hold or are you currently applying for any patents relating to the content of the manuscript?
- Have you received reimbursements, fees, funding, or salary from an organization that holds or has applied for patents relating to the content of the manuscript?
- Do you have any other financial competing interests?
- Do you have any non-financial competing interests in relation to this paper?

If you can answer no to all of the above, write 'I declare that I have no competing interests' below. If your reply is yes to any, please give details below.

I declare that I have no competing interests

I agree to the open peer review policy of the journal. I understand that my name will be included on my report to the authors and, if the manuscript is accepted for publication, my named report including any attachments I upload will be posted on the website along with the authors' responses. I agree for my report to be made available under an Open Access Creative Commons CC-BY license (http://creativecommons.org/licenses/by/4.0/). I understand that any comments which I do not wish to be included in my named report can be included as confidential comments to the editors, which will not be published.

Choose an item.

To further support our reviewers, we have joined with Publons, where you can gain additional credit to further highlight your hard work (see: https://publons.com/journal/530/gigascience). On publication of this paper, your review will be automatically added to Publons, you can then choose whether or not to claim your Publons credit. I understand this statement.

Yes Choose an item.
